# Novel insights into iron metabolism by integrating deletome and transcriptome analysis in an iron deficiency model of the yeast *Saccharomyces cerevisiae*

**DOI:** 10.1186/1471-2164-10-130

**Published:** 2009-03-25

**Authors:** William J Jo, Jeung Hyoun Kim, Eric Oh, Daniel Jaramillo, Patricia Holman, Alex V Loguinov, Adam P Arkin, Corey Nislow, Guri Giaever, Chris D Vulpe

**Affiliations:** 1 Department of Nutritional Sciences and Toxicology, University of California, Berkeley, California 94720, USA; 2 Stanford Genome Technology Center, Stanford University, Palo Alto, California 94304, USA; 3 Department of Bioengineering, University of California, Berkeley, California 94720, USA; 4 Physical Biosciences Division, Lawrence Berkeley National Laboratory, Berkeley, California 94720, USA; 5 University of Toronto, Department of Molecular Genetics, Toronto, Ontario M5S3E1, Canada; 6 University of Toronto, Banting and Best Department of Medical Research, Toronto, Ontario M5S3E1, Canada; 7 University of Toronto, Department of Pharmaceutical Sciences, Toronto, Ontario M5S3E1, Canada; 8 University of Toronto, Donnelley Centre for Cellular and Biomolecular Research, Toronto, Ontario M5S3E1, Canada

## Abstract

**Background:**

Iron-deficiency anemia is the most prevalent form of anemia world-wide. The yeast *Saccharomyces cerevisiae *has been used as a model of cellular iron deficiency, in part because many of its cellular pathways are conserved. To better understand how cells respond to changes in iron availability, we profiled the yeast genome with a parallel analysis of homozygous deletion mutants to identify essential components and cellular processes required for optimal growth under iron-limited conditions. To complement this analysis, we compared those genes identified as important for fitness to those that were differentially-expressed in the same conditions. The resulting analysis provides a global perspective on the cellular processes involved in iron metabolism.

**Results:**

Using functional profiling, we identified several genes known to be involved in high affinity iron uptake, in addition to novel genes that may play a role in iron metabolism. Our results provide support for the primary involvement in iron homeostasis of vacuolar and endosomal compartments, as well as vesicular transport to and from these compartments. We also observed an unexpected importance of the peroxisome for growth in iron-limited media. Although these components were essential for growth in low-iron conditions, most of them were not differentially-expressed. Genes with altered expression in iron deficiency were mainly associated with iron uptake and transport mechanisms, with little overlap with those that were functionally required. To better understand this relationship, we used expression-profiling of selected mutants that exhibited slow growth in iron-deficient conditions, and as a result, obtained additional insight into the roles of *CTI6*, *DAP1*, *MRS4 *and *YHR045W *in iron metabolism.

**Conclusion:**

Comparison between functional and gene expression data in iron deficiency highlighted the complementary utility of these two approaches to identify important functional components. This should be taken into consideration when designing and analyzing data from these type of studies. We used this and other published data to develop a molecular interaction network of iron metabolism in yeast.

## Background

Iron-deficiency anemia affects hundreds of millions of people worldwide and is the most common form of anemia, particularly in developing countries, with young children and pregnant women being the most vulnerable [1]. Iron deficiency in infancy and early childhood is associated with behavioral and cognitive delays. The importance of iron to human health derives from its critical role in many metabolic reactions as an electron donor and acceptor [2]. On the other hand, when iron is present in excess, it can catalyze the formation of potentially toxic reactive oxygen radicals. Consequently, evolutionarily-conserved homeostatic mechanisms control the import, distribution, storage and detoxification of this essential metal in all organisms to prevent its deficiency and toxicity.

The budding yeast *Saccharomyces cerevisiae *has provided significant insight into iron homeostasis in eukaryotes, including humans. In iron deficiency, yeast undergoes a global metabolic reorganization that includes activation of iron transporters, recycling of heme iron, release of stored iron from the vacuole, and down-regulation of iron-dependent processes [3].

The transcription factor Aft1p plays a central role in the expression of multiple genes in response to iron deficiency, particularly of those involved in iron uptake, including the iron transporter gene *FTR1 *and the iron ferroxidase gene *FET3 *[4-6]. Aft2p, the paralog of Aft1p, is also involved in iron homeostasis and regulates a set of genes that are partially redundant with those targeted by Aft1p [7,8]. Between the two, Aft1p plays a more prominent role in the transcriptional regulation of iron regulon genes [9]. The mechanisms by which these transcription factors sense cellular iron status are not completely understood. In addition to the transcriptional up-regulation of iron uptake processes, yeast down-regulates iron-dependent processes to preserve intracellular iron. This is achieved by a post-transcriptional mechanism via the RNA-binding proteins Cth1p and Cth2p, which are up-regulated in iron deficiency [10,11]. Cth1p preferentially targets the degradation of mRNAs encoding components involved in mitochondrial function, while Cth2p does the same for those encoding iron homeostasis and iron-utilizing proteins [11]. On the other hand, in high iron conditions, degradation of Ftr1p and Fet3p is increased, constituting a further level of post-translational control in iron metabolism [12].

Genome-wide functional profiling can help identify additional components of iron metabolism in yeast. The systematic creation of yeast deletion strains for most of the approximately 6,000 open reading frames (ORFs) reported in this organism allows to profile its genome under conditions of interest [13]. This collection of mutants (the deletome) has been previously used to identify novel components of iron metabolism by evaluating the growth phenotype in iron-limited media of each strain individually [14-16]. In contrast, the parallel analysis of deletion mutants allows monitoring their growth simultaneously, thus providing a high-throughput way to evaluate growth. This approach takes advantage of two 20-base oligomer sequences (up and downs tags) found at the site of each ORF deletion and that are unique to each mutant strain [17]. The tags in the DNA allow the relative quantitation of each strain in the pool by a universal tag-amplification step, followed by hybridization to purpose-built microarrays which contain the complementary oligos to the tags. Hybridization intensities in the microarray correspond to the abundance of the deletion strains in the treatment pool and, when compared to a control, are indicative of the relative sensitivity of each strain to the selective growth condition [18].

Several studies have used this approach to profile the yeast genome under a variety of growth conditions, and we have previously used it to identify components in the cellular response to toxic concentrations of iron and copper [19]. In this report, we describe the functional profiling of the yeast genome by parallel analysis of homozygous mutants in iron-limited conditions. We characterized the phenotype of selected severely growth-inhibited strains by individual growth analysis in iron-limited media and by gene expression profiling. Furthermore, we conducted expression profiling of yeast wild type in iron deficiency and compared these results to those obtained by functional profiling. We show that these methods provide complementary perspectives on iron metabolism in yeast. Finally, we integrated our results with existing published data to create a molecular interaction network for iron metabolism in yeast.

## Methods

### Yeast strains and culture

Yeast strains were from the BY4741 and BY4743 backgrounds (Research Genetics, now Invitrogen, Carlsbad, CA). Growth was conducted in rich (YPD, 1% yeast extract, 2% peptone and 2% dextrose) or synthetic defined media (SD, 1.7 g/L yeast nitrogen base, 0.75 g/L complete supplement mixture, 5 g/L ammonium sulfate and 2% dextrose). Bathophenanthrolinebisulfonic acid (BPS) or ferrozine (Sigma Aldrich, Saint Louis, MO) were used at different concentrations to induce iron-deficient conditions.

### Growth assays

For assays in solid media, individual deletion mutant strains were first pre-treated overnight by growing on plates containing YPD agar ± 500 μM BPS or 1 mM ferrozine. Cells were then gently scraped from the agar surface, resuspended in SD media, and diluted to optical densities at 595 nm (OD_595_) of 0.0165, 0.00165, and 0.000165. A volume of 5 μl of each dilution was spotted onto YPD agar plates ± 100 μM BPS, which were then incubated at 30°C and evaluated for growth after 3 days.

For assays in liquid media, cells were pre-treated as described above and diluted to an OD_595 _of 0.0165 in 400 μl of synthetic defined media contained in wells of a 48-well plate with 0, 50, or 100 μM BPS. The 48-well plate was then incubated at 30°C in a shaking incubator and spectrophotometer (Tecan Genios, Männedorf, Switzerland), which recorded the OD_595 _every 15 minutes for 24 hours to monitor growth.

### Functional profiling of the yeast genome in iron deficiency

Three independent pools of homozygous yeast deletion mutants (BY4743 background) were grown in YPD media in the presence of BPS at concentrations of 75 and 100 μM. Pool growth, genomic DNA isolation, PCR and hybridization to TAG3 arrays (Affymetrix, Santa Clara, CA) were carried out as described previously [18]. The sensitive, unaffected, and resistant strains were identified based on an outlier approach previously used to identify differentially-expressed genes [20]. Each strain is represented in the microarray by four values of signal intensity (uptag sense, downtag sense, uptag antisense and downtag antisense) that were averaged to give a single value representing the growth for that particular strain. Direct sequencing of tags from the yeast deletion set subsequently identified unexpected discrepancies between the sequence of some tags and the original ones, although these differences do not appreciably affect signal [21].

Growth for each of the strains in the pools treated with BPS was compared against their growth in 16 control experiments in YPD media by calculating a log_2 _ratio of treated to untreated signal intensity (fitness score). The corresponding scatter plots were tested for linearity as described previously [20], and normalized by global normalization using chip median intensity. In these scatter plots, data points for treatment-sensitive strains fall below the line (log_2 _< 1) while treatment-resistant (refractory) strains fall above it (log_2 _> 1). Data points for unaffected strains fall near the line of equivalence (log_2 _close to 1). In order to identify "outlier strains", which are differentially sensitive or resistant to treatment, a confidence (p-value) was assigned to each strain by calculating simultaneous tolerance confidence intervals. Sensitive or resistant strains from each experiment were determined by using cut-offs for expected false-positives on average [20] rather than for p- or q-values to ease interpretation. Cut-offs for q- and p-values are monotonically and continuously interrelated with the cut-offs for expected false-positives on average. A strain was considered to be significantly affected by the treatment if it was "sensitive" or "resistant" at the above-mentioned p-value level in all 16 replicates simultaneously.

### Gene expression profiling of an iron deficiency model in yeast

Yeast cells were grown (with BPS treatment, if applicable) to mid-log phase and collected by centrifugation. Total RNA was isolated using the hot phenol protocol (microarrays.org, ) Poly(A)^+ ^RNA was prepared using Oligotex resin (Qiagen Inc., Valencia, CA) according to the manufacturer's protocol. cDNA was synthesized from 2 μg of poly(A)^+ ^RNA using RNA SuperScript II RNAse H-reverse transcriptase (Invitrogen) and oligo-dT primer in the presence of aminoallyl-labeled dUTP. cDNA was labeled by incubation with Cy3 or Cy5 fluorescent dyes (Amersham Pharmacia Biotech, Pittsburg, PA) for one hour at room temperature in 100 mM sodium carbonate buffer (pH 9.0). The Cy3- and Cy5-labeled cDNA were mixed and hybridized overnight at 65°C onto a yeast cDNA microarray containing 6,218 yeast ORFs (Berkeley Yeast Consortium and Nutritional Sciences and Toxicology Genomics Facility, University of California, Berkeley, CA). Hybridized microarrays were scanned on an ArrayWoRx biochip (Applied Precision, Issaquah, WA) and quantification of signals was performed using GenePix software version 3.01 (Axon Instruments, Union City, CA). The ratio of the signal intensity from untreated wild type versus mutant or treated wild type was normalized by total median intensity and differentially-expressed genes were identified as described before [20].

### Hierarchical cluster analysis

Raw data from published functional experiments conducted under different growth conditions [18], available at , was re-analyzed using the outlier detection method [20]. For each condition, two biologic replicates were compared to each of sixteen control experiments. Only the genes that were identified in the two replicates either as sensitive or resistant were used for analysis. In addition, published data for iron and copper overload conditions was included [22]. Clustering analysis was performed only on the genes identified after BPS treatments by using the Expression Profile data CLUSTering and analysis tool (EPCLUST, ), with average linkage (average distance, UPGMA) clustering based on Spearman's rank correlation-based distance.

### Analysis of interactions networks and functional enrichment

Genes that were significant in all three BPS functional profiling experiments were mapped onto the yeast interaction [23] and regulatory networks (, accessed on July 2007) [24] using Cytoscape version 6.0 . Active sub-networks showing significant changes in fitness after BPS treatment were identified with the Jactive modules plugin version 2.2 [25] using both search and annealing strategies.

Functional and expression profiling data sets (significant in all 3 and at least in 2 experiments, respectively) were verified for enrichment for any particular biological attribute by identifying significantly-enriched Gene Ontology (GO) categories using the Functional Specification resource (FunSpec ), with a hypergeometric distribution P-value cut-off of 0.01.

### Construction of the iron metabolism map for yeast

Iron metabolism genes together with their known physical and genetic interactions in yeast were used to generate an iron metabolism map. In addition, published genomic screens associated either directly or indirectly to iron metabolism were systematically collected from PubMed (, up until October 2008). Data from this study was included as well. All data were organized following the order [source -interaction type -target] into a spreadsheet and imported into Cytoscape version 6.0 for visualization. Two views of the iron map were generated. In the first view of the map, all data were organized using the force-directed layout. In the second view, data from high-throughput studies were excluded to facilitate visualization and the remaining nodes in the network were organized according to their involvement in iron-related pathways and/or cellular localization. Nodes and edges were color-coded to indicate differential growth/expression in BPS and the interaction type, respectively. Files are available upon request.

## Results and discussion

### Functional profiling of the yeast genome in iron deficiency identifies components of iron metabolism

The application of genome-wide expression profiling has accelerated our understanding of iron metabolism [6,26-29]. However, transcriptional and protein profiling methods provide limited insight into the functional importance of the differentially-regulated genes and do not differentiate between primary and downstream responses. In addition, gene expression approaches provide no information on functional components whose mRNA or protein levels are not directly regulated. In the case of iron metabolism, where there are several layers of regulation, particularly at the post-transcriptional level, analysis of gene expression could then present limitations.

For the functional profiling of the yeast genome, we grew three independent pools containing 4,757 homozygous yeast deletion strains in YPD media in the presence of 75 μM (n = 1) or 100 μM (n = 2) of the iron chelator BPS for 15 generations of growth. We then identified the strains with significant alterations in growth compared to regular rich media (as described in Methods). Because there were no differences between the strains identified at the two BPS concentrations tested, we combined the results from the three experiments. In this way, we identified a total of 141 and 20 strains that were sensitive and resistant to BPS in all three experiments, respectively [see Additional file 1]. Approximately 500 strains were affected in at least two of the treatments [see Additional file 2].

**Figure 1:**
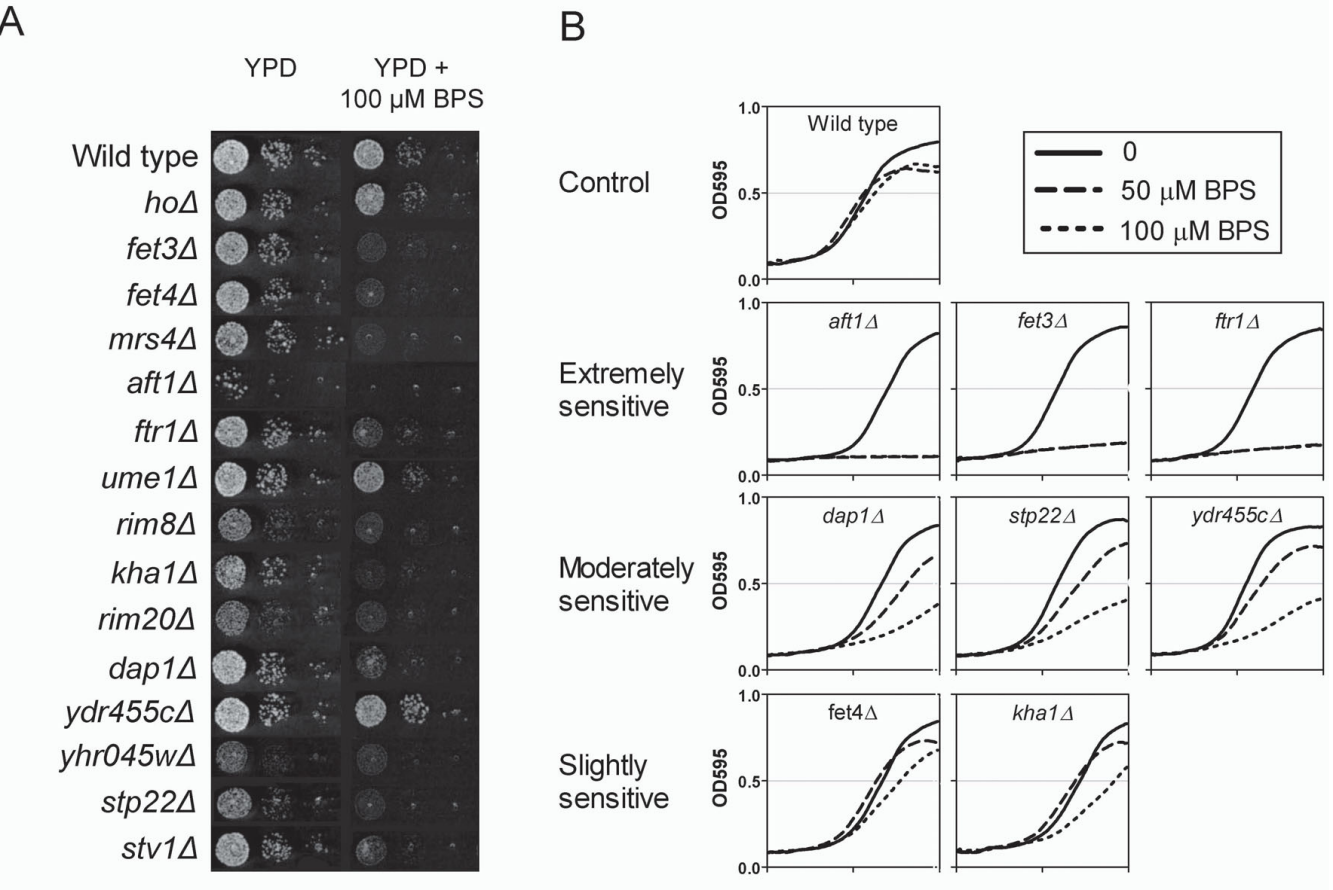
**Confirmatory assays of selected BPS-sensitive deletion strains identified by functional profiling**. The wild type strain and known mutants defective in iron metabolism, *aft1Δ *and *fet4Δ *(not identified in the functional studies), were included for comparison. **A **Growth assays in YPD agar plates. Cultures of individual deletion strains were pre-treated by growing overnight on BPS or ferrozine-containing plates, followed by serial dilution and spotting onto YPD plates ± 100 μM BPS. The strain *hoΔ *was included as a deletion control. **B **Growth assays in liquid SD media with 0, 50 or 100 μM BPS. Cultures were pre-treated and diluted in fresh media as before for the assay. Curves show the optical density at 595 nm (OD_595_) of the culture monitored for a period of 24 hours. Mutants exhibited different degrees of sensitivity to BPS, which is indicative of the relative importance of the missing gene in iron deficiency. Among these genes, *AFT1*, *FET3 *and *FTR1 *were the most essential for growth. The severity of the iron-deficient phenotype did not always correlate between liquid and solid media for the same strain (see results and discussion).

We selected a number of representative strains that showed growth sensitivity in iron-limited media for individual confirmatory analysis (Fig. 1). The wild type strain was modestly growth-inhibited at 50 and 100 μM of BPS, while the selected deletion strains exhibited different levels of sensitivity to treatment. Interestingly, the severity of the iron-sensitive phenotype did not always correlate between solid (agar) and liquid media for the same mutant. For example, *fet4Δ *exhibited a stronger growth defect on solid than in liquid media while *ydr055cΔ *appeared to be insensitive on solid. These discrepancies could be due to different media used in the assays (YPD for solid *versus *SD for liquid) or to an intrinsic feature of the growth matrix (solid *versus *liquid) such as nutrient availability, aeration, etc.

Among the highly required genes for optimal growth in BPS (corresponding to the 10 most sensitive strains in the screen), were genes that encode proteins known to be involved in iron metabolism (Table 1). Several identified genes encode proteins involved in high affinity uptake of elemental iron (*FTR1*, iron permease; and *FET3*, multi copper oxidase), and components of the copper assimilatory pathway (*ATX1*, chaperone;*GEF1*, vesicular chloride channel; and *CCC2*, P-type ATPase transporter). *CTR1*, the high-affinity transporter gene required for copper uptake, was essential for fitness in two of the three screens that we performed [see Additional file 2]. The identification of several copper metabolism genes could be related to the documented connection between the iron and copper metabolic pathways. Alternatively, this could be due to the fact that chelators are not entirely specific to a given metal, and BPS could also chelating copper in our system.

**Table 1:** Top 10 genes essential for optimal yeast growth in iron-deficient conditions induced by BPS treatment

**Systematic Name**	**Common Name**	**Protein Description**	**Average log_2 _Fitness^1^**
*YDR269C*		*CCC2 *antisense	-4.80

*YER145C*	*FTR1*	High affinity iron permease that forms a complex with Fet3p, expression regulated by iron	-4.72

*YNL259C*	*ATX1*	Cytosolic copper metallochaperone that transports copper to the secretory vesicle copper transporter Ccc2p for eventual insertion into Fet3p	-4.62

*YPL170W*	*DAP1*	Heme-binding protein involved in regulation of cytochrome P450 protein Erg11p, related to mammalian membrane progesterone receptors	-4.33

*YDR455C*		Dubious open reading frame, overlaps with *NHX1*	-4.30

*YMR058W*	*FET3*	Ferro-O2-oxidoreductase required for high-affinity iron uptake	-3.85

*YPL182C*		Dubious open reading frame, overlaps with *CTI6*	-3.74

*YPL181W*	*CTI6*	Relieves transcriptional repression by binding to the Cyc8p-Tup1p corepressor and recruiting the SAGA complex to the repressed promoter	-3.65

*YJR040W*	*GEF1*	Chloride channel localized to late- or post-Golgi vesicles, involved in iron metabolism	-3.65

*YKR052C*	*MRS4*	Mitochondrial iron transporter that functions under low-iron conditions	-3.55

^1^Average fitness scores for significantly sensitive strains in three replicate experiments. See Additional file 1 for complete list of genes which deletion affects growth in BPS (sensitive and resistant strains).

In spite of its known role in the induction of iron-uptake genes in iron-limited conditions, we did not identify the transcription factor gene *AFT1*. We noted high variability and generally low fitness of *aft1Δ *when grown on agar in the absence of BPS (Fig. 1A). However, this mutant grew relatively well in liquid media, although slower than the wild type (Fig 1B), and showed a marked growth inhibition when BPS was present. The reason why *AFT1 *was not identified in our screen with liquid media is unclear, but may be related to the intrinsic slow growth of the deletion strain relative to other ones in the pool, the competitive growth environment, and/or to a very low starting cell number in the pool.

Other genes with relatively high requirement in low-iron media (log_2 _fitness < -2.00, 13% of sensitive strains), include ones encoding components of the ESCRT-I complex (*STP22 *and *VPS28*, ubiquitin-dependent sorting of proteins into the endosome) and transcription factors (*OPI1 *and *IRS4*) [see Additional file 1]. The identification of genes involved in iron metabolism by functional profiling suggests that other identified genes, without any previous association to it, may play unknown roles in iron deficiency.

### Differences in iron-deficiency sensitivity profiles between functional profiling methods

The use of functional profiling with yeast deletion mutants to identify novel components in iron metabolism has been reported before, although by different approaches. There was low concordance between our results and those from these previous screens [see Additional file 3], which we attribute to the differences in BPS concentrations, growth matrixes (solid *versus *liquid media), and detection methods used. Furthermore, we grew all mutants simultaneously, creating a competitive growth environment which could have added more stress to the strains, as opposed to individual growth on plates in the published studies.

Davis-Kaplan *et al*. [14] performed a screen of diploid strains on solid media with 90 μM BPS and low μM concentrations of FeSO_4_, and scored growth after 24 h using a subjective scale. Strains with mutations in genes involved in high affinity iron transport, vacuolar acidification and others of diverse function were growth-inhibited. Of the 17 strains that exhibited a severe growth defect, only 4 associated with components of high affinity iron uptake(*fet3Δ, ftr1Δ*, *ccc2Δ *and *gef1Δ*) overlapped with our data.

Dudley *et al*. [15] also screened the same collection of mutants on agar plates as one of multiple chemical screens, using digital quantification of strain growth. They identified few of the known components of iron metabolism in yeast at a BPS concentration of 200 μM. Of the 35 "high confidence" sensitive strains reported in their data, only one (*gcs1Δ*)was common with our study.

Lesuisse *et al*. [16] conducted a screen of haploid strains grown on standard media in the presence of labeled ferrioxamine B (a siderophore). Unlike the previous two cases, strains with altered ferrioxamine B uptake were identified using a scintillation counter. Further characterization grouped the mutants into three categories with similar alterations in ferric reductase activity, iron uptake from ferrous salts and from siderophores. We found 12 strains (*aps3Δ, atx1 Δ, ccc2 Δ, rav1 Δ, rcy1 Δ, snf7 Δ, vps4 Δ, vps20 Δ, vps25 Δ, vps28 Δ, vps36 Δ *and *yme1Δ*) in common between our study and the 81 mutants with abnormally high or low uptake identified by them.

As noted previously, we found discrepancies in the sensitivity phenotype of some mutants after comparing their growth between solid and liquid media (Fig. 1). Together, these results support a rationale for screening in liquid media to uncover different and potentially novel components of iron metabolism in yeast.

### The importance of intracellular trafficking pathways and the vacuole for optimal growth in low-iron conditions

Interestingly, most of the genes that we identified by functional profiling encoded components involved in intracellular transport (n = 53, 33% of the dataset). Among these, were genes associated with organelle organization and biogenesis (peroxisome, n = 9; endosome, n = 11; and vacuole, n = 5), protein transport, and components of the endocytic pathway (Table 2) [see Additional file 4].

**Table 2:** Gene Ontology enrichment by biological process and cellular localization of genes identified by functional profiling

**BIOLOGICAL PROCESS**			**CELLULAR COMPONENT**		
	
**Category**	**p-value^1^**	**# genes^2^**	**Category**	**p-value^1^**	**# genes^2^**
Transport	1.87 E-14	53	Endosome	5.24 E-10	11

Organelle organization and biogenesis	2.44 E-12	41	Peroxisome	5.11 E-07	9

Peroxisome organization and biogenesis	6.98 E-12	11	Cytoplasm	3.94 E-06	73

Intracellular protein transport	3.11 E-11	32	Peroxisomal membrane	6.98 E-06	5

Protein transport	6.79 E-10	33	Intracellular	1.16 E-04	98

Protein-peroxisome targeting	4.41 E-09	7	Cell	1.75 E-04	102

Protein targeting	4.99 E-09	22	AP-3 adaptor complex	3.76 E-03	2

Cytoplasm organization and biogenesis	1.82 E-08	41	Vacuolar membrane	6.58 E-03	5

Vacuole organization and biogenesis	6.98 E-07	14	Hydrogen-translocating V-type ATPase complex	7.16 E-03	3

Protein-vacuolar targeting	1.90 E-06	10	Membrane	7.28 E-03	28

^1^Calculated from hypergeometric distribution P < 0.01

^2^Number of identified genes in the Gene Ontology (GO) category

Enriched categories were identified using Funspec; see Additional file 4 for complete tables.

Although the most sensitive strains contained deletions in genes directly involved in iron uptake and metabolism, they accounted for a small fraction, as the majority of genes were involved in vacuolar function. The likely reason for an overrepresentation of vacuolar genes is that the vacuole acts as an iron reservoir in yeast [30] and its function has been shown to be important for iron homeostasis [14,31-34]. In these lines, vacuolar acidification was an essential biological process, and genes of the H-V-type ATPase complex (*STV1*, *RAV1 *and *RAV2*) were necessary for optimal growth. Thus, this further supports an important role of a functional vacuole in iron deficiency.

Metal transport processes were also important for yeast growth under these conditions [see Additional file 4]. However, their relative importance given by functional enrichment was lower than the vacuole and intracellular transport, which had lower hypergeometric p-values (that is, they were more significant). Nevertheless, these results indicate an important and known role of iron uptake pathways in iron deficiency.

### Gene regulatory networks essential in iron-limited conditions

We analyzed the yeast molecular interaction network to find genes potentially working in a coordinated fashion in iron-limited conditions, but not necessarily associated to a same biological process or molecular function. For this purpose, we used the Jactive modules plug in for Cytoscape, which assigns statistically-determined scores to subnetworks and identify those ones with high scores [25]. After overlaying our functional data, the resulting high-scoring subnetworks were enriched with genes essential for growth in iron deficiency.

**Figure 2:**
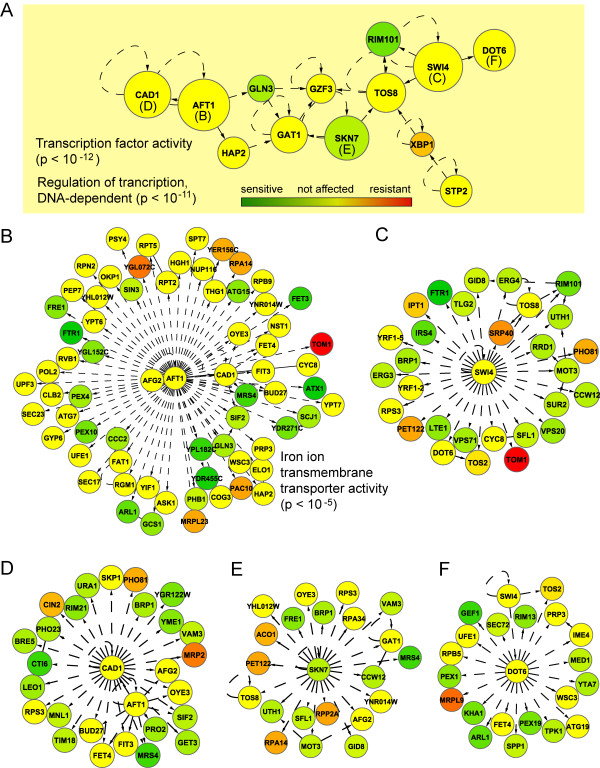
**Functional identification of transcriptional response networks in iron deficiency**. **A **Interaction network of highly interconnected nodes, mostly representing transcription factors, associated with the response to iron deficiency in yeast. Each node interacts with other genes (not shown) which deletions result in growth sensitivity to BPS. Node size is proportional to its connectivity to other nodes in the network, which were omitted for clarity purposes. Nodes with the highest number of interactions from **A **are shown with their corresponding transcriptional targets for **B ***AFT1*, **C ***SWI4*, **D ***CAD1*, **E ***SKN7 *and **F ***DOT6*. Most of these transcription factors were not identified by functional profiling although many of their gene targets were either essential or detrimental for yeast's growth in BPS. Enriched GO molecular functions or biological processes for each interaction subnetwork (if any) are indicated with corresponding hypergeometric p-values.

Using the search strategy in Jactive modules, we found 4 significant subnetworks (*z*-scores > 3). The merge of these networks (totaling 199 nodes) showed many highly interconnected nodes (or hubs), namely *AFT1*, *CAD1*, *DOT6*, *GAT1*, *GLN3*, *GZF3*, *HAP2*, *RIM101*, *SKN7*, *STP2, SWI4*, *TOS8*, and *XBP1 *(Fig. 2A). These genes encode transcription factors that regulate and/or physically interact with many significant genes from our data, some of which are involved in iron metabolism (Figs. 2B–F) [see Additional file 5]. Therefore, these transcription factors are likely to mediate the cellular adaptation and response to iron deficiency, although most of them were not significant in our screen (for example *AFT1 *and *CAD1*, both implicated in iron metabolism). The fact that the network analysis identified these additional functional components shows its importance as a complementary tool in genomic analysis.

Both Aft1p and Cad1p regulate many overlapping genes and appear to work in conjunction to mediate the transcriptional response of several genes in iron deficiency (Figs. 2B and 2D). Cad1p is required for stress response, growth at toxic levels of iron chelators, and thought to be involved in iron metabolism [35,36]. Most notably, Cad1p regulates *CTI6 *and *MRS4 *which were highly required in BPS, suggesting that its role in iron deficiency may be related to these interactions. Functionally-enriched biological processes of Cad1p-regulated genes that were significant in our study include histone deacetylation/chromatin modification (*SIF2*, *PHO23 *and *CTI6*) and response to arsenic (*GET3 *and *TIM18*). Thus, Cad1p may be involved in transcriptional activation in response to iron deprivation and other stressors.

Swi4p, Dot6p and Skn7p (Figs. 2C, E and 2F, respectively), on the other hand, regulate many genes with diverse functions so their relation to the regulation of specific processes is unclear. These transcription factors also showed partial overlap in regulated genes, indicating the existence of multiple transcriptional regulators for the genes that we identified as essential for optimal grow in BPS. This redundancy at the regulatory level could be the reason why several of these transcription factors were not identified in our functional screen (Fig 2A), as the lost of any of them in the deletion mutants could be compensated by the function of the remaining ones. Information about Swi4p, Dot6p and Skn7p, and their regulated genes, can be found at YEASTRACT .

### The function of the ESCRT complexes and peroxisome are required in iron deficiency

**Figure 3:**
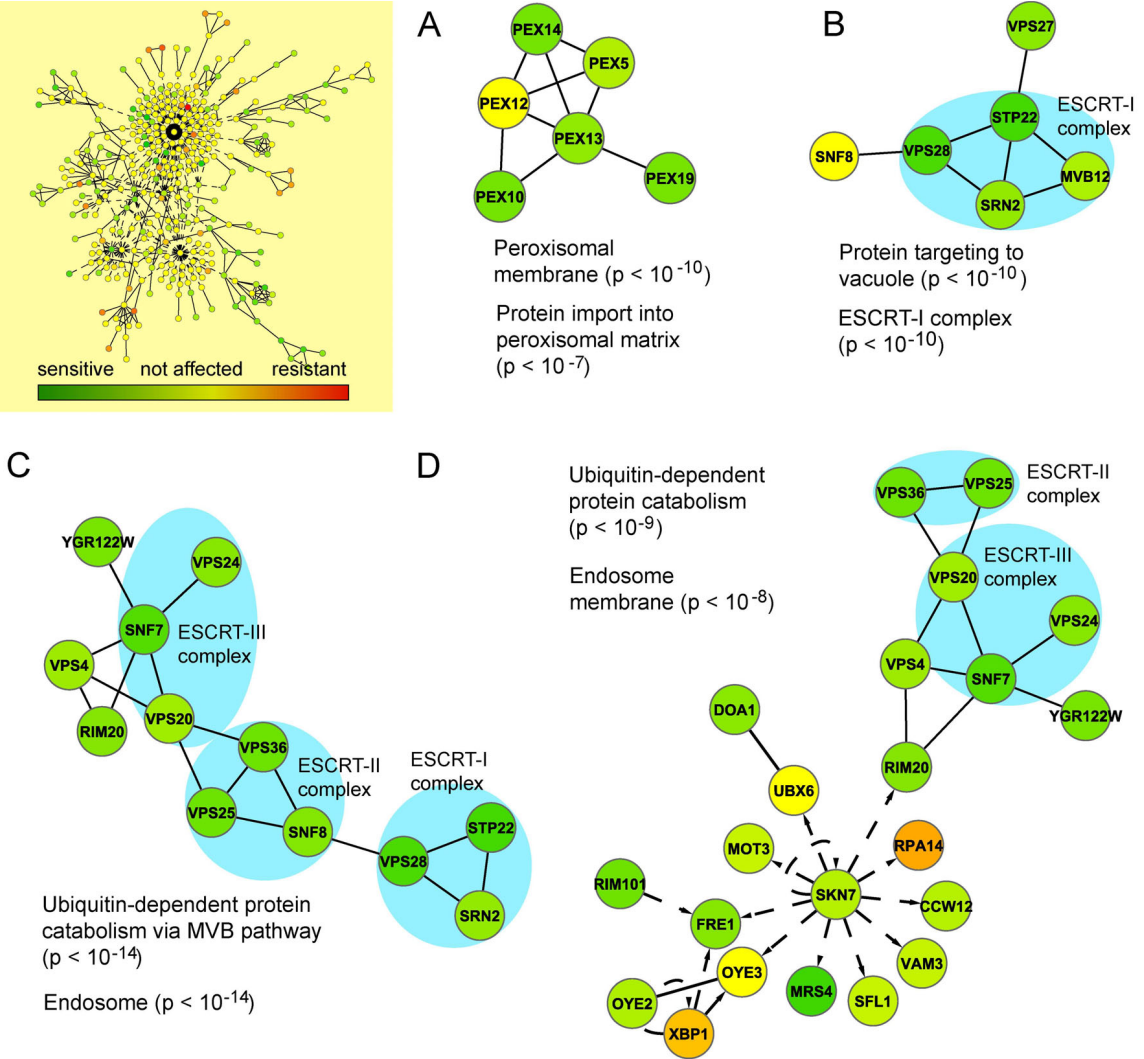
**Subnetworks with essential components for optimal yeast growth in iron deficiency**. The majority of proteins interact physically (solid lines) and form molecular complexes that include the **A **peroxisome, as well as **B**, **C**, **D **ESCRT I, II and III. Deletion of these subunits (green nodes) results in growth sensitivity in BPS, probably by disruption of the function of these complexes. Significant over represented GO categories are shown for each interaction network.

We also searched for essential subnetworks using the annealing strategy in JActive modules and identified one large subnetwork (n = 370, *z*-score = 16.633) (Fig. 3). A second application of the search algorithm revealed several subnetworks of interest with predominantly physical interactions; one associated with the peroxisome (Fig. 3A) and three with the ESCRT complexes (Figs. 3B–D). Most genes in these subnetworks were essential, showing an important role of these cellular components in iron-deficient conditions.

Our findings for the peroxisome suggest that this organelle may play a previously unsuspected role in iron metabolism. We speculate that iron deficiency may impair mitochondrial β-oxidation due to the significant iron requirements of the mitochondrial electron transport chain. Under these conditions, peroxisomal β-oxidation may play a greater role in energy metabolism and disruption of the peroxisome may prevent the utilization of this alternative energy source.

Similarly, the requirement of many genes encoding subunits of the ESCRT complexes I, II and III is a strong indication of their involvement in iron-deficient conditions. The ESCRT complexes have been associated with metal resistance including cadmium and iron overload [22,37]. These results support the role of the ESCRT complexes and intracellular transport in metal homeostasis in general.

### Specific genetic requirements of yeast for optimal growth in iron deficiency

**Figure 4:**
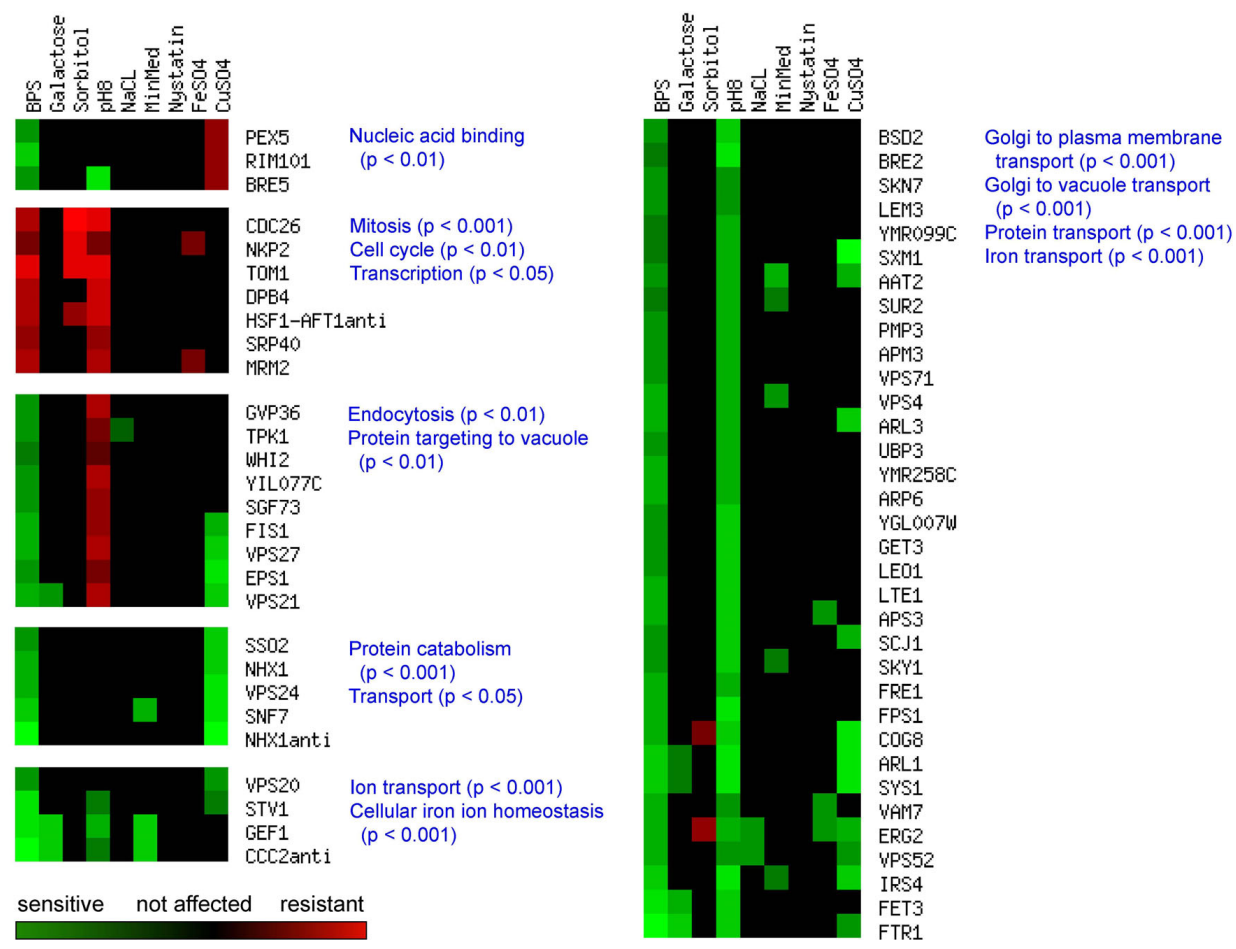
**Hierarchical clustering analysis of the genes identified by functional profiling in iron deficiency under diverse growth conditions**. Genes identified in iron deficiency were compared for functional requirement across different conditions from previously-published studies. Clusters contain genes with similar or opposite responses in these conditions to BPS treatment. Enriched GO biological processes for each cluster are indicated with corresponding hypergeometric p-values. Growth at pH 8 showed the closest similarity in genes to BPS treatment, with the majority of required genes associated to intracellular transport, but at the same time showed opposite requirements for a subgroup of genes also associated with this process.

We compared the genes that we identified after BPS treatments to the ones previously identified under growth in galactose, sorbitol, pH 8, NaCl, minimal media, nystatin, and iron and copper overload. We performed a cluster analysis with the 161 genes identified in our BPS treatments, as our goal was to provide insight into the genetic requirements in this condition and its specificity compared to other stressors (Fig. 4) [see Additional file 6]. Most genes required in iron deficiency were specific to it, although several ones involved in high affinity iron uptake (*FTR1, CCC2 *and *ATX1*)were also required for optimal growth in other conditions including galactose and high pH.

There were distinct groups of genes with similar and opposite requirement patterns in the different growth conditions (Fig. 4) [see Additional file 6]. Among the conditions that we compared, growth at pH 8 exhibited the closest similarity in the genetic requirements to BPS treatment, particularly in genes associated with the intracellular trafficking pathway and transport (p < 0.001). The presence of genes required in iron deficiency (*FTR1*, *FRE1 *and *FET3*) suggest low-iron conditions in pH 8 and could be associated to the low solubility of iron at high pH. However, because the genes involved in vacuolar/intracellular trafficking play diverse roles in yeast metabolism, it is not possible to establish whether the observed similarity between the pH 8 and BPS treatments is associated to iron deficiency. On the other hand, several genes with diverse functions including growth (*TPK1 *and *WHI2*) and protein targeting (*EPS1*, *VPS27 *and *VPS21*), showed an opposite requirement in pH 8.

Not unexpectedly, iron overload and deficiency showed very little overlap in their genetic requirements, which may implicate different homeostatic mechanisms under these two conditions. Interestingly, *CIN2 *displayed opposite requirements, being essential in iron overload but detrimental in iron deficiency. Cin2p is a GTPase-activating protein involved in microtubule stability. In mammalian cells, microtubules interact with the iron-storing protein ferritin, playing a role in its cytoplasmic distribution and possibly increasing its iron-binding capacity relative to microtubule-associated ferritin [38]. The role of Cin2p in yeast could be associated to iron homeostasis through its role in microtubule function.

Interestingly, copper overload exhibited concordance in the requirement of several genes associated with intracellular trafficking (for example,*VPS27 *and *VPS21*) and iron transport (*FTR1*), suggesting that excess copper (in addition to its deficiency) alters iron homeostasis.

Our results show a specific genetic requirement profile in iron deficiency as opposed to other environmental stressors. Few components of iron metabolism form part of yeast's response to other growth conditions, suggesting multiple functions for them or that these conditions can also alter iron homeostasis.

### Differential gene expression in iron deficiency indicates a metabolic reprogramming in yeast

We evaluated the transcriptional response of yeast in iron deficiency by gene expression profiling after treatment with 100 μM BPS for 1 hour. We conducted three independent experiments and considered for analysis those genes that were differentially-expressed in at least two of them. In this way, we identified a total of 100 and 42 up- and down-regulated genes, respectively [see Additional files 7 and 8]. Most of the up-regulated genes corresponded to iron (n = 17) and nucleotide (n = 15) metabolism. Among these two groups, iron metabolism genes had, on average, higher expression levels than those for nucleotide metabolism. This last group included transcription factor and chromatin remodeling genes and its presence indicate that nuclear changes precede and/or accompany adaptation to iron deficiency. On the other side, genes associated with energy (n = 5, all localized to the mitochondria), protein (n = 5), and nucleotide (n = 5) metabolism were down-regulated.

GO enrichment analysis showed that genes that were up-regulated were mainly associated with metal transport (iron, n = 11; copper, n = 4; and zinc, n = 3) and their products localized to the membrane (n = 38) and extracellular space (n = 6). On the other hand, down-regulated genes were associated with components of the TCA cycle (*ACO1*, *ACO2 *and *SDH4*) and most of their products localized to the mitochondria (*CYT2, CCP1 *and *COX17*) (Table 3) [see Additional file 9].

**Table 3:** Gene Ontology enrichment by biological process for differentially-expressed genes in BPS

**BIOLOGICAL PROCESSES WITH UP-REGULATED GENES**					
	
**Category**	**p-value^1^**	**#genes^2^**	**Category**	**p-value^1^**	**#genes^2^**
Ion transport	1.00 E-14	20	Oxidation reduction	1.38 E-03	11

Iron ion transport	4.47 E-13	11	Metal ion transport	1.55 E-03	3

Copper ion import	2.37 E-06	4	Zinc ion transport	2.16 E-03	3

Siderophore transport	2.62 E-06	3	Siderophore-iron transport	2.78 E-03	2

Cellular iron ion homeostasis	9.36 E-05	6	Biopolymer biosynthetic process	2.87 E-03	7

Copper ion transport	1.17 E-04	4	Response to starvation	2.90 E-03	3

Intracellular copper ion transport	2.07 E-04	3	High-affinity iron ion transport	3.85 E-03	2

Transport	2.4 E-04	28	Cation transport	5.40 E-03	3

Response to inorganic substance	1.13 E-03	2			

**BIOLOGICAL PROCESSES WITH DOWN-REGULATED GENES**					
	
**Category**	**p-value^1^**	**# genes^2^**	**Category**	**p-value^1^**	**#genes^2^**
Tricarboxylic acid cycle	5.25 E-03	3	Regulation of ergosterol	6.36 E-03	1
			biosynthetic process		

^1^Calculated from hypergeometric distribution P < 0.01

^2^Number of identified genes in the Gene Ontology (GO) category

Enriched categories were identified using Funspec; see Additional file 9 for complete tables.

We also observed in BPS a time-dependent increase in the number of up-regulated genes involved in high affinity iron transport and glycolysis, which occurred in parallel to an increase in the number of down-regulated genes associated with the TCA cycle, respiratory chain and biosynthesis of certain amino acids (data not shown). Up-regulation of genes in the glycolytic pathway probably occurs to compensate for the decreased energy production as a consequence of the down-regulation of components involved in aerobic energy synthesis.

Altogether, our data supports that iron deficiency induces metabolic changes that aims to preserve iron from non-essential cellular processes (reviewed by Kaplan *et al*. [3]). The down-regulation of several mitochondrial/energy metabolism genes suggests that during anaerobic growth in plain YPD, when glucose remains in the media, respiration genes are not entirely down-regulated (or not down-regulated at all) even though they are not required at that point of growth. In BPS, maintaining the expression of these genes is expensive in terms of iron usage and can be detrimental; therefore, yeast can afford to down-regulate them if glucose is still present. On the other side, up-regulation of iron uptake mechanisms aims to increase intracellular iron levels. Most of the up-regulated genes were associated with these processes, suggesting that iron uptake is mainly regulated at the transcriptional level. Shakoury-Elizeh *et al*. [6] also showed that yeast subjected to iron deprivation undergo a transcriptional remodeling, resulting in a metabolic shift from iron-dependent to iron-independent pathways, which includes biotin uptake and biosynthesis, nitrogen assimilation, and purine biosynthesis.

### Functional and expression profiling provide complementary views of yeast's response in iron deficiency

Of the 161 genes that we identified with functional profiling, only 11 were differentially-expressed (Table 4). Both functional and expression profiling of yeast grown in limited-iron conditions identified few known components of the iron regulon (*FET3*, *FRE1*, *MRS4 *and *CCC2*) as functionally essential and up-regulated, respectively. *YCR007C*, encoding a putative plasma membrane protein, was also essential and up-regulated in iron deficiency, and could be involved in iron uptake or a related process. *DAP1 *and *YTA7 *were also essential but down-regulated, although their requirement with decreased expression levels in iron deficiency is not clear. The aconitase gene *ACO1*, which product is a component of the TCA cycle, was down-regulated and its presence was detrimental for growth, thus further supporting the fact that reduction in aerobic respiration helps preserve iron for other vital processes.

**Table 4:** Overlapping genes identified by expression and fitness profiling approaches under iron-limited conditions

**Gene ID**	**Gene Name**	**Log_2 _expression**	**Log_2 _fitness**	**Protein function**	**Cellular localization**
*YCR007C*^1^		0.47	-0.98	Unknown	Plasma membrane

*YMR058W*^1^	*FET3*	0.76	-3.85	Multicopper oxidase	Plasma membrane

*YLR214W*^1^	*FRE1*	0.93	-2.21	Ferric (and cupric) reductase	Plasma membrane

*YKR052C*^1^	*MRS4*	0.50	-3.55	Mitochondrial carrier protein	Mitochondria

*YJL094C*^1^	*KHA1*	0.40	-2.51	Putative H+/K+ antiporter	Membrane

*YHL027W*^1^	*RIM101*	0.76	-2.64	Transcriptional activator required for meiosis	Nucleus

*YDR271C*^1^		0.82	-2.56	Unknown, overlaps *CCC2*	Unknown

*YDR270W*^1^	*CCC2*	0.63	-1.65	P-type ATPase	Golgi apparatus

*YPL182C*^2^		-0.29	-3.65	Cyc8-Tup1 interacting protein	Nucleus

*YPL170W*^2^	*DAP1*	-0.32	-4.33	Regulator of Erg11	Membrane

*YGR270W*^2^	*YTA7*	-1.54	-1.52	ATPase activity	Unknown

*YLR304C*^3^	*ACO1*	-0.82	1.03	Aconitase (TCA cycle)	Mitochondria

^1^Up-regulated and essential for optimal growth in iron deficiency

^2^Down-regulated and essential for optimal growth in iron deficiency

^3^Down-regulated and detrimental for optimal growth in iron deficiency

Although the number of identified genes was about the same, functional profiling identified primarily components of the intracellular trafficking pathway and vacuole (Table 2), while expression profiling identified most components in iron and metal metabolism (Table 3). Therefore, these approaches identified components that are functionally distinct but both important for iron homeostasis.

Low affinity- and siderophore-iron transport systems (*FET4, ARN and FIT *genes) are important for iron uptake and the fact that they were differentially-expressed after BPS treatment in our experiments, confirms a role for them in iron deficiency. However, we did not identify these same genes in our functional studies, which we attribute to functional redundancy. Additionally, deletion of the *ARN *and *FIT *genes would probably not be detrimental for growth because the pathway in which they are involved requires the presence of siderophores not produce by yeast itself but by other microorganisms. Therefore, the presence or absence of these genes would not have an impact on fitness under the growth conditions of our study, despite the fact that these genes were up-regulated. Conversely, the identification of several vacuolar-sorting defective mutants as sensitive suggests that intracellular trafficking pathways have components with specialized functions and low degree of functional redundancy. However, the encoded genes are non essential under regular conditions, as homozygous deletion mutants are viable in rich media. Intracellular transport and related processes are also required in a variety of cellular processes including detoxification of diverse heavy metals, so many of the genes involved may be constitutively expressed due to this continuous requirement and not differentially-expressed in iron deficiency.

Our analysis revealed two groups of genes essential for yeast in iron deficiency, with few overlapping members. One group that is inducible in response to low iron and is directly implicated in the metabolism of iron and other metals, with some degree of functional redundancy among components. The other group is not transcriptionally-regulated and forms part of regular metabolism and response to multiple stressors in yeast, and includes components associated with intracellular transport pathways.

The lack of correlation between genes identified by functional and expression profiling approaches has been reported previously [18,39,40], and may reflect a fundamental aspect of how cells respond to stress [41]. Haugen *et al*. [40] showed that genes that confer sensitivity to arsenic are in pathways that are upstream of the genes that are differentially-expressed in arsenic and that share redundant functions. This is also the case in iron deficiency, where some genes associated with intracellular trafficking function upstream of the ones involved in iron uptake. For example, copper loading onto Fet3p and assembly of functional Fet3p-Ftr1p in the plasma membrane for high-affinity iron uptake is dependent on these transport pathways. However, our results show that functional profiling does not necessarily identify genes in upstream pathways. Some other genes in intracellular transport pathways that we identified are associated to vacuolar iron storage and/or mobilization and hence, downstream of iron uptake. As such, these genes are not directly related to iron uptake but one that works in parallel to maintain iron homeostasis through the vacuole.

### Expression profiling of selected BPS-sensitive deletion strains confirms genes with roles in iron metabolism

The high requirement in BPS of the genes *DAP1*, *CTI6*, *MRS4 *and *YHR45W *suggested important roles for them, at the time of this study, in iron metabolism (Table 1) [see Additional file 1]. We expression profiled the mutants with deletion in these genes to gain insight into their functions under normal growth conditions [see Additional file 10]. While this study was in progress, transcriptional data for *cti6Δ *grown in the presence of BPS was published by Puig *et al*. [42]. Alterations in the expression of known iron metabolism genes in these strains compared to the wild type (both grown in rich media, YPD), as discussed below, implicates *DAP1*, *CTI6*, *MRS4 *and *YHR45W *in iron metabolism.

*CTI6 *encodes an Rpd3p-Sin3p histone deacetylase-associated protein involved in derepression of promoters which are repressed by Ssn6p-Tup1p complexes [43], and required for growth in iron deficiency [42]. Cti6p is recruited to iron-responsive promoters in an Aft1p-dependent manner and mediates the expression of *ARN1 *under heme-deficient conditions [44]. The *cti6Δ *mutant showed up-regulation of multiple genes, most notably ones involved in carbohydrate, nucleotide, metal and protein metabolism [see Additional file 11]. The most up-regulated gene was *MET17 *(log_2 _= 4.51), encoding an O-acetylhomoserine sulfhydrylase. Interestingly, *met17Δ *exhibits high levels of H_2_S and is resistant to lead and methylmercury, while moderate overexpression leads to decreased H_2_S levels [45]. Perhaps decreased levels of H_2_S in *cti6Δ *contribute to iron sensitivity. On the other hand, *cti6Δ *showed a dramatic down-regulation of a whole series of genes involved in response to pheromone and chemosensory perception, suggesting that these pathways are important in sensing or responding to iron deficiency.

*DAP1*, for damage resistance protein, encodes a 152 amino acid protein that belongs to the membrane-associated progesterone receptor (MAPR) family. The *dap1Δ *strain exhibits increased DNA damage particularly to methylating agents, telomere elongation, partial loss of ergosterol synthesis, and defect in mitochondrial biogenesis [46]. Dap1p functions in iron deficiency probably through the regulation of vacuolar structure via sterol synthesis, which is achieved by activation of Erg11p [47]. We found that *DAP1 *is down-regulated in iron deficiency and in its absence, some members of the iron regulon, particularly siderophore iron transporters, have decreased transcript levels (Table 5) [see Additional file 12]. In particular, *dap1Δ *showed decreased transcript levels of *FIT2-3 *and *ARN3-4*, encoding siderophore transporters, and *FET3 *involved in high affinity iron transport systems. Taken together, these results suggest a specific role of Dap1p in iron metabolism.

**Table 5:** List of iron homeostasis genes that are down-regulated in *dap1Δ*

**Gene ID**	**Gene Name**	**Log_2 _Expression^1^**	**Protein function**
*YEL065W*	*ARN3*	-0.77	Siderophore iron permease

*YLR136C*	*TIS11/CTH1*	-1.46	RNA binding protein involved in mRNA stability of genes encoding iron-containing proteins

*YMR058W*	*FET3*	-0.83	Cell surface ferroxidase, required for high-affinity ferrous iron uptake

*YOL158C*	*ARN4*	-1.29	Iron uptake via a siderophore enterobactin

*YOR382W*	*FIT2*	-2.33	Cell wall mannoprotein of iron transport facilitator

*YOR383C*	*FIT3*	-1.41	Cell wall mannoprotein of iron transport facilitator

*YOR384W*	*FRE5*	-0.81	Similar to Fre2p, ferric reductase

^1^Expression data represents the average of differentially-expressed genes in at least three out of four independent experiments.

Gene expression profiling was performed in *dap1Δ *and wild type strain grown in YPD media.

See Additional file 12 for list of all differentially-expressed genes in *dap1Δ*.

*MRS4 *encodes a mitochondrial iron transporter that functions under low-iron conditions [48], cooperating with its homologous *MRS3 *to provide iron for Fe-S clusters and heme biosynthesis in mitochondria [49,50]. The *mrs4Δ *strain showed up-regulation of an increased number of genes involved in carbohydrate and energy metabolism, and high affinity and siderophore-mediated iron uptake [see Additional file 13]. These results indicate a loss of mitochondrial functionality and that this mutant has to rely on other pathways, such as glycolysis, for energy production. Moreover, they suggest that mitochondria constitutes part of the iron-sensing mechanism in yeast, as low mitochondrial iron due to *MRS4 *deletion is perceived as an iron deficient-state despite growth in normal-iron conditions. The fact that *mrs4Δ *exhibited disturbances in iron metabolism shows that the functions performed by *MRS4 *are not fully redundant to that *MRS3 *as thought [48].

Lastly,*YHR045W *is an ORF of uncharacterized function. The *yhr045wΔ *mutant showed increased transcript levels of several genes involved in iron homeostasis such as *ARN3-4, FIT2-3*, *CCC2 *and *FET5 *(but curiously, not the entire iron regulon), as well as multiple genes involved in energy metabolism including amino acid and carbohydrate metabolism. On the other hand, the majority of genes with decreased transcript levels encode ribosomal proteins [see Additional file 14]. In contrast to *cti6Δ*, we did not observe a decrease in transcript levels of chemosensory perception in *yhr045wΔ*, suggesting that this mutant is sensitive to low-iron media because of a specific defect in iron metabolism rather than general external stimuli sensory system.

### The iron metabolism map in yeast

Using information from known molecular interactions associated to iron metabolism, we created a network to further understand the interactions between its different components. We first constructed a network with all interactions and organized it with the force-directed algorithm in Cytoscape [see Additional file 15]. Here, nodes in the network behave as electrons that repulse each other while edges behave as springs pulling connected nodes closer to each other. The result is a graphic where all components in the system are in equilibrium, that is, the sum of forces is decreased to a minimum. By applying this layout, we found a cluster of nodes near of the center of the network that were highly interconnected and enriched with known iron metabolism genes including *AFT1*. *CTH2 *(*TIS11*), which protein product targets degradation of mRNAs from iron metabolism-related genes, is closer to the cluster, while its homolog *CTH1 *targeting mitochondrial genes, is located towards the outside of the network. Among the genes that we studied by expression profiling of deletion strains, *DAP1 *was the closest to it followed by *MRS4*, indicating a close functional relationship of these genes to iron metabolism. *CTI6 *and *YHR045W *were farther away from the iron metabolism cluster, suggesting less specialized functions but nonetheless important to iron metabolism.

We also constructed a second network that excluded molecular interactions derived from genomics data from this and other studies [11,22] [see Additional file 16]. A discrete number of genes in iron-protein utilizing pathways showed differential expression and/or functional requirement, suggesting that most of yeast's response in iron deficiency involve different pathways other than these. Iron metabolism is highly conserved in yeast and humans. In this network containing genes directly associated with iron metabolism, as well as iron related pathways, 44% of proteins have homologs in humans (SGD Model Organism BLASTP Best Hits). This value is higher than the estimated 33% of conservation between the human and yeast genomes (at a BLAST E-value of 10^-12^, which means highly conserved homologous genes) [51]. Therefore, these conserved genes and pathways associated to iron in yeast could help to better understand iron metabolism in humans.

## Conclusion

We analyzed in parallel the collection of yeast homozygous deletion mutants under iron-limiting conditions in order to identify important components of iron metabolism. Using this approach, we identified several known gene products required for high-affinity free iron uptake, suggesting that additional genes identified with this method may play important roles in cellular iron metabolism. A considerable fraction of these genes were associated with intracellular trafficking and transport and differed from those identified by gene expression profiling under the same conditions, where most of the genes were involved in iron uptake.

Giaever *et al*. [18] and Birrell *et al*. [39] first observed the lack of correlation between fitness and expression profiling methods in a diversity of growth conditions. In this report, we showed that expression and functional profiling identified different components of iron metabolism in yeast. Our results illustrate that the information provided by these approaches complement each other and can help gain a global view of cellular processes that operate in yeast to maintain iron homeostasis under low-iron conditions. Understanding the effects of iron deficiency at the cellular level can provide a better insight into the mechanisms and detrimental effects of iron-deficiency anemia in humans.

## Authors' contributions

WJ analyzed the data, created the iron map and prepared the manuscript. JK performed the expression profiling experiments, analyzed the data and created the iron map. EO and DJ performed the functional profiling experiments and growth assays. PH designed and printed the microarrays for expression profiling studies. AL performed the statistical analysis. CV and EO helped to draft the manuscript. AA, CN, GG and CV conceived the study, and participated in its design and coordination. All authors read and approved the final manuscript.

## Supplementary Material

Additional file 1 **List of all genes that were identified by functional profiling in three out of three independent experiments.** Deletion of these genes resulted in significant growth alterations in the presence of BPS compared to YPD media. Click here for file

Additional file 2 **List of all genes that were identified by functional profiling in at least two out of three independent experiments.** Deletion of these genes resulted in significant growth alterations in the presence of BPS compared to YPD media. Click here for file

Additional file 3 **Comparison of yeast functional data in iron deficiency between this and other published studies.** Genes identified in this study y functional profiling were compared to those reported by Davis-Kaplan *et al*. (2004), Dudley *et al*. (2005) and Lesuisse *et al*. (2005). Click here for file

Additional file 4 **Gene Ontology enrichment analysis of functional profiling data.** Gene Ontology molecular functions, biological processes and cellular components that were significantly enriched with genes identified by functional profiling. Click here for file

Additional file 5 **Functional identification of transcriptional response networks in iron deficiency.** All 13 hubs from figure 2A are individually shown with their transcriptional targets, colored in green if deletion of the gene resulted in mutant strain sensitivity to BPS, or red if resistance. Click here for file

Additional file 6 **Hierarchical clustering analysis of the genes identified by functional profiling in iron deficiency under diverse growth conditions.** Cluster shows the reanalyzed data from previous functional studies of yeast mutants compared to data from this study. Click here for file

Additional file 7 **Differentially-expressed genes in nutritional iron-deficient yeast model.** Yeast wild type was treated with 100 μM BPS for 1 hour. Genes that were up or down-regulated in at least two out of three independent experiments are listed with average log_2 _expression values. Click here for file

Additional file 8 **Differentially-expressed genes in nutritional iron-deficient yeast model, data for each of three independent experiments.** Yeast wild type was treated with 100 μM BPS for 1 hour. Genes that were up or down-regulated in any of three independent experiments are listed with log_2 _expression values. Click here for file

Additional file 9 **Gene Ontology enrichment analysis of expression profiling data for up- and down-regulated genes.** Gene Ontology molecular functions, biological processes and cellular components that were significantly enriched with genes identified by expression profiling. Click here for file

Additional file 10 **Summary of differentially-expressed genes from individual experiments in the *dap1Δ*, *mrs4Δ *and *yhr045wΔ*.** Gene expression profiling was performed in the deletion mutants and wild type strain grown in YPD media. Click here for file

Additional file 11 **List of differentially-expressed genes in *cti6Δ*.** Gene expression profiling was performed in *cti6Δ *and wild type strain grown in YPD media. Cti6p relieves transcriptional repression by binding to the Cyc8p-Tup1p corepressor and recruiting the SAGA complex to repressed promoters. Click here for file

Additional file 12 **List of differentially-expressed genes in the *dap1Δ*.** Gene expression profiling was performed in *dap1Δ *and wild type strain grown in YPD media. Dap1p is a heme-binding protein involved in regulation of cytochrome P450 protein Erg11p, related to mammalian membrane progesterone receptors. Click here for file

Additional file 13 **List of differentially-expressed genes in *mrs4Δ*.** Gene expression profiling was performed in *mrs4Δ *and wild type strain grown in YPD media. Mrs4p is a mitochondrial iron transporter that functions under low-iron conditions. Click here for file

Additional file 14 **List of differentially-expressed genes in the *yhr045wΔ*.** Gene expression profiling was performed in *yhr045wΔ *and wild type strain grown in YPD media. Yhr045wp is a putative protein of unknown function. Click here for file

Additional file 15 **Yeast iron map organized by the force-directed layout in Cytoscape.** Map was constructed using all molecular interactions associated to iron metabolism compiled from the literature, including ones from the present study. The iron metabolism cluster, enriched with known genes associated to it, is circled in red. Genes in this cluster are highly interconnected with each other by edges that represent different molecular interactions retrieved from the literature. Specific genes can be searched in the map by using the Find feature in Adobe Reader or Acrobat. Click here for file

Additional file 16 **Yeast iron map organized by iron-related pathway and Gene Ontology cellular localization.** Map was constructed using molecular interactions associated to iron metabolism compiled from the literature, excluding those ones obtained from genomic screens. The map can be browsed from the main menu by cellular component or iron-related pathway. Specific genes can be searched in the map by using the Find feature in Adobe Reader or Acrobat. Click here for file
