# Supplementary material for: Novel insights into iron metabolism by integrating deletome and transcriptome analysis in an iron deficiency model of the yeast Saccharomyces cerevisiae
Source: BMC Genomics. 2009 Mar 25;10:130. doi: 10.1186/1471-2164-10-130 (PMC2669097; doi:10.1186/1471-2164-10-130)
Supplement: Additional file 1 — List of all genes that were identified by functional profiling in three out of three independent experiments. Deletion of these genes resulted in significant growth alterations in the presence of BPS compared to YPD media. [file 1471-2164-10-130-S1.pdf]

Additional File 1: Genes identified by functional profiling in three independent replicate experiments (n = 161). Deletion of these genes affects the fitness of mutant strains in iron deficiency. The requirement for growth of each gene was quantified as a $\log_2$ ratio of growth of the corresponding mutant strain in YPD with BPS compared to YPD alone. Values represent the average of the three experiments. A negative value indicates better growth in YPD (sensitive to BPS treatment) while a positive one indicates better growth in BPS (resistant to BPS treatment). Individual strain phenotypes were confirmed only for a small subset of genes from the table.

**Systematic Name**	**Common Name**	**$\log_2$ Fitness**	**Gene Description**
YDR269C		-4.80	Dubious open reading frame unlikely to encode a functional protein, based on available experimental and comparative sequence data
YER145C	*FTR1*	-4.72	High affinity iron permease involved in the transport of iron across the plasma membrane; forms complex with Fet3p; expression is regulated by iron
YNL259C	*ATX1*	-4.62	Cytosolic copper metallochaperone that transports copper to the secretory vesicle copper transporter Ccc2p for eventual insertion into Fet3p, which is a multicopper oxidase required for high-affinity iron uptake
YPL170W	*DAP1*	-4.33	Heme-binding protein involved in regulation of cytochrome P450 protein Erg11p; damage response protein, related to mammalian membrane progesterone receptors; mutations lead to defects in telomeres, mitochondria, and sterol synthesis
YDR455C		-4.30	Dubious open reading frame unlikely to encode a protein, based on available experimental and comparative sequence data; partially overlaps the verified gene YDR456W
YMR058W	*FET3*	-3.85	Ferro-O2-oxidoreductase required for high-affinity iron uptake and involved in mediating resistance to copper ion toxicity, belongs to class of integral membrane multicopper oxidases
YPL182C		-3.74	Dubious open reading frame unlikely to encode a protein, based on available experimental and comparative sequence data; partially overlaps the verified gene CTI6/YPL181W
YPL181W	*CTI6*	-3.65	Protein that relieves transcriptional repression by binding to the Cyc8p-Tup1p corepressor and recruiting the SAGA complex to the repressed promoter; contains a PHD finger domain
YJR040W	*GEF1*	-3.65	Chloride channel localized to late- or post-Golgi vesicles, involved in iron metabolism; highly homologous to voltage-gated chloride channels in vertebrates
YKR052C	*MRS4*	-3.55	Mitochondrial iron transporter of the mitochondrial carrier family (MCF), very similar to and functionally redundant with Mrs3p; functions under low-iron conditions; may transport other cations in addition to iron
YJR033C	*RAV1*	-3.52	Subunit of the RAVE complex (Rav1p, Rav2p, Skp1p), which promotes assembly of the V-ATPase holoenzyme; required for transport between the early and late endosome/PVC and for localization of TGN membrane proteins; potential Cdc28p substrate

Systematic Name	Common Name	Log_2 Fitness	Gene Description
YCL008C	*STP22*	-3.45	Component of the ESCRT-I complex, which is involved in ubiquitin-dependent sorting of proteins into the endosome; homologous to the mouse and human Tsg101 tumor susceptibility gene; mutants exhibit a Class E Vps phenotype
YPL065W	*VPS28*	-3.28	Component of the ESCRT-I complex, which is involved in ubiquitin-dependent sorting of proteins into the endosome; involved in transport of precursors for soluble vacuolar hydrolases from the late endosome to the vacuole
YHL020C	*OPI1*	-3.28	Transcriptional regulator of a variety of genes; phosphorylation by protein kinase A stimulates Opi1p function in negative regulation of phospholipid biosynthetic genes; involved in telomere maintenance
YMR054W	*STV1*	-3.22	Subunit of vacuolar-ATPase V0 domain, one of two isoforms (Stv1p and Vph1p); Stv1p is located in V-ATPase complexes of the Golgi and endosomes while Vph1p is located in V-ATPase complexes of the vacuole
YLR025W	*SNF7*	-3.18	One of four subunits of the endosomal sorting complex required for transport III (ESCRT-III); involved in the sorting of transmembrane proteins into the multivesicular body (MVB) pathway; recruited from the cytoplasm to endosomal membranes
YIR033W	*MGA2*	-3.08	ER membrane protein involved in regulation of OLE1 transcription, acts with homolog Spt23p; inactive ER form dimerizes and one subunit is then activated by ubiquitin/proteasome-dependent processing followed by nuclear targeting
YKR019C	*IRS4*	-3.01	Protein involved in regulation of phosphatidylinositol 4,5-bisphosphate concentrations; Irs4p and Tax4p bind and activate the phosphatase Inp51p; mutation confers an increase in rDNA silencing
YGL045W	*RIM8*	-2.99	Protein of unknown function, involved in the proteolytic activation of Rim101p in response to alkaline pH; has similarity to A. nidulans PalF
YBR164C	*ARL1*	-2.88	Soluble GTPase with a role in regulation of membrane traffic; regulates potassium influx; G protein of the Ras superfamily, similar to ADP-ribosylation factor
YPL139C	*UME1*	-2.85	Negative regulator of meiosis, required for repression of a subset of meiotic genes during vegetative growth, binding of histone deacetylase Rpd3p required for activity, contains a NEE box and a WD repeat motif; homologous with Wtm1p, Wtm2p
YHR045W		-2.71	Putative protein of unknown function; green fluorescent protein (GFP)-fusion protein localizes to the endoplasmic reticulum
YJL211C		-2.70	Dubious open reading frame unlikely to encode a protein, based on available experimental and comparative sequence data; partially overlaps the verified gene YJL210W/PEX2
YLR417W	*VPS36*	-2.68	Component of the ESCRT-II complex; contains the GLUE (GRAM Like Ubiquitin binding in EAP45) domain which is involved in interactions with ESCRT-I and ubiquitin-dependent sorting of proteins into the endosome
YJR102C	*VPS25*	-2.66	Component of the ESCRT-II complex, which is involved in ubiquitin-dependent sorting of proteins into the endosome
YHL027W	*RIM101*	-2.64	Transcriptional repressor involved in response to pH and in cell wall construction; required for alkaline pH-stimulated haploid invasive growth and sporulation; activated by proteolytic processing; similar to A. nidulans PacC
YDR203W		-2.64	Dubious open reading frame unlikely to encode a functional protein, based on available experimental and comparative sequence data

Systematic Name	Common Name	Log_2 Fitness	Gene Description
YJL004C	*SYS1*	-2.61	Integral membrane protein of the Golgi required for targeting of the Arf-like GTPase Arl3p to the Golgi; multicopy suppressor of ypt6 null mutation
YDR202C	*RAV2*	-2.60	Subunit of RAVE (Rav1p, Rav2p, Skp1p), a complex that associates with the V1 domain of the vacuolar membrane (H+)-ATPase (V-ATPase) and promotes assembly and reassembly of the holoenzyme
YDL118W		-2.60	Non-essential protein of unconfirmed function; mutants are defective in telomere maintenance, and are synthetically sick or lethal with alpha-synuclein
YGL153W	*PEX14*	-2.57	Peroxisomal membrane protein that is a central component of the peroxisomal protein import machinery, interacts with PTS1 (Pex5p) and PTS2 (Pex7p) peroxisomal matrix protein signal recognition factors and membrane receptor Pex13p
YMR057C		-2.56	Dubious open reading frame unlikely to encode a protein, based on available experimental and comparative sequence data; partially overlaps verified ORF AAC1
YDR271C		-2.56	Hypothetical protein
YDR265W	*PEX10*	-2.56	RING finger peroxisomal membrane peroxin required for peroxisomal matrix protein import, interacts with Pex12p, links ubiquitin-conjugating Pex4p to protein import machinery; mutations in human homolog cause a variety of peroxisomal disorders
YJL094C	*KHA1*	-2.51	Putative K+/H+ antiporter with a probable role in intracellular cation homeostasis, localized to Golgi vesicles and detected in highly purified mitochondria in high-throughput studies
YDL065C	*PEX19*	-2.50	Chaperone and import receptor for newly-synthesized class I peroxisomal membrane proteins (PMPs), binds PMPs in the cytoplasm and delivers them to the peroxisome for subsequent insertion into the peroxisomal membrane
YGL045W	*YGL045W*	-2.49	Protein of unknown function, involved in the proteolytic activation of Rim101p in response to alkaline pH; has similarity to A. nidulans PalF
YGR122W		-2.45	Putative protein of unknown function; deletion mutants do not properly process Rim101p and have decreased resistance to rapamycin; green fluorescent protein (GFP)-fusion protein localizes to the cytoplasm
YDL119C		-2.39	Putative mitochondrial transport protein; GFP-fusion protein is induced in response to the DNA-damaging agent MMS; the authentic, non-tagged protein is detected in purified mitochondria
YBR035C	*PDX3*	-2.38	Pyridoxine (pyridoxamine) phosphate oxidase, has homologs in E. coli and Myxococcus xanthus; transcription is under the general control of nitrogen metabolism
YLL043W	*FPS1*	-2.36	Plasma membrane glycerol channel, member of the major intrinsic protein (MIP) family of channel proteins; involved in efflux of glycerol and in uptake of the trivalent metalloids arsenite and antimonite
YML071C	*COG8*	-2.34	Component of the conserved oligomeric Golgi complex (Cog1p through Cog8p), a cytosolic tethering complex that functions in protein trafficking to mediate fusion of transport vesicles to Golgi compartments
YMR063W	*RIM9*	-2.30	Protein of unknown function, involved in the proteolytic activation of Rim101p in response to alkaline pH; has similarity to A. nidulans PalI; putative membrane protein
YPL002C	*SNF8*	-2.30	Component of the ESCRT-II complex, which is involved in ubiquitin-dependent sorting of proteins into the endosome; appears to be functionally related to SNF7; involved in glucose derepression

Systematic Name	Common Name	Log_2 Fitness	Gene Description
YDR329C	*PEX3*	-2.30	Peroxisomal membrane protein (PMP) required to recruit Pex19p chaperone to peroxisomes; plays selective, essential, direct role in PMP import as a docking factor for Pex19p
YOR089C	*VPS21*	-2.29	GTPase required for transport during endocytosis and for correct sorting of vacuolar hydrolases; localized in endocytic intermediates; detected in mitochondria; geranylgeranylation required for membrane association; mammalian Rab5 homolog
YOR275C	*RIM20*	-2.25	Protein involved in proteolytic activation of Rim101p in response to alkaline pH; member of the PalA/AIP1/Alix family; interacts with the ESCRT-III subunits Snf7p, suggesting a relationship between the response to pH and multivesicular body formation
YGL148W	*ARO2*	-2.22	Bifunctional chorismate synthase and flavin reductase, catalyzes the conversion of 5-enolpyruvylshikimate 3-phosphate (EPSP) to form chorismate, which is a precursor to aromatic amino acids
YLR214W	*FRE1*	-2.21	Ferric reductase and cupric reductase, reduces siderophore-bound iron and oxidized copper prior to uptake by transporters; expression induced by low copper and iron levels
YGL152C		-2.20	Dubious open reading frame unlikely to encode a protein, based on available experimental and comparative sequence data; partially overlaps the verified ORF PEX14/YGL153W
YKL041W	*VPS24*	-2.19	One of four subunits of the endosomal sorting complex required for transport III (ESCRT-III); forms an ESCRT-III subcomplex with Did4p; involved in the sorting of transmembrane proteins into the multivesicular body (MVB) pathway
YKL213C	*DOA1*	-2.18	WD repeat protein required for ubiquitin-mediated protein degradation, forms complex with Cdc48p, plays a role in controlling cellular ubiquitin concentration; also promotes efficient NHEJ in postdiauxic/stationary phase
YNR006W	*VPS27*	-2.15	Endosomal protein that forms a complex with Hse1p; required for recycling Golgi proteins, forming lumenal membranes and sorting ubiquitinated proteins destined for degradation; has Ubiquitin Interaction Motifs which bind ubiquitin (Ubi4p)
YPR024W	*YME1*	-2.15	Subunit, with Mgr1p, of the mitochondrial inner membrane i-AAA protease complex, which is responsible for degradation of unfolded or misfolded mitochondrial gene products; mutation causes an elevated rate of mitochondrial turnover
YMR202W	*ERG2*	-2.15	C-8 sterol isomerase, catalyzes the isomerization of the delta-8 double bond to the delta-7 position at an intermediate step in ergosterol biosynthesis
YKL197C	*PEX1*	-2.14	AAA-family ATPase peroxin required for peroxisome biogenesis, contains two 230 amino acid ATP-binding AAA cassettes, upregulated in anaerobiosis; Pex1p and Pex6p interact via their N-terminal AAA-cassettes
YMR154C	*RIM13*	-2.11	Calpain-like protease involved in proteolytic activation of Rim101p in response to alkaline pH; has similarity to A. nidulans palB
YNL294C	*RIM21*	-2.10	Component of the RIM101 pathway, has a role in cell wall construction and alkaline pH response; has similarity to A. nidulans PalH
YCR033W	*SNT1*	-2.08	Subunit of the Set3C deacetylase complex that interacts directly with the Set3C subunit, Sif2p; putative DNA-binding protein
YGL212W	*VAM7*	-2.08	Component of the vacuole SNARE complex involved in vacuolar morphogenesis; SNAP-25 homolog; functions with a syntaxin homolog Vam3p in vacuolar protein trafficking

Systematic Name	Common Name	Log_2 Fitness	Gene Description
YDR456W	*NHX1*	-2.08	Endosomal Na+/H+ exchanger, required for intracellular sequestration of Na+; required for osmotolerance to acute hypertonic shock
YKR029C	*SET3*	-2.07	Defining member of the SET3 histone deacetylase complex which is a meiosis-specific repressor of sporulation genes; necessary for efficient transcription by RNAPII; one of two yeast proteins that contains both SET and PHD domains
YLR191W	*PEX13*	-2.06	Integral peroxisomal membrane receptor for the PTS1 peroxisomal matrix protein signal recognition factor Pex5p, required for the translocation of peroxisomal matrix proteins, also interacts with Pex7p and Pex14p, contains a C-terminal SH3 domain
YCR079W		-2.05	Phosphoprotein phosphatase type 2C similar to mammalian PP1Ks; involved in mitophagy; localized to mitochondrial inner membrane space; null mutant is sensitive to rapamycin
YAL024C	*LTE1*	-2.05	Putative GDP/GTP exchange factor required for mitotic exit at low temperatures; acts as a guanine nucleotide exchange factor (GEF) for Tem1p, which is a key regulator of mitotic exit; physically associates with Ras2p-GTP
YPL051W	*ARL3*	-2.04	GTPase of the Ras superfamily required to recruit Arl1p to the Golgi; similar to ADP-ribosylation factor
YLR176C	*RFX1*	-2.03	Protein involved in DNA damage and replication checkpoint pathway; recruits repressors Tup1p and Cyc8p to promoters of DNA damage-inducible genes; similar to a family of mammalian DNA binding RFX1-4 proteins
YLR085C	*ARP6*	-2.02	Actin-related protein that binds nucleosomes; a component of the SWR1 complex, which exchanges histone variant H2AZ (Htz1p) for chromatin-bound histone H2A
YGR077C	*PEX8*	-2.02	Intraperoxisomal organizer of the peroxisomal import machinery, tightly associated with the lumenal face of the peroxisomal membrane, essential for peroxisome biogenesis, binds PTS1-signal receptor Pex5p
YDL226C	*GCS1*	-2.00	ADP-ribosylation factor GTPase activating protein (ARF GAP), involved in ER-Golgi transport; shares functional similarity with Glo3p
YHR200W	*RPN10*	-1.99	Non-ATPase base subunit of the 19S regulatory particle (RP) of the 26S proteasome; N-terminus plays a role in maintaining the structural integrity of the RP; binds selectively to polyubiquitin chains; homolog of the mammalian S5a protein
YJL204C	*RCY1*	-1.95	F-box protein involved in recycling plasma membrane proteins internalized by endocytosis; localized to sites of polarized growth
YIL065C	*FIS1*	-1.94	Mitochondrial outer membrane protein involved in membrane fission, required for localization of Dnm1p and Mdv1p during mitochondrial division
YDR349C	*YPS7*	-1.93	Putative GPI-anchored aspartic protease, located in the cytoplasm and endoplasmic reticulum
YJL024C	*APS3*	-1.84	Small subunit of the clathrin-associated adaptor complex AP-3, which is involved in vacuolar protein sorting; related to the sigma subunit of the mammalian clathrin AP-3 complex; suppressor of loss of casein kinase 1 function
YPR173C	*VPS4*	-1.81	AAA-type ATPase required for efficient late endosome to vacuole transport; catalyzes the release of an endosomal membrane-associated class E VPS protein complex; regulates cellular sterol metabolism; cytoplasmic and endosomal localization
YMR258C		-1.81	Protein of unknown function with similarity to F-box proteins; physically interacts with Skp1p; green fluorescent protein (GFP)-fusion protein localizes to the cytoplasm and nucleus; YMR258C is not an essential gene

Systematic Name	Common Name	Log$_2$ Fitness	Gene Description
YDR484W	*VPS52*	-1.80	Component of the GARP (Golgi-associated retrograde protein) complex, Vps51p-Vps52p-Vps53p-Vps54p, which is required for the recycling of proteins from endosomes to the late Golgi; involved in localization of actin and chitin
YMR183C	*SSO2*	-1.80	Plasma membrane t-SNARE involved in fusion of secretory vesicles at the plasma membrane; syntaxin homolog that is functionally redundant with Sso1p
YOR123C	*LEO1*	-1.78	Component of the Paf1 complex, which associates with RNA polymerase II and is involved in histone methylation
YMR077C	*VPS20*	-1.75	Myristoylated subunit of ESCRTIII, the endosomal sorting complex required for transport of transmembrane proteins into the multivesicular body pathway to the lysosomal/vacuolar lumen; cytoplasmic protein recruited to endosomal membranes
YDR049W		-1.75	Zinc finger protein; putative transcription factor that may interact with proteins involved in histone acetylation or deacetylation; may be involved in altering acetylation on histone lysines
YOR030W	*DFG16*	-1.72	Probable multiple transmembrane protein, involved in diploid invasive and pseudohyphal growth upon nitrogen starvation; required for accumulation of processed Rim101p
YLR027C	*AAT2*	-1.72	Cytosolic aspartate aminotransferase, involved in nitrogen metabolism; localizes to peroxisomes in oleate-grown cells
YOR323C	*PRO2*	-1.72	Gamma-glutamyl phosphate reductase, catalyzes the second step in proline biosynthesis
YGR133W	*PEX4*	-1.71	Peroxisomal ubiquitin conjugating enzyme required for peroxisomal matrix protein import and peroxisome biogenesis
YJL155C	*FBP26*	-1.69	Fructose-2,6-bisphosphatase, required for glucose metabolism
YDR270W	*CCC2*	-1.65	Cu(+2)-transporting P-type ATPase, required for export of copper from the cytosol into an extracytosolic compartment; has similarity to human proteins involved in Menkes and Wilsons diseases
YJL027C		-1.64	Putative protein of unknown function
YMR214W	*SCJ1*	-1.63	One of several homologs of bacterial chaperone DnaJ, located in the ER lumen where it cooperates with Kar2p to mediate maturation of proteins
YDL100C	*GET3*	-1.62	ATPase, subunit of the GET complex; required for the retrieval of HDEL proteins from the Golgi to the ER in an ERD2 dependent fashion; involved in resistance to heat and metal stress
YML041C	*VPS71*	-1.62	Nucleosome-binding component of the SWR1 complex, which exchanges histone variant H2AZ (Htz1p) for chromatin-bound histone H2A; required for vacuolar protein sorting
YDR295C	*HDA2*	-1.60	Subunit of a possibly tetrameric trichostatin A-sensitive class II histone deacetylase complex containing an Hda1p homodimer and an Hda2p-Hda3p heterodimer; involved in telomere maintenance
YBR288C	*APM3*	-1.58	Mu3-like subunit of the clathrin associated protein complex (AP-3); functions in transport of alkaline phosphatase to the vacuole via the alternate pathway
YMR216C	*SKY1*	-1.57	SR protein kinase (SRPK) involved in regulating proteins involved in mRNA metabolism and cation homeostasis; similar to human SRPK1
YDR276C	*PMP3*	-1.56	Small plasma membrane protein related to a family of plant polypeptides that are overexpressed under high salt concentration or low temperature, not essential for viability, deletion causes hyperpolarization of the plasma membrane potential
YGL066W	*SGF73*	-1.55	73 kDa subunit of SAGA histone acetyltransferase complex; involved in formation of the preinitiation complex assembly at promoters

Systematic Name	Common Name	Log_2 Fitness	Gene Description
YGL007W		-1.54	Dubious ORF located in the upstream region of PMA1, deletion leads to polyamine resistance due to downregulation of PMA1
YBR227C	*MCX1*	-1.54	Mitochondrial ATP-binding protein, possibly a mitochondrial chaperone with non-proteolytic function; similar to bacterial ClpX proteins
YGR270W	*YTA7*	-1.52	Protein of unknown function, member of CDC48/PAS1/SEC18 family of ATPases, potentially phosphorylated by Cdc28p
YIL005W	*EPS1*	-1.51	Pdi1p (protein disulfide isomerase)-related protein involved in endoplasmic reticulum retention of resident ER proteins
YIL077C		-1.51	Putative protein of unknown function; the authentic, non-tagged protein is detected in highly purified mitochondria in high-throughput studies; deletion confers sensitivity to 4-(N-(S-glutathionylacetyl)amino) phenylarsenoxide (GSAO)
YNR051C	*BRE5*	-1.50	Ubiquitin protease cofactor, forms deubiquitination complex with Ubp3p that coregulates anterograde and retrograde transport between the endoplasmic reticulum and Golgi compartments; null is sensitive to brefeldin A
YHR031C	*RRM3*	-1.49	DNA helicase involved in rDNA replication and Ty1 transposition; relieves replication fork pauses at telomeric regions; structurally and functionally related to Pif1p
YER151C	*UBP3*	-1.46	Ubiquitin-specific protease that interacts with Bre5p to co-regulate anterograde and retrograde transport between endoplasmic reticulum and Golgi compartments; inhibitor of gene silencing; cleaves ubiquitin fusions but not polyubiquitin
YNL097C	*PHO23*	-1.46	Probable component of the Rpd3 histone deacetylase complex, involved in transcriptional regulation of PHO5; C-terminus has similarity to human candidate tumor suppressor p33(ING1)
YPR179C	*HDA3*	-1.45	Subunit of a possibly tetrameric trichostatin A-sensitive class II histone deacetylase complex that contains an Hda1p homodimer and an Hda2p-Hda3p heterodimer; required for the activity of the complex; has similarity to Hda2p
YDR244W	*PEX5*	-1.45	Peroxisomal membrane signal receptor for C-terminal tripeptide signal sequence (PTS1) of peroxisomal matrix proteins, required for peroxisomal matrix protein import, tetratricopeptide repeat protein, also involved in PTS1-independent import
YIL153W	*RRD1*	-1.45	Activator of the phosphotyrosyl phosphatase activity of PP2A; regulates G1 phase progression, the G2/M phase transition, microtubule dynamics, the osmoresponse, bud morphogenesis and DNA repair; subunit of the Tap42p-Sit4p-Rrd1p complex
YPL055C	*LGE1*	-1.44	Protein of unknown function; null mutant forms abnormally large cells
YDR098C	*GRX3*	-1.43	Hydroperoxide and superoxide-radical responsive glutathione-dependent oxidoreductase; monothiol glutaredoxin subfamily member along with Grx4p and Grx5p; protects cells from oxidative damage
YOL044W	*PEX15*	-1.43	Phosphorylated tail-anchored type II integral peroxisomal membrane protein required for peroxisome biogenesis, cells lacking Pex15p mislocalize peroxisomal matrix proteins to cytosol, overexpression results in impaired peroxisome assembly
YKL216W	*URA1*	-1.39	Dihydroorotate dehydrogenase, catalyzes the fourth enzymatic step in the de novo biosynthesis of pyrimidines, converting dihydroorotic acid into orotic acid
YER084W		-1.39	Hypothetical protein

Systematic Name	Common Name	Log_2 Fitness	Gene Description
YKR042W	*UTH1*	-1.38	Mitochondrial outer membrane and cell wall localized SUN family member required for mitochondrial autophagy; involved in the oxidative stress response, life span during starvation, mitochondrial biogenesis, and cell death
YIL041W	*GVP36*	-1.37	Golgi vesicle protein of unknown function; localizes to both early and late Golgi vesicles; may interact with ribosomes, based on co-purification experiments
YGR182C		-1.36	Dubious open reading frame unlikely to encode a protein, based on available experimental and comparative sequence data; partially overlaps the verified ORF TIM13/YGR181W
YBR290W	*BSD2*	-1.36	Heavy metal ion homeostasis protein, facilitates trafficking of Smf1p and Smf2p metal transporters to the vacuole where they are degraded, controls metal ion transport, prevents metal hyperaccumulation, functions in copper detoxification
YNL323W	*LEM3*	-1.35	Membrane protein of the plasma membrane and ER, involved in translocation of phospholipids and alkylphosphocholine drugs across the plasma membrane
YNL329C	*PEX6*	-1.33	Peroxisomal membrane AAA-family ATPase peroxin required for peroxisome assembly, contains two 230 amino acid ATP-binding AAA cassettes, interacts with Pex1p
YHR206W	*SKN7*	-1.31	Nuclear response regulator and transcription factor, part of a branched two-component signaling system; required for optimal induction of heat-shock genes in response to oxidative stress; involved in osmoregulation
YEL037C	*RAD23*	-1.30	Protein with ubiquitin-like N terminus, recognizes and binds damaged DNA (with Rad4p) during nucleotide excision repair; regulates Rad4p levels, subunit of Nuclear Excision Repair Factor 2 (NEF2); homolog of human HR23A and HR23B proteins
YJL164C	*TPK1*	-1.28	Subunit of cytoplasmic cAMP-dependent protein kinase, which contains redundant catalytic subunits Tpk1p, Tpk2p, and Tpk3p and regulatory subunit Bcy1p; promotes vegetative growth in response to nutrients; inhibits filamentous growth
YJL130C	*URA2*	-1.28	Bifunctional carbamoylphosphate synthetase (CPSase)-aspartate transcarbamylase (ATCase), catalyzes the first two enzymatic steps in the de novo biosynthesis of pyrimidines; both activities are subject to feedback inhibition by UTP
YBR105C	*VID24*	-1.26	Peripheral membrane protein located at Vid (vacuole import and degradation) vesicles; regulates fructose-1,6-bisphosphatase (FBPase) targeting to the vacuole; involved in proteasome-dependent catabolite degradation of FBPase
YLR056W	*ERG3*	-1.21	C-5 sterol desaturase, catalyzes the introduction of a C-5(6) double bond into episterol, a precursor in ergosterol biosynthesis; mutants are viable, but cannot grow on non-fermentable carbon sources
YDR254W	*CHL4*	-1.19	Outer kinetochore protein required for chromosome stability, interacts with kinetochore proteins Ctf19p, Ctf3p, and Iml3p; exhibits a two-hybrid interaction with Mif2p; association with CEN DNA requires Ctf19p
YOR297C	*TIM18*	-1.16	Component of the mitochondrial Tim54p-Tim22p complex involved in insertion of polytopic proteins into the inner membrane; may function to stabilize the complex
YMR099C		-1.14	Glucose-6-phosphate 1-epimerase (hexose-6-phosphate mutarotase), likely involved in carbohydrate metabolism; GFP-fusion protein localizes to both the nucleus and cytoplasm and is induced in response to the DNA-damaging agent MMS

Systematic Name	Common Name	Log_2 Fitness	Gene Description
YDR297W	*SUR2*	-1.14	Sphinganine C4-hydroxylase, catalyses the conversion of sphinganine to phytosphingosine in sphingolipid biosyntheis
YHR204W	*MNL1*	-1.12	Alpha mannosidase-like protein of the endoplasmic reticulum required for degradation of glycoproteins but not for processing of N-linked oligosaccharides
YDR495C	*VPS3*	-1.07	Cytoplasmic protein required for the sorting and processing of soluble vacuolar proteins, acidification of the vacuolar lumen, and assembly of the vacuolar H+-ATPase
YPL138C	*SPP1*	-1.06	Subunit of COMPASS (Set1C), a complex which methylates histone H3 on lysine 4 and is required in telomeric transcriptional silencing; PHD finger domain protein similar to human CGBP, an unmethylated CpG binding protein
YOR106W	*VAM3*	-1.06	Syntaxin-related protein required for vacuolar assembly; functions with Vam7p in vacuolar protein trafficking; member of the syntaxin family of proteins
YLR015W	*BRE2*	-1.03	Subunit of the COMPASS (Set1C) complex, which methylates histone H3 on lysine 4 and is required in transcriptional silencing near telomeres; involved in telomere maintenance; similar to trithorax-group protein ASH2L
YIR034C	*LYS1*	-1.01	Saccharopine dehydrogenase (NAD+, L-lysine-forming), catalyzes the conversion of saccharopine to L-lysine, which is the final step in the lysine biosynthesis pathway
YOR043W	*WHI2*	-1.01	Protein required, with binding partner Psr1p, for full activation of the general stress response, possibly through Msn2p dephosphorylation; regulates growth during the diauxic shift; negative regulator of G1 cyclin expression
YCR007C		-0.98	Putative integral membrane protein, member of DUP240 gene family; YCR007C is not an essential gene
YDR395W	*SXM1*	-0.88	Nuclear transport factor (karyopherin) involved in protein transport between the cytoplasm and nucleoplasm; similar to Nmd5p, Cse1p, Lph2p, and the human cellular apoptosis susceptibility protein, CAS1
YKR077W		-0.85	Putative transcriptional activator, identified by high-throughput two-hybrid experiments; green fluorescent protein (GFP)-fusion protein localizes to the cytoplasm and nucleus; YKR077W is not an essential gene
YJL145W	*SFH5*	-0.80	Putative phosphatidylinositol transfer protein (PITP), exhibits phosphatidylinositol- but not phosphatidylcholine-transfer activity, mainly localized to cytosol and microsomes, similar to Sec14p; may be PITP regulator rather than actual PITP
YIL101C	*XBP1*	0.84	Transcriptional repressor that binds to promoter sequences of the cyclin genes, CYS3, and SMF2; expression is induced by stress or starvation during mitosis, and late in meiosis; member of the Swi4p/Mbp1p family; potential Cdc28p substrate
YPL241C	*CIN2*	0.95	Tubulin folding factor C (putative) involved in beta-tubulin (Tub2p) folding; isolated as mutant with increased chromosome loss and sensitivity to benomyl
YNR032C-A	*YNR032C-A*	0.99	Ubiquitin-like protein modifier, may function in modification of Sph1p and Hbt1p, functionally complemented by the human or S. pombe ortholog; mechanism of Hub1p adduct formation not yet clear
YLR344W	*RPL26A*	1.01	Protein component of the large (60S) ribosomal subunit, nearly identical to Rpl26Bp and has similarity to E. coli L24 and rat L26 ribosomal proteins; binds to 5.8S rRNA

Systematic Name	Common Name	Log_2 Fitness	Gene Description
YLR221C	*RSA3*	1.01	Protein with a likely role in ribosomal maturation, required for accumulation of wild-type levels of large (60S) ribosomal subunits; binds to the helicase Dbp6p in pre-60S ribosomal particles in the nucleolus
YLR304C	*ACO1*	1.03	Aconitase, required for the tricarboxylic acid (TCA) cycle and also independently required for mitochondrial genome maintenance; component of the mitochondrial nucleoid; mutation leads to glutamate auxotrophy
YLR315W	*NKP2*	1.07	Non-essential kinetochore protein, subunit of the Ctf19 central kinetochore complex (Ctf19p-Mcm21p-Okp1p-Mcm22p-Mcm16p-Ctf3p-Chl4p-Mcm19p-Nkp1p-Nkp2p-Ame1p-Mtw1p)
YDR156W	*RPA14*	1.14	RNA polymerase I subunit A14
YOL039W	*RPP2A*	1.18	Ribosomal protein P2 alpha, a component of the ribosomal stalk, which is involved in the interaction between translational elongation factors and the ribosome; regulates the accumulation of P1 (Rpp1Ap and Rpp1Bp) in the cytoplasm
YDR237W	*MRPL7*	1.35	Mitochondrial ribosomal protein of the large subunit
YJR050W	*ISY1*	1.37	Component of the spliceosome complex involved in pre-mRNA splicing, auxiliary splicing factor that may modulate Syf1p activity and help optimize splicing; isy1 syf2 double mutation activates the spindle checkpoint, causing cell cycle arrest
YKR092C	*SRP40*	1.46	Nucleolar, serine-rich protein with a role in preribosome assembly or transport; may function as a chaperone of small nucleolar ribonucleoprotein particles (snoRNPs); immunologically and structurally to rat Nopp140
YDR363W-A	*SEM1*	1.58	Component of the lid subcomplex of the regulatory subunit of the 26S proteasome; ortholog of human DSS1
YLR368W	*MDM30*	1.60	F-box protein; physically associates with mitochondria and is required for normal mitochondrial fusion in rich medium, during sporulation, and in mating cells; promotes ubiquitin-mediated degradation of Gal4p in some strains
YBL038W	*MRPL16*	1.65	Mitochondrial ribosomal protein of the large subunit
YGL072C		1.83	Dubious open reading frame unlikely to encode a protein; partially overlaps the verified gene HSF1
YDR121W	*DPB4*	2.17	Shared subunit of DNA polymerase epsilon and of ISW2/yCHRAC chromatin accessibility complex; involved in both chromosomal DNA replication and in inheritance of telomeric silencing
YGL136C	*MRM2*	2.18	Mitochondrial 21S rRNA methyltransferase, required for methylation of U(2791) in 21S rRNA; MRM2 deletion confers thermosensitive respiration and loss of mitochondrial DNA; has similarity to Spb1p and Trm7p, and to E. coli FtsJ/RrmJ
YFR036W	*CDC26*	2.31	Subunit of the Anaphase-Promoting Complex/Cyclosome (APC/C), which is a ubiquitin-protein ligase required for degradation of anaphase inhibitors, including mitotic cyclins, during the metaphase/anaphase transition
YDR457W	*TOM1*	3.41	E3 ubiquitin ligase of the hect-domain class; has a role in mRNA export from the nucleus and may regulate transcriptional coactivators
