# Supplementary material for: Novel insights into iron metabolism by integrating deletome and transcriptome analysis in an iron deficiency model of the yeast Saccharomyces cerevisiae
Source: BMC Genomics. 2009 Mar 25;10:130. doi: 10.1186/1471-2164-10-130 (PMC2669097; doi:10.1186/1471-2164-10-130)
Supplement: Additional file 2 — List of all genes that were identified by functional profiling in at least two out of three independent experiments. Deletion of these genes resulted in significant growth alterations in the presence of BPS compared to YPD media. [file 1471-2164-10-130-S2.pdf]

Additional File 2: Genes identified by functional profiling in at least two out of three independent replicate experiments. Deletion of these genes affects the fitness of mutant strains in iron deficiency. The requirement for growth of each gene was quantified as a $\log_2$ ratio of growth of the corresponding mutant strain in YPD with BPS compared to YPD alone. The $\log_2$ fitness represents the average of the significant values in these experiments. A negative value indicates sensitivity to BPS treatment while a positive one indicates resistance to BPS treatment.

.

ORF	Gene	$\log_2$ fitness
YDR269C	*CCC2*	-4.8
YER145C	*FTR1*	-4.7
YNL259C	*ATX1*	-4.6
YPL170W	*DAP1*	-4.3
YDR455C	*NHX1*	-4.3
YMR058W	*FET3*	-3.9
YPL182C	*CTI6*	-3.7
YPL181W	*CTI6*	-3.7
YJR040W	*GEF1*	-3.6
YKR052C	*MRS4*	-3.5
YJR033C	*RAV1*	-3.5
YCL008C	*STP22*	-3.4
YPL065W	*VPS28*	-3.3
YHL020C	*OPI1*	-3.3
YMR054W	*STV1*	-3.2
YLR025W	*SNF7*	-3.2
YIR033W	*MGA2*	-3.1
YKR019C	*IRS4*	-3.0
YGL045W	*RIM8*	-3.0
YBR164C	*ARL1*	-2.9
YPL139C	*UME1*	-2.9
YHR045W		-2.7
YJL211C	*PEX2*	-2.7
YLR417W	*VPS36*	-2.7
YJR102C	*VPS25*	-2.7
YHL027W	*RIM101*	-2.6
YDR203W	*RAV2*	-2.6
YJL004C	*SYS1*	-2.6
YDR202C	*RAV2*	-2.6

ORF	Gene	$\log_2$ fitness
YDL118W	*YDL119c*	-2.6
YGL153W	*PEX14*	-2.6
YMR057C	*FET3*	-2.6
YDR271C	*CCC2*	-2.6
YDR265W	*PEX10*	-2.6
YJL094C	*KHA1*	-2.5
YDL065C	*PEX19*	-2.5
YGL045W	*RIM8*	-2.5
YGR122W		-2.5
YDL119C		-2.4
YBR035C	*PDX3*	-2.4
YML071C	*COG8*	-2.4
YLL043W	*FPS1*	-2.4
YMR063W	*RIM9*	-2.3
YPL002C	*SNF8*	-2.3
YDR329C	*PEX3*	-2.3
YOR089C	*VPS21*	-2.3
YOR275C	*RIM20*	-2.3
YGL148W	*ARO2*	-2.2
YPR024W	*YME1*	-2.2
YLR214W	*FRE1*	-2.2
YGL152C	*PEX14*	-2.2
YKL041W	*VPS24*	-2.2
YKL213C	*DOA1*	-2.2
YNR006W	*VPS27*	-2.2
YMR202W	*ERG2*	-2.1
YKL197C	*PEX1*	-2.1
YCR068W	*ATG15*	-2.1
YMR154C	*RIM13*	-2.1

ORF	Gene	Log_2 fitness
YNL294C	*RIM21*	-2.1
YCR033W	*SNT1*	-2.1
YGL212W	*VAM7*	-2.1
YDR456W	*NHX1*	-2.1
YNL041C	*COG6*	-2.1
YKR029C	*SET3*	-2.1
YLR191W	*PEX13*	-2.1
YCR079W		-2.1
YAL024C	*LTE1*	-2.0
YPL051W	*ARL3*	-2.0
YGR077C	*PEX8*	-2.0
YOL081W	*IRA2*	-2.0
YLR176C	*RFX1*	-2.0
YLR085C	*ARP6*	-2.0
YDL226C	*GCS1*	-2.0
YHR200W	*RPN10*	-2.0
YBR058C	*UBP14*	-2.0
YLR119W	*SRN2*	-2.0
YJL204C	*RCY1*	-2.0
YIL065C	*FIS1*	-1.9
YDR048C		-1.9
YDR349C	*YPS7*	-1.9
YJL095W	*BCK1*	-1.9
YJL024C	*APS3*	-1.9
YGL005C	*COG7*	-1.8
YPR173C	*VPS4*	-1.8
YMR258C		-1.8
YDR484W	*VPS52*	-1.8
YDR334W	*SWR1*	-1.8
YMR183C	*SSO2*	-1.8
YOR123C	*LEO1*	-1.8
YLR027C	*AAT2*	-1.7
YMR077C	*VPS20*	-1.7
YDR049W		-1.7
YOR030W	*DFG16*	-1.7
YBR231C	*SWC5*	-1.7
YOR323C	*PRO2*	-1.7
YDR335W	*MSN5*	-1.7
YPL120W	*VPS30*	-1.7
YGR133W	*PEX4*	-1.7
YJL155C	*FBP26*	-1.7
YDR270W	*CCC2*	-1.7
YJL027C		-1.6
YMR214W	*SCJ1*	-1.6

ORF	Gene	Log_2 fitness
YDR389W	*SAC7*	-1.6
YDL100C	*ARR4*	-1.6
YML041C	*VPS71*	-1.6
YDR295C	*HDA2*	-1.6
YGR184C	*UBR1*	-1.6
YBR288C	*APM3*	-1.6
YLR111W		-1.6
YMR216C	*SKY1*	-1.6
YER040W	*GLN3*	-1.6
YGL066W	*SGF73*	-1.6
YDR276C	*PMP3*	-1.6
YGL007W		-1.5
YBR227C	*MCX1*	-1.5
YJL179W	*PFD1*	-1.5
YGR270W	*YTA7*	-1.5
YIL005W	*EPS1*	-1.5
YNR051C	*BRE5*	-1.5
YNR005C		-1.5
YIL077C		-1.5
YGR206W		-1.5
YNL084C	*END3*	-1.5
YHR031C	*RRM3*	-1.5
YFR010W	*UBP6*	-1.5
YHR179W	*OYE2*	-1.5
YER151C	*UBP3*	-1.5
YNL097C	*PHO23*	-1.5
YPR179C	*HDA3*	-1.5
YIL153W	*RRD1*	-1.5
YDR244W	*PEX5*	-1.5
YDR098C	*GRX3*	-1.4
YPL055C	*LGE1*	-1.4
YPL195W	*APL5*	-1.4
YOL044W	*PEX15*	-1.4
YIL041W		-1.4
YER084W		-1.4
YKL216W	*URA1*	-1.4
YMR263W	*SAP30*	-1.4
YLR110C	*CCW12*	-1.4
YPL154C	*PEP4*	-1.4
YKR042W	*UTH1*	-1.4
YNL323W	*LEM3*	-1.4
YGR182C		-1.4
YBR290W	*BSD2*	-1.4
YNL329C	*PEX6*	-1.3

ORF	Gene	Log_2 fitness
YHR206W	*SKN7*	-1.3
YEL037C	*RAD23*	-1.3
YJL164C	*TPK1*	-1.3
YBR103W	*SIF2*	-1.3
YJL130C	*URA2*	-1.3
YER073W	*ALD5*	-1.3
YDR162C	*NBP2*	-1.3
YBR105C	*VID24*	-1.3
YJL209W	*CBP1*	-1.3
YIR009W	*MSL1*	-1.3
YGL012W	*ERG4*	-1.3
YDR149C	*NUM1*	-1.2
YFL049W		-1.2
YCR050C		-1.2
YJR043C	*POL32*	-1.2
YHR004C	*NEM1*	-1.2
YLR087C	*CSF1*	-1.2
YLR056W	*ERG3*	-1.2
YLR204W	*QRI5*	-1.2
YBR174C		-1.2
YDL077C	*VAM6*	-1.2
YER019W	*ISC1*	-1.2
YOR297C	*TIM18*	-1.2
YDR254W	*CHL4*	-1.2
YGR092W	*DBF2*	-1.2
YGL194C	*HOS2*	-1.2
YOR002W	*ALG6*	-1.2
YOL096C	*COQ3*	-1.2
YMR219W	*ESC1*	-1.2
YPL265W	*DIP5*	-1.2
YCR065W	*HCM1*	-1.2
YJR120W		-1.2
YCR087W		-1.2
YDL052C	*SLC1*	-1.2
YGR252W	*GCN5*	-1.2
YPR070W	*MED1*	-1.2
YLR238W	*FAR10*	-1.2
YER093C	*TSC11*	-1.1
YGL054C	*ERV14*	-1.1
YNL016W	*PUB1*	-1.1
YEL031W	*SPF1*	-1.1
YMR099C		-1.1
YKL126W	*YPK1*	-1.1
YDR297W	*SUR2*	-1.1

ORF	Gene	Log_2 fitness
YDR393W	*SHE9*	-1.1
YJL166W	*QCR8*	-1.1
YGR181W	*TIM13*	-1.1
YHR204W	*MNL1*	-1.1
YLR360W	*VPS38*	-1.1
YJL185C		-1.1
YDR469W	*SDC1*	-1.1
YLR330W	*CHS5*	-1.1
YIL097W	*FYV10*	-1.1
YDL074C	*BRE1*	-1.1
YOL004W	*SIN3*	-1.1
YPR124W	*CTR1*	-1.1
YDR495C	*VPS3*	-1.1
YCR087C-A		-1.1
YMR052C-A		-1.1
YFL025C	*BST1*	-1.1
YOR106W	*VAM3*	-1.1
YKL046C	*DCW1*	-1.1
YLR268W	*SEC22*	-1.1
YKR094C	*RPL40B*	-1.1
YOR360C	*PDE2*	-1.1
YPL138C	*SPP1*	-1.1
YOR069W	*VPS5*	-1.1
YCL064C	*CHA1*	-1.0
YDL041W		-1.0
YGL025C	*PGD1*	-1.0
YLR015W	*BRE2*	-1.0
YOR140W	*SFL1*	-1.0
YPL178W	*CBC2*	-1.0
YBR291C	*CTP1*	-1.0
YMR070W	*MOT3*	-1.0
YML097C	*VPS9*	-1.0
YPL270W	*MDL2*	-1.0
YBR195C	*MSI1*	-1.0
YJL149W		-1.0
YOL012C	*HTZ1*	-1.0
YOL018C	*TLG2*	-1.0
YIR034C	*LYS1*	-1.0
YOR043W	*WHI2*	-1.0
YGR132C	*PHB1*	-1.0
YCL010C	*SGF29*	-1.0
YLR420W	*URA4*	-1.0
YPR052C	*NHP6A*	-1.0
YHR077C	*NMD2*	-1.0

ORF	Gene	Log_2 fitness
YER007C-A		-1.0
YDR435C	*PPM1*	-1.0
YDL076C	*RXT3*	-1.0
YIL029C		-1.0
YBL039C	*URA7*	-1.0
YDR485C	*VPS72*	-1.0
YGL250W		-1.0
YCR007C		-1.0
YNL001W	*DOM34*	-1.0
YDR314C		-1.0
YPR089W		-1.0
YPL144W		-1.0
YCR045C		-0.9
YDR120C	*TRM1*	-0.9
YMR311C	*GLC8*	-0.9
YHR012W	*VPS29*	-0.9
YGR171C	*MSM1*	-0.9
YOR139C		-0.9
YER087C-A		-0.9
YLR292C	*SEC72*	-0.9
YIL038C	*NOT3*	-0.9
YJL124C	*LSM1*	-0.9
YJL154C	*VPS35*	-0.9
YER031C	*YPT31*	-0.9
YOR039W	*CKB2*	-0.9
YEL064C	*AVT2*	-0.9
YKL220C	*FRE2*	-0.9
YOR068C	*VAM10*	-0.9
YOR191W	*RIS1*	-0.9
YDL229W	*SSB1*	-0.9
YJL206C		-0.9
YOR366W		-0.9
YNL206C	*RTT106*	-0.9
YDR395W	*SXM1*	-0.9
YNL107W	*YAF9*	-0.9
YKL053C-A	*MDM35*	-0.9
YNL299W	*TRF5*	-0.9
YKR077W		-0.9
YPL213W	*LEA1*	-0.9
YMR246W	*FAA4*	-0.9
YOR014W	*RTS1*	-0.9
YMR135C	*GID8*	-0.8
YJR060W	*CBF1*	-0.8
YGR261C	*APL6*	-0.8

ORF	Gene	Log_2 fitness
YDL240W	*LRG1*	-0.8
YNR032W	*PPG1*	-0.8
YOR025W	*HST3*	-0.8
YER032W	*FIR1*	-0.8
YBR106W	*PHO88*	-0.8
YGR135W	*PRE9*	-0.8
YPR083W	*MDM36*	-0.8
YLR133W	*CKI1*	-0.8
YDR005C	*MAF1*	-0.8
YLR213C	*CRR1*	-0.8
YOL115W	*TRF4*	-0.8
YLR451W	*LEU3*	-0.8
YJL145W	*SFH5*	-0.8
YOR085W	*OST3*	-0.8
YDR143C	*SAN1*	-0.8
YDR537C		-0.8
YGL253W	*HXK2*	-0.8
YDR363W	*ESC2*	-0.8
YOL020W	*TAT2*	-0.8
YIL002C	*INP51*	-0.8
YOR379C		-0.8
YOL064C	*MET22*	-0.8
YDL234C	*GYP7*	-0.8
YEL062W	*NPR2*	-0.8
YKR035W-A	*DID2*	-0.8
YDR075W	*PPH3*	-0.7
YDL167C	*NRP1*	-0.7
YMR291W		-0.7
YJR117W	*STE24*	-0.7
YER075C	*PTP3*	-0.7
YMR052W	*FAR3*	-0.7
YER019C-A	*SBH2*	-0.7
YMR123W	*PKR1*	-0.7
YJL217W		-0.7
YDR391C		-0.7
YHL037C		-0.7
YBR101C	*FES1*	-0.7
YLR036C		-0.7
YBL051C	*PIN4*	-0.7
YHL042W		-0.7
YBR089C-A	*NHP6B*	-0.7
YLR126C		-0.7
YBR208C	*DUR1,2*	-0.7
YIL039W		-0.7

ORF	Gene	Log_2 fitness
YIL053W	*RHR2*	-0.6
YOR322C		-0.6
YIL016W	*SNL1*	-0.6
YDL091C	*UBX3*	-0.6
YEL056W	*HAT2*	-0.6
YHR153C	*SPO16*	-0.6
YHL014C	*YLF2*	-0.6
YLL001W	*DNM1*	-0.6
YHR017W	*YSC83*	-0.6
YOR345C		-0.6
YPL137C		-0.6
YEL060C	*PRB1*	-0.6
YOR008C-A		-0.6
YDR035W	*ARO3*	-0.6
YMR276W	*DSK2*	-0.6
YNL105W		-0.6
YDL023C	*GPD1 - overlap*	-0.6
YJL215C		-0.5
YOL027C	*MDM38*	-0.5
YMR274C	*RCE1*	-0.5
YHL040C	*ARN1*	-0.5
YHR104W	*GRE3*	-0.5
YNL104C	*LEU4*	-0.4
YOR005C	*DNL4*	-0.4
YOR059C		-0.4
YPL110C		-0.4
YLR131C	*ACE2*	-0.3

YDR457W	*TOM1*	3.4
YFR036W	*CDC26*	2.3
YDR121W	*DPB4*	2.2
YGL136C	*MRM2*	2.2
YGR220C	*MRPL9*	2.0
YGR102C		1.9
YGL072C		1.9
YPR166C	*MRP2*	1.8
YOR158W	*PET123*	1.7
YBL038W	*MRPL16*	1.7
YLR368W	*MDM30*	1.7
YDR363W-A	*SEM1*	1.6
YOR295W	*UAF30*	1.6
YBR278W	*DPB3*	1.6
YJL023C	*PET130*	1.6

ORF	Gene	Log_2 fitness
YER110C	*KAP123*	1.5
YKR092C	*SRP40*	1.5
YCR028C	*FEN2*	1.5
YER017C	*AFG3*	1.5
YJR050W	*ISY1*	1.4
YHR167W	*THP2*	1.4
YDR237W	*MRPL7*	1.4
YML094C-A		1.4
YGL064C	*MRH4*	1.4
YER139C		1.4
YBR078W	*ECM33*	1.3
YCR051W		1.3
YCR003W	*MRPL32*	1.3
YKL056C		1.3
YIR005W	*IST3*	1.3
YPR087W	*VPS69*	1.3
YJR139C	*HOM6*	1.2
YKL074C	*MUD2*	1.2
YOR141C	*ARP8*	1.2
YOR182C	*RPS30B*	1.2
YNL215W	*IES2*	1.2
YOL039W	*RPP2A*	1.2
YLL045C	*RPL8B*	1.2
YGR078C	*PAC10*	1.2
YGR192C	*TDH3*	1.2
YML122C		1.2
YNR037C	*RSM19*	1.2
YER002W	*NOP16*	1.2
YER153C	*PET122*	1.2
YHR134W	*WSS1*	1.2
YDR156W	*RPA14*	1.2
YOR150W	*MRPL23*	1.2
YLR450W	*HMG2*	1.1
YDR447C	*RPS17B*	1.1
YER092W	*IES5*	1.1
YFR032C-A	*RPL29*	1.1
YLR373C	*VID22*	1.1
YKL110C	*KTI12*	1.1
YDR101C	*ARX1*	1.1
YNL198C		1.1
YDL002C	*NHP10*	1.1
YGR233C	*PHO81*	1.1
YDL059C	*RAD59*	1.1
YER156C		1.1

ORF	Gene	Log_2 fitness
YLL044W		1.1
YNL025C	*SSN8*	1.1
YLR315W	*NKP2*	1.1
YLR032W	*RAD5*	1.1
YGL149W		1.1
YPR057W	*BRR1*	1.0
YOR349W	*CIN1*	1.0
YLR304C	*ACO1*	1.0
YKR048C	*NAP1*	1.0
YMR269W		1.0
YDR385W	*EFT2*	1.0
YOR235W		1.0
YLR221C	*RSA3*	1.0
YOR189W	*IES4*	1.0
YLR344W	*RPL26A*	1.0
YGL060W	*YBP2*	1.0
YGR034W	*RPL26B*	1.0
YNR032C-A	*HUB1*	1.0
YPL158C		1.0
YNL096C	*RPS7B*	1.0
YGR076C	*MRPL25*	1.0
YFR031C	*SMC2*	1.0
YPL241C	*CIN2*	1.0
YNL071W	*LAT1*	0.9
YKR072C	*SIS2*	0.9
YJR077C	*MIR1*	0.9
YDR179C	*CSN9*	0.9
YKL138C	*MRPL31*	0.9
YBR246W		0.9
YPL104W	*MSD1*	0.9
YIL103W	*DPH1*	0.9
YNR042W		0.9
YNL248C	*RPA49*	0.9
YFL013W-A		0.9
YNL056W		0.9
YOR230W	*WTM1*	0.9
YKL008C	*LAC1*	0.9
YDR442W	*SSN2**	0.9
YOR330C	*MIP1*	0.8
YMR207C	*HFA1*	0.8
YIL121W	*QDR2*	0.8
YIL101C	*XBP1*	0.8
YHR057C	*CPR2*	0.8
YOR027W	*STI1*	0.8

ORF	Gene	Log_2 fitness
YJR097W	*JJJ3*	0.8
YPL202C	*AFT2*	0.8
YGR035C		0.8
YKR074W		0.8
YLL006W	*MMM1*	0.8
YDR399W	*HPT1*	0.8
YBL027W	*RPL19B*	0.8
YPL239W	*YAR1*	0.7
YJL093C	*TOK1*	0.7
YDR414C	*ERD1*	0.7
YCR066W	*RAD18*	0.7
YIL112W	*HOS4*	0.7
YIL043C	*CBR1*	0.7
YBR277C		0.7
YDR403W	*DIT1*	0.7
YLR233C	*EST1*	0.7
YBL080C	*PET112*	0.7
YDR072C	*IPT1*	0.7
YGR118W	*RPS23A*	0.7
YKL087C	*CYT2*	0.7
YGR228W		0.7
YJL192C	*SOP4*	0.7
YDL106C	*PHO2*	0.6
YKR047W		0.6
YNL183C	*NPR1*	0.6
YHR094C	*HXT1*	0.6
YDR431W		0.6
YCR015C		0.6
YBR031W	*RPL4A*	0.6
YNL285W		0.6
YDR490C	*PKH1*	0.6
YOR327C	*SNC2*	0.6
YKL068W	*NUP100*	0.6
YOL079W		0.6
YML107C		0.6
YPL172C	*COX10*	0.5
YCR008W	*SAT4*	0.5
YDR493W		0.5
YIL069C	*RPS24B*	0.5
YOL070C		0.5
YNR067C	*DSE4*	0.4
YPL057C	*SUR1*	0.4
YHR108W	*GGA2*	0.4
YGR221C	*TOS2*	0.3
