# Supplementary material for: Novel insights into iron metabolism by integrating deletome and transcriptome analysis in an iron deficiency model of the yeast Saccharomyces cerevisiae
Source: BMC Genomics. 2009 Mar 25;10:130. doi: 10.1186/1471-2164-10-130 (PMC2669097; doi:10.1186/1471-2164-10-130)
Supplement: Additional file 3 — Comparison of yeast functional data in iron deficiency between this and other published studies. Genes identified in this study y functional profiling were compared to those reported by Davis-Kaplan et al. (2004), Dudley et al. (2005) and Lesuisse et al. (2005). [file 1471-2164-10-130-S3.pdf]

Additional File 3: Comparison of genes identified by functional profiling in different studies reported in the literature using yeast deletion mutants. Genes from Additional file 1 from this study were compared to those reported by Davis-Kaplan *et al.*[*J Biol Chem* 2004, **279**(6):4322-4329], Dudley *et al*. [*Mol Syst Biol* 2005, **1**(1):msb4100004-E4100001], and Lesuisse *et al*. [*Genetics* 2005, **169**(1):107-122]. Only the genes with the highest degree of confidence from these studies are compared in the table. Genes in grey boxes indicate an overlap of a gene identified in this study with at least one from the other studies.

**This study**	**Davis-Kaplan *et al.***	**Dudley *et al.***	**Lesuisse *et al.***
AAT2			
	AFT1	*AFT1*	*AFT1*
APM3			
APS3			*APS3*
			ARF1
ARL1			
ARL3			
ARO2			
			ARP5
ARP6			
ATX1			*ATX1*
	BEM2		
BRE2			
BRE5			
			BRO1
BSD2			
			BUD25
			BUD32
CCC2	*CCC2*		*CCC2*
			CCR4
			CDC50
			CDC73
CHL4			
COG8			
	CTR1		*CTR1*
	CUP5	*CUP5*	
	CWH36	*CWH36*	
			CYC8
			CYS4
DAP1			
			DEG1
DFG16			
			DIA2
DOA1			
		DOA4	
			EBS1
EPS1			
ERG2			
ERG3			
		ERG24	
FBP26			
FET3	*FET3*		
FIS1			
			FMC1
FPS1			
FRE1			
			FRE4
FTR1	*FTR1*		

**This study**	**Davis-Kaplan *et al.***	**Dudley *et al.***	**Lesuisse *et al.***
GCS1		*GCS1*	
GEF1	*GEF1*		
GET3			
			GGA2
			GGC1
GRX3			
			GRX5
GVP36			
HDA2			
HDA3			
			HEM14
			HSP12
		IES6	
			IMG2
IRS4			
KHA1			
		KRE22	
LEM3			
LEO1			
LGE1			
			LPD1
			LSM1
			LSM6
			LSM7
LTE1			
LYS1			
		MAC1	
MCX1			
			MED1
MGA2			
			MMR1
MNL1			
			MRS1
MRS4			
			MTM1
		NEM1	
		NGG1	
NHX1			
			NPL6
			PAT1
PDX3			
			PEP7
			PEP12
PEX1			
PEX3			
PEX4			
PEX5			
PEX6			
PEX8			
PEX10			
PEX13			
PEX14			
PEX15			
PEX19			
PHO23			
		PHO86	
	PKR1	*PKR1*	*PKR1*
PMP3			
			POR1
		PPA1	
			PPZ1
PRO2			
RAD23			
RAV1			*RAV1*
RAV2			
RCY1			*RCY1*
RFX1			
RIM8			
RIM9			
RIM13			
RIM20			
RIM21			
RIM101			
RPN10			
			RPS11A
RRD1			
RRM3			
			RSM19

This study	Davis-Kaplan *et al.*	Dudley *et al.*	Lesuisse *et al.*
SCJ1			
		SEC22	
SET3			
SFH5			
SGF73			
		SHP1	
			SIT1
SKN7			
SKY1			
SNF7			*SNF7*
SNF8			
SNT1			
SPP1			
			SPT4
			SRB5
			SRB8
			SSN3
			SSN8
SSO2			
			SSQ1
STP22			
STV1			
SUR2			
		SWI3	
		SWI4	
SXM1			
SYS1			
			TAF14
		TFP1	
		TFP3	
TIM18			
			TOM5
TPK1			
			TUP1
UBP3			
UME1			
		UMP1	
URA1			
URA2			

This study	Davis-Kaplan *et al.*	Dudley *et al.*	Lesuisse *et al.*
VAM3			
			VAM6
VAM7			
VID24			
			VID28
	VMA2	*VMA2*	
		VMA6	*VMA6*
	VMA8	*VMA8*	
			VMA10
		VMA13	*VMA13*
	VMA21	*VMA21*	*VMA21*
		VMA22	
	VPH2		
VPS3			
VPS4			*VPS4*
			VPS9
VPS20			*VPS20*
VPS21			
VPS24			
VPS25			*VPS25*
VPS27			
VPS28			*VPS28*
VPS36			*VPS36*
VPS52			
			VPS63
			VPS64
VPS71			
WHI2			
YME1			*YME1*
YPS7			
YTA7			
	ZAP1	*ZAP1*	
YCR007C			
			YCR024C
YCR079W			
YDL118W			
YDL119C			
YDR049W			
			YDR199W

This study	Davis-Kaplan *et al.*	Dudley *et al.*	Lesuisse *et al.*
YDR269C			
YDR271C			
YDR455C			
YER084W			
YGL007W			
YGL045W			
YGL152C			
			YGL220
			YGR064W
YGR122W			
YGR182C			
YHR045W			
			YHR100C
YIL077C			
YJL027C			
YJL211C			

This study	Davis-Kaplan *et al.*	Dudley *et al.*	Lesuisse *et al.*
	YKL118W	*YKL118W*	
YKR077W			
			YLL029W
			YLL030C
			YLR358C
		YLR386W	
		YML013C-A	
YMR057C			
YMR099C			
YMR258C			
		YNL080C	
		YOR139C	
		YOR331C	
YPL182C			
	YPR123C		
