# Supplementary material for: Novel insights into iron metabolism by integrating deletome and transcriptome analysis in an iron deficiency model of the yeast Saccharomyces cerevisiae
Source: BMC Genomics. 2009 Mar 25;10:130. doi: 10.1186/1471-2164-10-130 (PMC2669097; doi:10.1186/1471-2164-10-130)
Supplement: Additional file 4 — Gene Ontology enrichment analysis of functional profiling data. Gene Ontology molecular functions, biological processes and cellular components that were significantly enriched with genes identified by functional profiling. [file 1471-2164-10-130-S4.pdf]

**Additional File 4:** Gene Ontology enrichment analysis of genes inducing differential fitness in iron deficiency. Gene Ontology molecular functions, biological processes and cellular components that were significantly enriched with genes identified by functional profiling were identified using Funspec (http://funspec.med.utoronto.ca/). In the tables, *k* represents the number of identified genes and *f* the total number of genes in the category. Only the categories with hypergeometric P < 0.01 are shown.

| **GO Molecular Function** | | | | | |
|---|---|---|---|---|---|
| **Category** | **GO ID** | **p-value** | **In Category from Cluster** | **k** | **f** |
| Peroxisome targeting sequence binding | 0000268 | 1.91E-03 | *PEX5 PEX8* | 2 | 3 |

| **GO Biological Process** | | | | | |
|---|---|---|---|---|---|
| **Category** | **GO ID** | **p-value** | **In Category from Cluster** | **k** | **f** |
| Transport | 0006810 | 1.87E-14 | *VID24 ARL1 APM3 BSD2 STP22 PEX19 YDL119C GCS1 PEX5 CCC2 PMP3 PEX3 SXM1 NHX1 TOM1 SAC2 VPS3 FTR1 PEX14 VAM7 PEX8 EPS1 SYS1 APS3 KHA1 SFH5 RCY1 VPS25 VPS24 MRS4 SRP40 FPS1 SNF7 ARP6 PEX13 FRE1 VPS36 VPS71 COG8 FET3 VPS20 SSO2 SKY1 ATX1 VPS27 PEX15 VPS21 VAM3 TIM18 SNF8 ARL3 VPS28 VPS4* | 53 | 698 |
| Organelle organization and biogenesis | 0006996 | 2.44E-12 | *BSD2 STP22 PEX19 GCS1 RAV2 PEX5 PEX10 PEX3 NHX1 SAC2 VPS3 CDC26 PEX14 PEX8 PEX4 EPS1 FIS1 SYS1 RAV1 VPS25 VPS24 PEX1 UTH1 SNF7 ARP6 PEX13 MDM30 VPS36 VPS71 STV1 VPS20 PEX6 VPS27 PEX15 VPS21 TIM18 SNF8 VPS28 CIN2 YME1 VPS4* | 41 | 485 |
| Peroxisome organization and biogenesis | 0007031 | 6.98E-12 | *PEX19 PEX5 PEX10 PEX3 PEX14 PEX8 PEX4 PEX1 PEX13 PEX6 PEX15* | 11 | 25 |
| Intracellular protein transport | 0006886 | 3.11E-11 | *APM3 BSD2 STP22 PEX19 GCS1 PEX5 PEX3 SAC2 VPS3 PEX14 VAM7 PEX8 EPS1 SYS1 APS3 VPS25 ARP6 PEX13 VPS36 VPS71 COG8 SSO2 SKY1 VPS27 PEX15 VPS21 VAM3 TIM18 SNF8 ARL3 VPS28 VPS4* | 32 | 331 |
| Protein transport | 0015031 | 6.79E-10 | *APM3 BSD2 STP22 PEX19 GCS1 PEX5 PEX3 SAC2 VPS3 PEX14 VAM7 PEX8 EPS1 SYS1 APS3 RCY1 VPS25 ARP6 PEX13 VPS36 VPS71 COG8 SSO2 SKY1 VPS27 PEX15 VPS21 VAM3 TIM18 SNF8 ARL3 VPS28 VPS4* | 33 | 394 |
| Protein-peroxisome targeting | 0006625 | 4.41E-09 | *PEX19 PEX5 PEX3 PEX14 PEX8 PEX13 PEX15* | 7 | 12 |
| Protein targeting | 0006605 | 4.99E-09 | *BSD2 STP22 PEX19 PEX5 PEX3 VPS3 PEX14 PEX8 EPS1 VPS25 ARP6 PEX13 VPS36 VPS71 SKY1 VPS27 PEX15 VPS21 TIM18 SNF8 VPS28 VPS4* | 22 | 200 |

| Category | GO ID | p-value | In Category from Cluster | k | f |
|---|---|---|---|---|---|
| Cytoplasm organization and biogenesis | 0007028 | 1.82E-08 | *BSD2 STP22 PEX19 GCS1 RAV2 PEX5 PEX10 PEX3 NHX1 SAC2 VPS3 CDC26 PEX14 PEX8 PEX4 EPS1 FIS1 SYS1 RAV1 VPS25 VPS24 PEX1 UTH1 SNF7 ARP6 PEX13 MDM30 VPS36 VPS71 STV1 VPS20 PEX6 VPS27 PEX15 VPS21 TIM18 SNF8 VPS28 CIN2 YME1 VPS4* | 41 | 646 |
| Vacuole organization and biogenesis | 0007033 | 6.98E-07 | *BSD2 STP22 RAV2 NHX1 VPS3 RAV1 VPS25 ARP6 VPS36 VPS71 STV1 VPS21 SNF8 VPS28* | 14 | 111 |
| Protein-vacuolar targeting | 0006623 | 1.90E-06 | *BSD2 STP22 VPS3 VPS25 ARP6 VPS36 VPS71 VPS21 SNF8 VPS28* | 10 | 59 |
| Cell organization and biogenesis | 0016043 | 1.90E-06 | *BSD2 STP22 SNT1 PEX19 GCS1 RAV2 PEX5 PEX10 PEX3 NHX1 TOM1 SAC2 VPS3 CDC26 PEX14 PEX8 PEX4 EPS1 FIS1 SYS1 RAV1 VPS25 VPS24 PEX1 SET3 UTH1 BRE2 SNF7 ARP6 PEX13 MDM30 VPS36 VPS71 STV1 VPS20 PHO23 PEX6 VPS27 PEX15 VPS21 TIM18 SNF8 VPS28 SPP1 CIN2 YME1 VPS4* | 47 | 939 |
| Cell growth and/or maintenance | 0008151 | 6.35E-06 | *LTE1 MRPL16 PDX3 VID24 ARL1 APM3 BSD2 STP22 SNT1 PEX19 YDL119C GCS1 GRX3 DPB4 RPA14 RAV2 MRPL7 PEX5 CHL4 PEX10 CCC2 PMP3 SUR2 PEX3 SEM1 SXM1 NHX1 TOM1 SAC2 VPS3 RAD23 FTR1 UBP3 CDC26 MRM2 ARO2 PEX14 VAM7 PEX8 PEX4 YTA7 OPI1 RIM101 RRM3 RPN10 MNL1 SKN7 EPS1 FIS1 XBP1 RRD1 MGA2 LYS1 SYS1 APS3 KHA1 URA2 SFH5 FBP26 TPK1 RCY1 RAV1 GEF1 ISY1 VPS25 VPS24 PEX1 DOA1 URA1 SET3 UTH1 MRS4 SRP40 FPS1 BRE2 SNF7 AAT2 ERG3 ARP6 PEX13 FRE1 ACO1 RPL26A MDM30 VPS36 VPS71 COG8 STV1 FET3 RIM9 VPS20 RIM13 SSO2 ERG2 SCJ1 SKY1 PHO23 ATX1 RIM21 PEX6 VPS27 RPP2A PEX15 DFG16 WHI2 VPS21 VAM3 LEO1 RIM20 TIM18 PRO2 SNF8 ARL3 LGE1 VPS28 SPP1 UME1 CIN2 YME1 VPS4* | 120 | 3657 |
| Regulation of transcription by carbon catabolites | 0045990 | 1.63E-05 | *VPS25 VPS36 SNF8* | 3 | 3 |
| Regulation of transcription by glucose | 0046015 | 1.63E-05 | *VPS25 VPS36 SNF8* | 3 | 3 |
| Negative regulation of transcription by glucose | 0045014 | 1.63E-05 | *VPS25 VPS36 SNF8* | 3 | 3 |
| Negative regulation of transcription by carbon catabolites | 0045013 | 1.63E-05 | *VPS25 VPS36 SNF8* | 3 | 3 |
| Endosome transport | 0016197 | 2.32E-05 | *NHX1 SYS1 VPS24 SNF7 VPS20 VPS4* | 6 | 24 |
| Endosome organization and biogenesis | 0007032 | 2.32E-05 | *NHX1 SYS1 VPS24 SNF7 VPS20 VPS4* | 6 | 24 |

| Category | GO ID | p-value | In Category from Cluster | k | f |
|---|---|---|---|---|---|
| Protein metabolism | 0019538 | 3.30E-05 | *MRPL16 APM3 BSD2 STP22 SNT1 PEX19 GCS1 MRPL7 PEX5 PEX3 TOM1 SAC2 VPS3 UBP3 CDC26 PEX14 VAM7 PEX8 PEX4 YTA7 RPN10 MNL1 EPS1 SYS1 APS3 TPK1 VPS25 DOA1 SET3 BRE2 ARP6 PEX13 RPL26A VPS36 VPS71 COG8 RIM13 SSO2 SCJ1 SKY1 VPS27 RPP2A PEX15 VPS21 VAM3 RIM20 TIM18 SNF8 VPS28 SPP1 VPS4* | 51 | 1168 |
| Vesicle-mediated transport | 0016192 | 3.77E-05 | *VID24 ARL1 APM3 GCS1 NHX1 SAC2 VAM7 SYS1 APS3 RCY1 VPS24 SNF7 VPS20 SSO2 VPS21 VAM3 VPS4* | 17 | 219 |
| Golgi to vacuole transport | 0006896 | 6.67E-05 | *APM3 SAC2 VAM7 APS3 VAM3* | 5 | 18 |
| Post Golgi transport | 0006892 | 1.65E-04 | *APM3 SAC2 VAM7 SYS1 APS3 SSO2 VAM3* | 7 | 47 |
| Late endosome to vacuole transport | 0045324 | 2.44E-04 | *VPS24 SNF7 VPS20 VPS4* | 4 | 13 |
| Hydrogen ion homeostasis | 0030641 | 3.58E-04 | *RAV2 NHX1 RIM101 RAV1 STV1* | 5 | 25 |
| Regulation of pH | 0006885 | 3.58E-04 | *RAV2 NHX1 RIM101 RAV1 STV1* | 5 | 25 |
| Cation transport | 0006812 | 4.11E-04 | *BSD2 CCC2 PMP3 NHX1 KHA1 FRE1 FET3 ATX1* | 8 | 71 |
| Secretory pathway | 0045045 | 5.49E-04 | *APM3 GCS1 SAC2 VAM7 EPS1 SYS1 APS3 VPS36 COG8 SSO2 VPS27 VAM3 VPS4* | 13 | 177 |
| Heavy metal ion transport | 0006823 | 7.37E-04 | *BSD2 CCC2 FRE1 FET3 ATX1* | 5 | 29 |
| Protein secretion | 0009306 | 7.53E-04 | *APM3 GCS1 SAC2 VAM7 EPS1 SYS1 APS3 VPS36 COG8 SSO2 VPS27 VAM3 VPS4* | 13 | 183 |
| Monovalent inorganic cation homeostasis | 0030004 | 1.01E-03 | *RAV2 NHX1 RIM101 RAV1 STV1* | 5 | 31 |
| Vacuolar acidification | 0007035 | 1.18E-03 | *RAV2 NHX1 RAV1 STV1* | 4 | 19 |
| Protein-Golgi targeting | 0000042 | 1.72E-03 | *VPS36 VPS27 VPS4* | 3 | 10 |
| Golgi retention | 0045053 | 1.72E-03 | *VPS36 VPS27 VPS4* | 3 | 10 |
| Metal ion transport | 0030001 | 2.29E-03 | *BSD2 CCC2 FRE1 FET3 ATX1* | 5 | 37 |
| Golgi organization and biogenesis | 0007030 | 2.32E-03 | *VPS36 VPS27 VPS4* | 3 | 11 |
| Transition metal transport | 0000041 | 2.48E-03 | *CCC2 FRE1 FET3 ATX1* | 4 | 23 |
| Ion transport | 0006811 | 2.99E-03 | *BSD2 CCC2 PMP3 NHX1 KHA1 FRE1 FET3 ATX1* | 8 | 96 |
| Copper ion transport | 0006825 | 3.03E-03 | *CCC2 FRE1 ATX1* | 3 | 12 |
| Ion homeostasis | 0006873 | 5.06E-03 | *RAV2 NHX1 RIM101 RAV1 GEF1 STV1 SKY1* | 7 | 83 |
| Vacuolar transport | 0007034 | 8.43E-03 | *RAV2 NHX1 RAV1 STV1* | 4 | 32 |
| Negative regulation of meiosis | 0045835 | 9.08E-03 | *SNT1 SET3* | 2 | 6 |
| Homeostasis | 0019725 | 9.89E-03 | *RAV2 NHX1 RIM101 RAV1 GEF1 STV1 SKY1* | 7 | 94 |

| GO Cellular Component | | | | | |
|---|---|---|---|---|---|
| **Category** | **GO ID** | **p-value** | **In Category from Cluster** | **k** | **f** |
| Endosome | 0005768 | 5.24E-10 | *STP22 NHX1 VPS25 VPS24 SNF7 VPS36 VPS20 VPS27 VPS21 SNF8 VPS28* | 11 | 35 |
| Peroxisome | 0005777 | 5.11E-07 | *PEX5 PEX3 PEX14 PEX8 PEX4 AAT2 PEX13 PEX6 PEX15* | 9 | 40 |
| Cytoplasm | 0005737 | 3.94E-06 | *MRPL16 VID24 MCX1 APM3 BSD2 STP22 PEX19 YDL119C GCS1 RAV2 MRPL7 PEX5 CCC2 SUR2 PEX3 NHX1 SAC2 VPS3 UBP3 MRM2 PEX14 VAM7 PEX8 PEX4 RPN10 MNL1 EPS1 FIS1 MGA2 LYS1 SYS1 APS3 URA2 FBP26 TPK1 RAV1 GEF1 VPS25 VPS24 URA1 UTH1 MRS4 FPS1 SNF7 AAT2 ERG3 RFX1 PEX13 ACO1 RPL26A VPS36 COG8 STV1 VPS20 SSO2 ERG2 SCJ1 SKY1 ATX1 LEM3 PEX6 VPS27 RPP2A PEX15 VPS21 VAM3 TIM18 PRO2 SNF8 VPS28 CIN2 YME1 VPS4* | 73 | 1810 |
| Peroxisomal membrane | 0005778 | 6.98E-06 | *PEX3 PEX14 PEX8 PEX13 PEX15* | 5 | 12 |
| Intracellular | 0005622 | 1.16E-04 | *MRPL16 VID24 MCX1 APM3 BSD2 STP22 SNT1 PEX19 YDL119C GCS1 GRX3 DPB4 RPA14 RAV2 MRPL7 PEX5 CHL4 CCC2 PMP3 SUR2 PEX3 SXM1 NHX1 SAC2 VPS3 RAD23 FTR1 UBP3 CDC26 SGF73 MRM2 PEX14 VAM7 PEX8 PEX4 OPI1 RRM3 RPN10 MNL1 SKN7 EPS1 FIS1 XBP1 MGA2 LYS1 SYS1 APS3 URA2 FBP26 TPK1 RAV1 GEF1 ISY1 VPS25 VPS24 URA1 SET3 UTH1 MRS4 SRP40 FPS1 BRE2 SNF7 AAT2 ERG3 RFX1 PEX13 FRE1 ACO1 RPL26A VPS36 COG8 STV1 FET3 VPS20 SSO2 ERG2 SCJ1 SKY1 PHO23 ATX1 LEM3 PEX6 VPS27 RPP2A PEX15 VPS21 VAM3 LEO1 TIM18 PRO2 SNF8 VPS28 SPP1 UME1 CIN2 YME1 VPS4* | 98 | 2921 |
| Cell | 0005623 | 1.75E-04 | *LTE1 MRPL16 VID24 ARL1 MCX1 APM3 BSD2 STP22 SNT1 PEX19 YDL119C GCS1 GRX3 DPB4 RPA14 RAV2 MRPL7 PEX5 CHL4 CCC2 PMP3 SUR2 PEX3 SXM1 NHX1 SAC2 VPS3 RAD23 FTR1 UBP3 CDC26 SGF73 MRM2 PEX14 VAM7 PEX8 PEX4 OPI1 RRM3 RPN10 MNL1 SKN7 EPS1 FIS1 XBP1 MGA2 LYS1 SYS1 APS3 KHA1 URA2 FBP26 TPK1 RAV1 GEF1 ISY1 VPS25 VPS24 URA1 SET3 UTH1 MRS4 SRP40 FPS1 BRE2 SNF7 AAT2 ERG3 RFX1 PEX13 FRE1 ACO1 RPL26A VPS36 COG8 STV1 FET3 VPS20 SSO2 ERG2 SCJ1 SKY1 PHO23 ATX1 LEM3 PEX6 VPS27 RPP2A PEX15 VPS21 VAM3 LEO1 TIM18 PRO2 SNF8 ARL3 VPS28 SPP1 UME1 CIN2 YME1 VPS4* | 102 | 3106 |
| AP-3 adaptor complex | 0030123 | 3.76E-03 | *APM3 APS3* | 2 | 4 |
| Vacuolar membrane | 0005774 | 6.58E-03 | *RAV2 VAM7 RAV1 STV1 VAM3* | 5 | 47 |
| Hydrogen-translocating V-type ATPase complex | 0016471 | 7.16E-03 | *RAV2 RAV1 STV1* | 3 | 16 |
| Membrane | 0016020 | 7.28E-03 | *VID24 YDL119C RAV2 PMP3 PEX3 FTR1 PEX14 VAM7 PEX8 EPS1 FIS1 MGA2 SYS1 KHA1 RAV1 UTH1 FPS1 PEX13 FRE1 STV1 FET3 SSO2 LEM3 PEX15 VAM3 TIM18 ARL3 YME1* | 28 | 683 |
