# Supplementary material for: Novel insights into iron metabolism by integrating deletome and transcriptome analysis in an iron deficiency model of the yeast Saccharomyces cerevisiae
Source: BMC Genomics. 2009 Mar 25;10:130. doi: 10.1186/1471-2164-10-130 (PMC2669097; doi:10.1186/1471-2164-10-130)
Supplement: Additional file 5 — Functional identification of transcriptional response networks in iron deficiency. All 13 hubs from figure 2A are individually shown with their transcriptional targets, colored in green if deletion of the gene resulted in mutant strain sensitivity to BPS, or red if resistance. [file 1471-2164-10-130-S5.pdf]

Novel Insights into iron metabolism by integrating deletome and transcriptome analysis in an iron deficiency model of the yeast Saccharomyces cerevisiae

Jo, Kim, Oh, *et al.* (2009)

Additional File 5: Functional identification of transcriptional response networks in iron deficiency

Click on transcription factor to visualize interactions

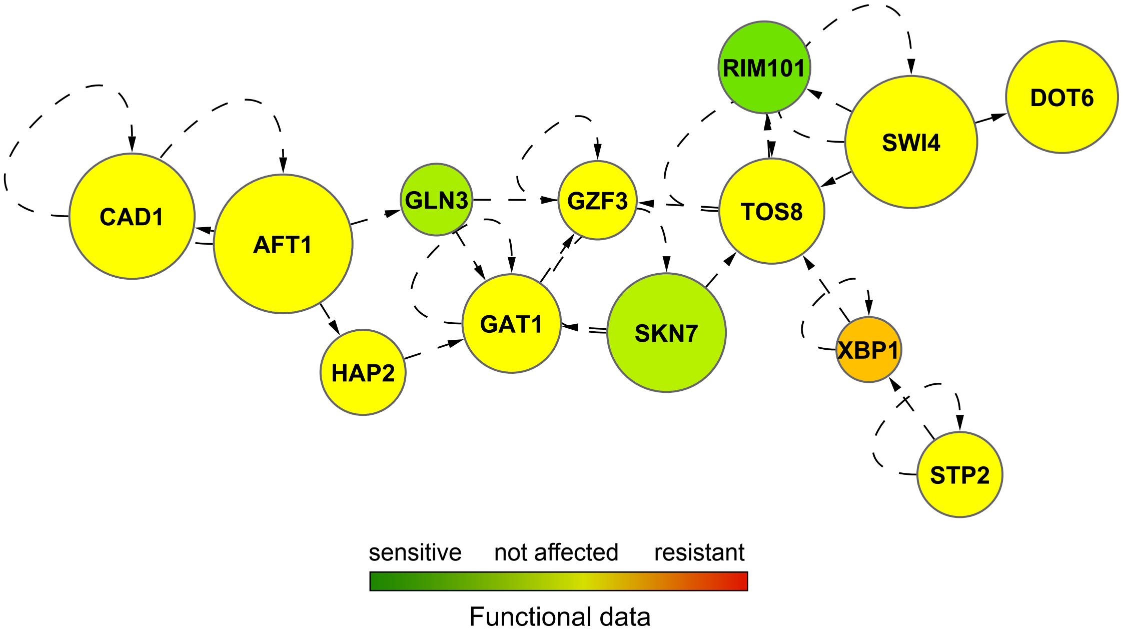

Next page

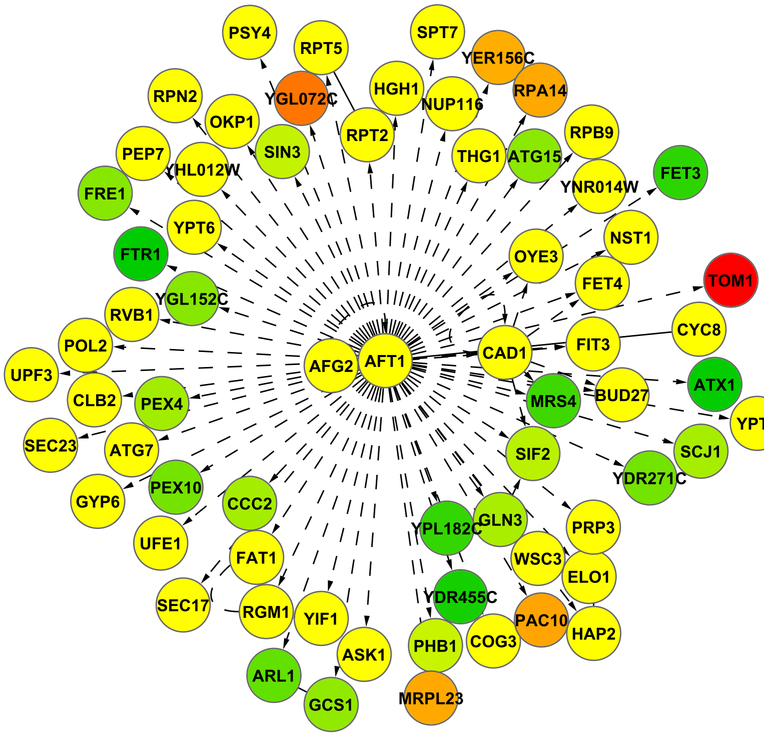

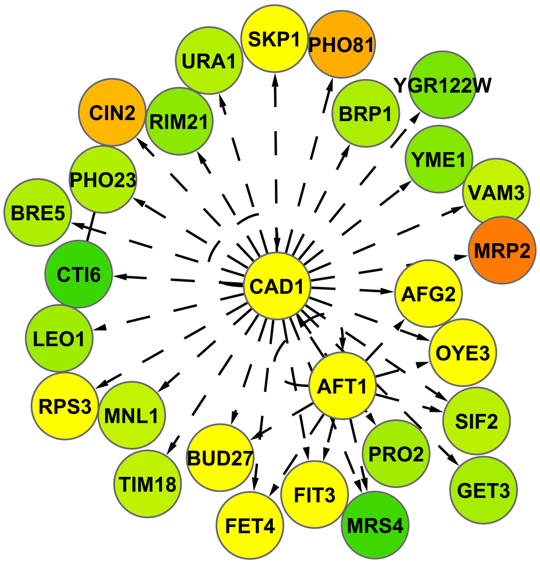

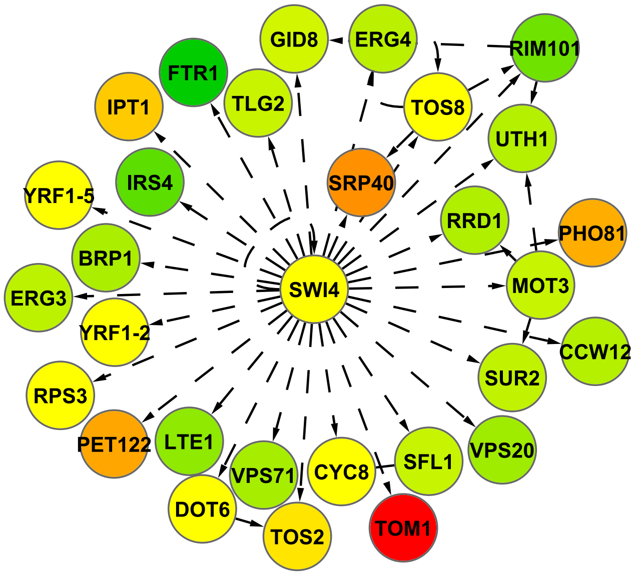

Back to main network

Next page

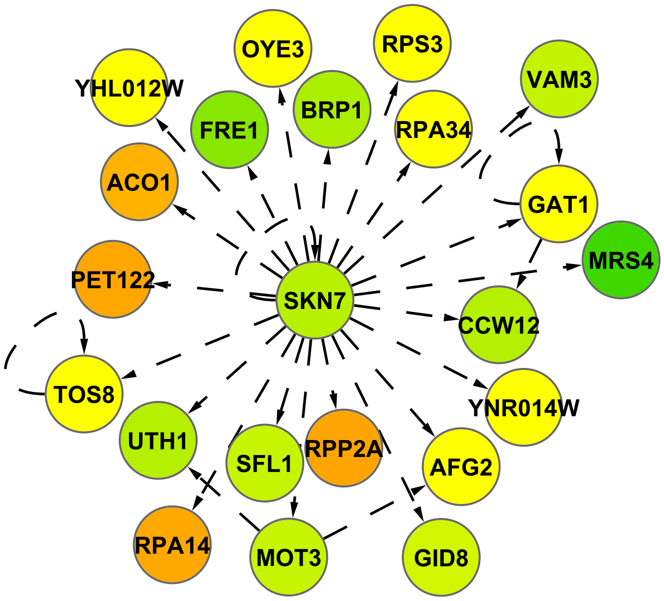

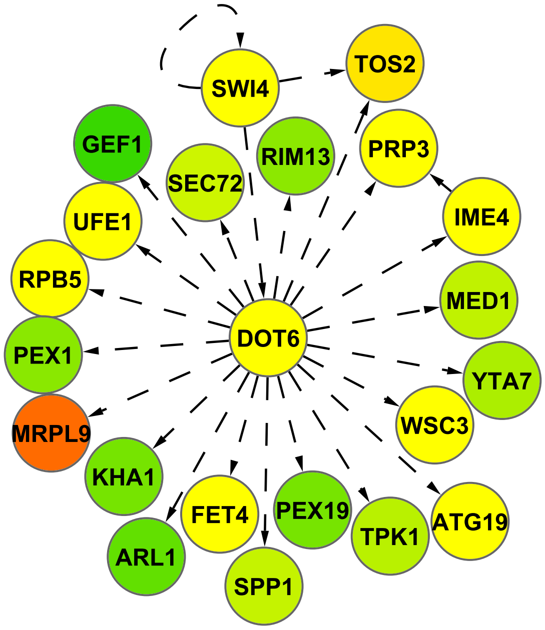

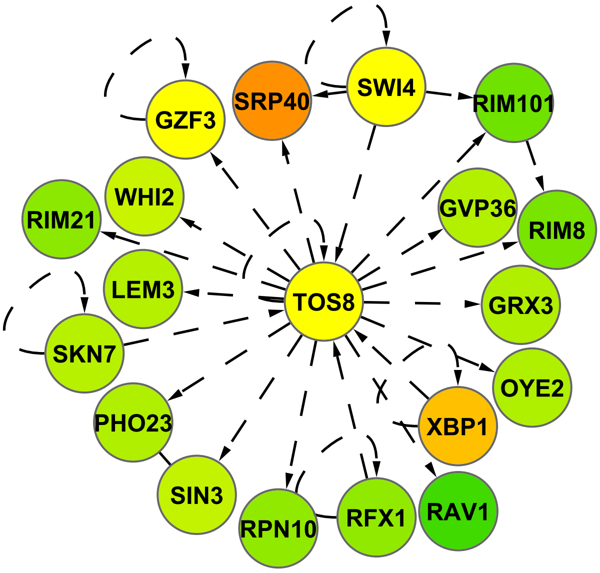

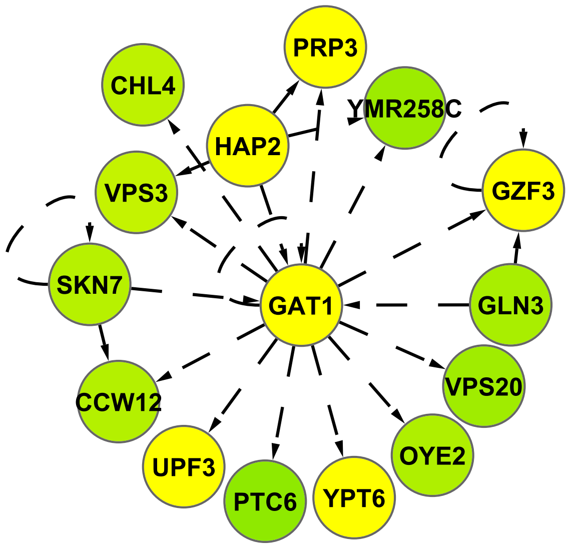

Back to main network

Next page

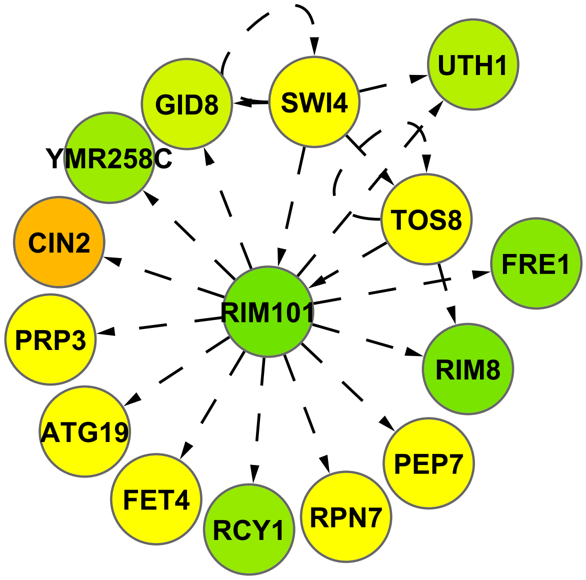

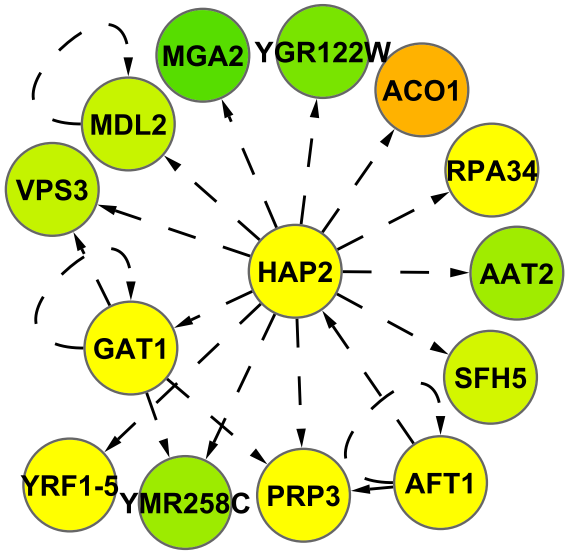

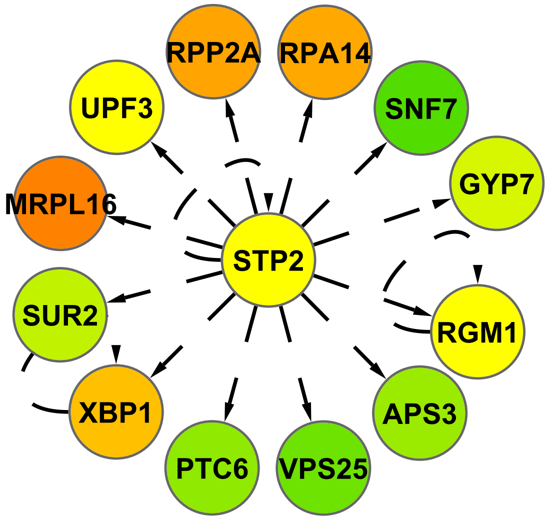

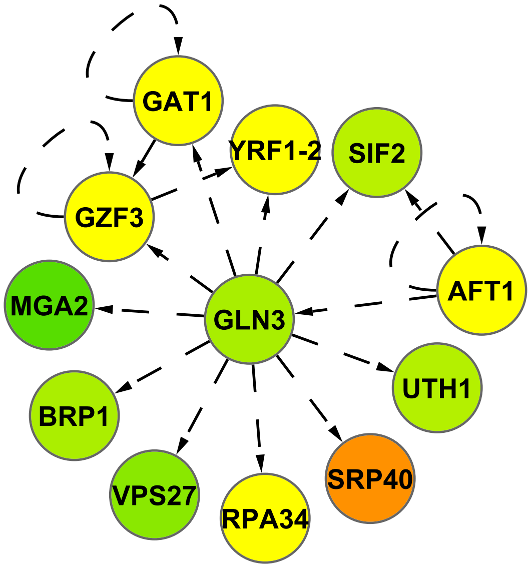

Back to main network

Next page

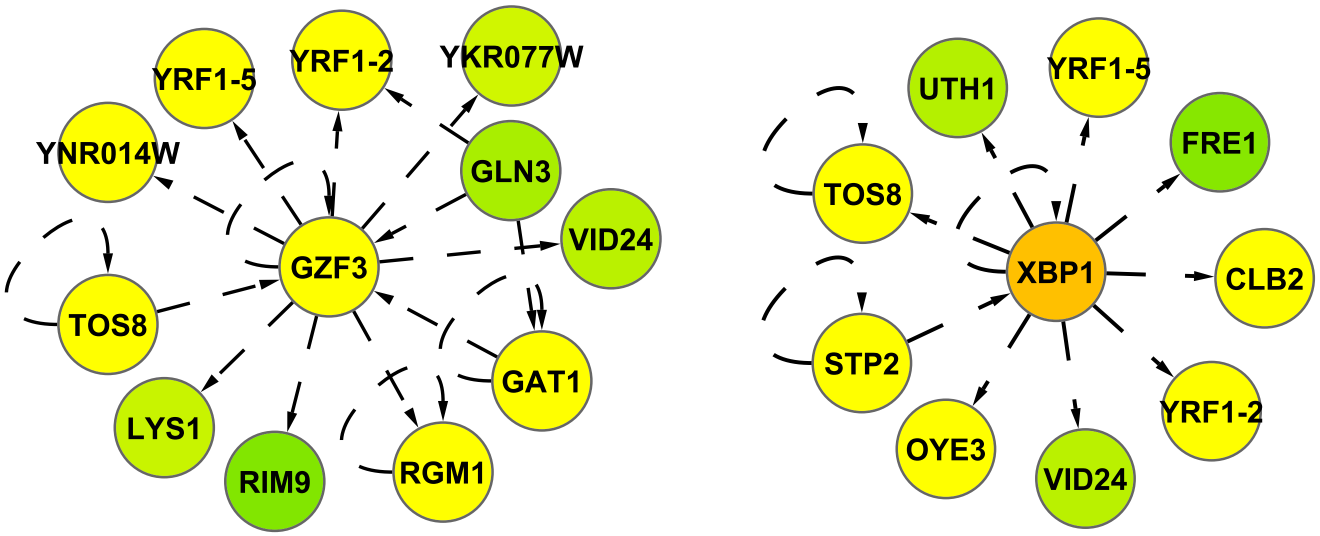

Back to main network
