# Supplementary material for: Novel insights into iron metabolism by integrating deletome and transcriptome analysis in an iron deficiency model of the yeast Saccharomyces cerevisiae
Source: BMC Genomics. 2009 Mar 25;10:130. doi: 10.1186/1471-2164-10-130 (PMC2669097; doi:10.1186/1471-2164-10-130)

Novel insights into iron metabolism by integrating deletome and transcriptome analysis in an iron deficiency model of the yeast *Saccharomyces cerevisiae*

Jo, Kim, Oh, *et al.* (2009)

Additional File 6: Hierarchical clustering analysis of the genes identified by functional profiling in iron deficiency.

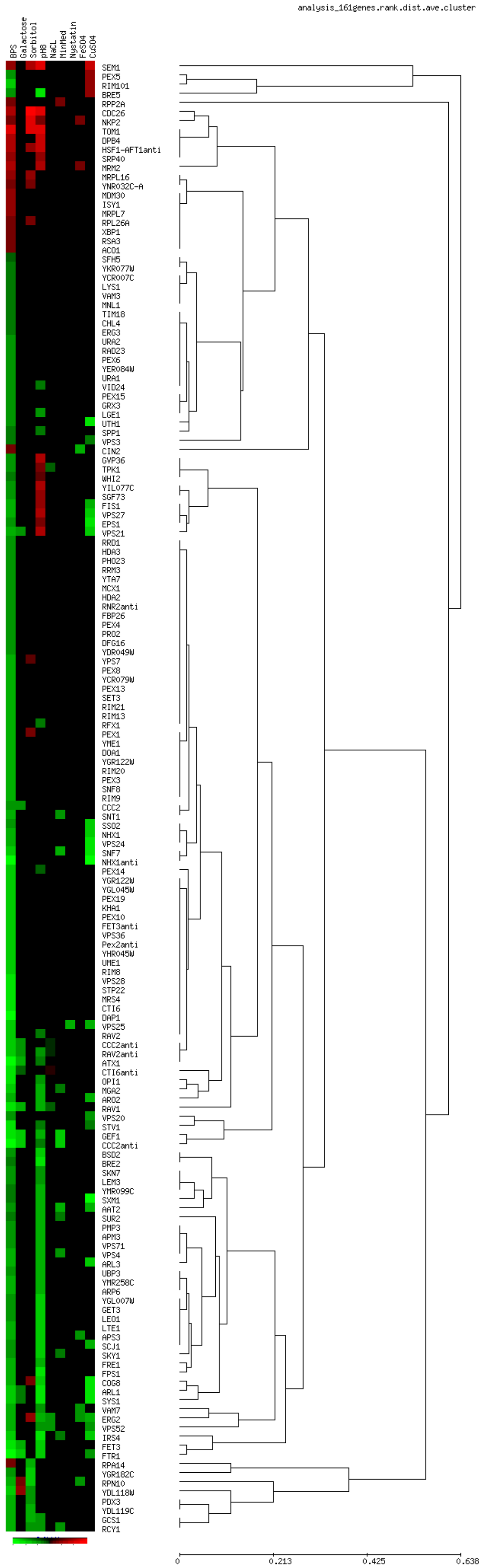

Supplement: Additional file 6 — Hierarchical clustering analysis of the genes identified by functional profiling in iron deficiency under diverse growth conditions. Cluster shows the reanalyzed data from previous functional studies of yeast mutants compared to data from this study. [file 1471-2164-10-130-S6.pdf]
