# Supplementary material for: Novel insights into iron metabolism by integrating deletome and transcriptome analysis in an iron deficiency model of the yeast Saccharomyces cerevisiae
Source: BMC Genomics. 2009 Mar 25;10:130. doi: 10.1186/1471-2164-10-130 (PMC2669097; doi:10.1186/1471-2164-10-130)
Supplement: Additional file 7 — Differentially-expressed genes in nutritional iron-deficient yeast model. Yeast wild type was treated with 100 μM BPS for 1 hour. Genes that were up or down-regulated in at least two out of three independent experiments are listed with average log2 expression values. [file 1471-2164-10-130-S7.pdf]

`

Additional File 7: Differentially-expressed genes in nutritional iron-deficient yeast model. Yeast wild type was treated with 100 μM BPS for 1 hour. Genes that were up or down-regulated in at least two out of three independent experiments are listed. The average expression level is shown in logarithmic scale with base of two. Genes were categorized according to their cellular functions using the GO biological process from Funspec (http://funspec.med.utoronto.ca/).

Up regulated genes (n = 100)

Gene ID	Gene Name	Average expression level	Localization	Function/ Activity
Carbohydrate metabolism (4)				
YAL038W	*CDC19*	0.83	Cytoplasm; Nucleus	Pyruvate kinase, catalyzes final step in glycolysis
YBR110W	*ALG1*	0.33	ER	Beta-mannosyltransferase involved in protein N-glycosylation
YCL040W	*GLK1*	0.49	Cytoplasm	Glucokinase
YGR254W	*ENO1*	0.8	Cytoplasm	Enolase in glycolysis
Iron metabolism (17)				
YDR270W	*CCC2*	0.63	Golgi	Copper-transporting P-type ATPase
YDR534C	*FIT1*	1.46	Cell wall	Cell wall mannoprotein of iron transport facilitator
YEL065W	*SIT1*	1.34	Endosome	Siderophore iron (Ferrioxamine B) permease
YFL041W	*FET5*	0.57	Vacuole	Multicopper oxidase involved in ferrous iron transport
YGL071W	*AFT1*	0.52	Nucleus; Cytoplasm	Iron responsive transcription activator that regulates genes involved in iron homeostasis
YKL220C	*FRE2*	0.85	Plasma membrane	Ferric and cupric reductase
YKR052C	*MRS4*	0.5	Mitochondria	Member of the mitochondrial carrier family of membrane transporters involved in iron transport
YLR136C	*TIS11*	1.38	ER	Zinc finger containing protein that belongs to iron regulon
YLR205C	*HMX1*	0.89	Unknown	Homology to heme oxygenases
YLR214W	*FRE1*	0.93	Plasma membrane	Ferric and cupric reductase

`

Gene ID	Gene Name	Average expression level	Localization	Function/ Activity
Iron metabolism (cont'd)				
YMR058W	*FET3*	0.76	Plasma membrane	Cell surface ferroxidase, required for high-affinity ferrous iron uptake
YMR319C	*FET4*	0.5	Plasma membrane	Low-affinity iron (copper and zinc) transport protein
YOL158C	*ARN4*	1.23	Endosome	Protein involved in iron uptake via a siderophore enterobactin
YOR381W	*FRE3*	1.18	Plasma membrane	Ferric reductase involved in siderophore iron transport
YOR382W	*FIT2*	2.13	Cell wall	Cell wall mannoprotein of iron transport facilitator
YOR383C	*FIT3*	2.02	Cell wall	Cell wall mannoprotein of iron transport facilitator
YOR384W	*FRE5*	1.27	Unknown	Protein with similarity to Fre2p
Lipid metabolism (2)				
YGL055W	*OLE1*	0.54	ER	Delta-9-fatty acid desaturase for synthesis of unsaturated fatty acids
YJR073C	*OPI3*	0.47	ER	Phospholipid-N-methyltransferase in the phosphatidylcholine biosynthesis pathway
Metal homeostasis (4)				
YGL255W	*ZRT1*	1.13	Plasma membrane	High-affinity zinc transport protein
YHR175W	*CTR2*	0.49	Vacuole	Low-affinity copper transport protein
YKL175W	*ZRT3*	0.52	Vacuole	Vacuolar membrane protein involved in zinc transport and the regulation of zinc storage
YLR034C	*SMF3*	0.46	Vacuole	Metal transporter
Nucleotide metabolism (15)				
YBL002W	*HTB2*	0.69	Nucleus	Histone H2B
YBL005W-A	YBL005W-A	0.91	Nucleus	TyA Gag protein
YBL034C	*STU1*	0.85	Nucleus	Suppressor of beta-tubulin mutation that is required for assembly of the mitotic spindle
YBL101W-A	YBL101W-A	0.50	Nucleus	TyA Gag protein
YBR012W-A	YBR012W-A	1.08	Nucleus	TyA Gag protein
YDR118W	*APC4*	0.39	Nucleus	Component of the anaphase-promoting complex
YDR263C	*DIN7*	0.38	Mitochondria	Nuclease with a role in stabilizing the mitochondrial genome, expression is induced by DNA damage

`

Gene ID	Gene Name	Average expression level	Localization	Function/ Activity
Nucleotide metabolism (cont'd)				
YDR423C	*CAD1*	0.4	Cytoplasm; Nucleus	Transcriptional activator of the basic leucine zipper (bZIP) family, involved in regulation of multidrug resistance
YEL042W	*GDA1*	0.52	Golgi	Guanosine diphosphatase
YHL027W	*RIM101*	0.76	Cytoplasm; Nucleus	Transcription factor involved in induction of IME1, IME2, DIT1, and DIT2 transcription, has three C2H2-type zinc fingers
YIL126W	*STH1*	0.34	Nucleus	Component of abundant chromatin remodeling complex
YJR105W	*ADO1*	0.53	Cytoplasm	Adenosine kinase
YKL043W	*PHD1*	0.45	Cytoplasm; Nucleus	Transcription factor involved in regulation of filamentous growth
YMR287C	*MSU1*	0.36	Mitochondria	Component of a mitochondrial 3'-5' exonuclease complex
YML039W	YML039W	0.31	nucleus	TyB Gag-Pol protein
Others (9)				
YAL012W	*CYS3*	0.42	Cytoplasm	Cystathionine gamma-lyase in sulfur assimilation pathway
YDL234C	*GYP7*	0.45	Unknown	GTPase-activating protein for Ypt7p
YDR264C	*AKR1*	0.73	Plasma membrane; Cytoplasm	Protein with palmitoyl transferase activity and ankyrin repeats
YER024W	*YAT2*	1.17	Cytosol; Mitochondrion	Protein with carnitine acetyltransferase function
YLR109W	*AHP1*	0.72	Cytosol; Mitochondrion; Peroxisome	Alkyl hydroperoxide reductase
YML121W	*GTR1*	0.99	Cytoplasm	GTP-binding protein involved in the function of the Pho84p phosphate transporter
YNL192W	*CHS1*	0.46	Membrane	Chitin synthase I
YOL016C	*CMK2*	0.59	Cytoplasm	Calcium/calmodulin-dependent serine/threonine protein kinase type II
YPL171C	*OYE3*	0.44	Cytoplasm	NADPH dehydrogenase, isoform 3
Protein metabolism (5)				
YBR118W	*TEF2*	0.76	Ribosome	Translation elongation factor
YGL123W	*RPS2*	0.66	Cytoplasm	Ribosomal protein S2

`

Gene ID	Gene Name	Average expression level	Localization	Function/ Activity
Protein metabolism (cont'd)				
YJL138C	*TIF2*	0.69	Cytoplasm; Ribosome	Translation initiation factor 4A
YLR121C	*YPS3*	0.31	Plasma membrane; Cell wall	GPI-anchored aspartic protease
YPL154C	*PEP4*	0.44	Vacuole	Proteinase A, aspartyl protease required for activation of various degradative enzymes
Stress (2)				
YDR171W	*HSP42*	0.53	Cytoplasm	Heat shock protein
YGL073W	*HSF1*	2.18	Nucleus	Heat shock transcription factor
Transporters (6)				
YEL063C	*CAN1*	0.79	Plasma membrane	Permease for basic amino acids
YGL006W	*PMC1*	0.33	Vacuole	Ca2+-transporting P-type ATPase
YGR138C	*TPO2*	0.43	Vacuole; Plasma membrane	Polyamine transporter
YHL035C	*YHL035C*	0.83	Membrane	Member of the ATP-binding cassette (ABC) superfamily
YJL094C	*KHA1*	0.4	Plasma membrane	Putative H+/K+ antiporter
YNL003C	*PET8*	0.33	Mitochondria	Protein of the mitochondrial carrier (MCF) family of membrane transporters, has similarity to Mrs4p and Mrs3p
Functionally unknown genes (36)				
YAL061W	*YAL061W*	0.38	Unknown	Unknown
YAL065C	*YAL065C*	0.31	Unknown	Unknown
YAR068W	*YAR068W*	0.41	Unknown	Unknown
YBL111C	*YBL111C*	0.39	Unknown	Unknown
YBR005W	*YBR005W*	0.38	Unknown	Unknown
YBR047W	*YBR047W*	0.56	Unknown	Unknown
YCR007C	*YCR007C*	0.47	Unknown	Unknown
YDL048C	*STP4*	0.34	Chromatin	Protein with strong similarity to Stp1p, which is involved in tRNA splicing and branched-chain amino acid uptake
YDL124W	*YDL124W*	0.56	Unknown	Unknown

`

Gene ID	Gene Name	Average expression level	Localization	Function/ Activity
Functionally unknown genes (cont'd)				
YDR033W	*MRH1*	0.73	Plasma membrane; Nucleus	Protein with similarity to Yro2p
YDR271C	*YDR271C*	0.82	Unknown	Unknown
YEL076C-A	*YEL076C-A*	0.34	Unknown	Unknown
YEL076W-C	*YEL076W-C*	0.34	Unknown	Unknown
YER053C	*YER053C*	0.32	Vacuole	Protein with homology to mitochondrial phosphate transporter, but not itself
YFL010C	*WWM1*	0.43	Cytoplasm; Nucleus	Protein that may be involved in regulation of metacaspase (Mca1p) function in control of cell proliferation
YFR024C	*YFR024C*	0.48	Unknown	Unknown
YFR024C-A	*LSB3*	0.42	Unknown	Protein with high similarity to S. cerevisiae Ysc84p
YGR053C	*YGR053C*	0.43	Unknown	Unknown
YHR214W-A	*YHR214W-A*	0.47	Unknown	Unknown
YIL028W	*YIL028W*	0.48	Unknown	Unknown
YIL146C	*ECM37*	0.52	Unknown	Protein possibly involved in cell wall structure or biosynthesis
YJL078C	*PRY3*	0.35	Cell wall; Cytoplasm	Protein with similarity to plant pathogenesis-related proteins
YKL153W	*YKL153W*	0.62	Unknown	Unknown
YLR126C	*YLR126C*	0.54	Unknown	Unknown
YLR194C	*YLR194C*	0.59	Unknown	Unknown
YLR280C	*YLR280C*	0.46	Unknown	Unknown
YLR297W	*YLR297W*	0.35	Unknown	Unknown
YLR327C	*YLR327C*	0.41	Unknown	Unknown
YLR443W	*ECM7*	0.78	Unknown	Protein possibly involved in cell wall structure or biosynthesis
YMR084W	*YMR084W*	0.72	Unknown	Unknown
YMR251W	*YMR251W*	1.4	Unknown	Unknown
YNL143C	*YNL143C*	0.54	Unknown	Unknown
YOL098C	*YOL098C*	0.7	Unknown	Unknown
YOR247W	*SRL1*	0.55	Cell wall	Protein with similarity to Svs1p
YOR291W	*YOR291W*	0.68	Membrane	Unknown
YPR157W	*YPR157W*	0.43	Unknown	Unknown

`

Down-regulated genes (n = 42)

Gene ID	Gene Name	Average expression level	Localization	Function/ Activity
Energy metabolism (5)				
YDR178W	*SDH4*	-0.42	Mitochondria	Succinate dehydrogenase membrane anchor subunit
YGR174C	*CBP4*	-0.49	Mitochondria	Ubiquinol-cytochrome c reductase assembly factor
YKL087C	*CYT2*	-0.32	Mitochondria	Cytochrome c1 heme lyase
YLR304C	*ACO1*	-0.82	Mitochondria	Aconitase
YPR191W	*QCR2*	-0.58	Mitochondria	Ubiquinol cytochrome-c reductase core protein 2
Nucleotide metabolism (5)				
YGR092W	*DBF2*	-0.49	Spindle pole body; Nucleus; Bud neck	Serine/threonine protein kinase related to Dbf20p, required for events in anaphase/telophase
YKL109W	*HAP4*	-0.45	Nucleus	Transcription factor with acidic activation domain, component of Hap2p-Hap3p-Hap4p-Hap5p complex involved in activation of CCAAT box-containing genes
YLR398C	*SKI2*	-0.56	Cytoplasm; Nucleolus	Helicase (probable)
YML061C	*PIF1*	-0.47	Nucleus; Mitochondria	Single-stranded DNA-dependent ATPase and 5'-3' DNA helicase required for maintenance and repair of mitochondrial DNA, also functions in nucleus to regulate telomere length
YPL001W	*HAT1*	-0.49	Cytoplasm; Nucleus	Histone acetyltransferase
Others (7)				
YBR093C	*PHO5*	-0.38	Cell wall; Periplasmic space	Acid phosphatase in phosphate metabolism
YGL032C	*AGA2*	-0.48	Shmoo tip; ER; Bud tip; Cell wall	a-agglutinin adhesion subunit involved in cellular fusion
YHR096C	*HXT5*	-0.42	Plasma membrane	Hexose transporter
YLL009C	*COX17*	-0.72	Mitochondrion; Cytoplasm	Protein involved in delivery of copper ions to mitochondrial cytochrome oxidase

`

**Gene ID**	**Gene Name**	**Average expression level**	**Localization**	**Function/ Activity**
Others (cont'd)				
YMR015C	*ERG5*	-0.36	Cytoplasm; Microsome; Nucleus	Cytochrome P450-involved in C-22 denaturation of the ergosterol side-chain
YNL111C	*CYB5*	-0.34	ER	Cytochrome b5 in sterol metabolism
YPL170W	*DAP1*	-0.32	Membrane	Protein involved in ergosterol biosynthesis, response to UV, mitochondrial genome maintenance, and telomere maintenance, member of the membrane-associated progesterone receptor family
Protein metabolism (5)				
YDL184C	*RPL41A*	-0.5	Cytosol	Ribosomal protein L41A
YGL009C	*LEU1*	-1.19	Cytosol	3-Isopropylmalate dehydratase in leucine biosynthesis
YGR084C	*MRP13*	-0.89	Mitochondria	Mitochondrial ribosomal protein
YIL018W	*RPL2B*	-0.63	Cytosol	Ribosomal protein L2B
YLR325C	*RPL38*	-0.52	Cytosol	Ribosomal protein L38
Stress (2)				
YER174C	*GRX4*	-0.64	Unknown	Glutaredoxin
YKR066C	*CCP1*	-0.93	Mitochondria	Cytochrome c peroxidase
Functionally unknown genes (18)				
YBL044W	*YBL044W*	-0.53	Unknown	Unknown
YDL162C	*YDL162C*	-0.9	Unknown	Unknown
YDR154C	*YDR154C*	-0.46	Unknown	Unknown
YDR494W	*RSM28*	-0.31	Unknown	Unknown
YER156C	*YER156C*	-0.42	Unknown	Unknown
YFL012W	*YFL012W*	-0.76	Unknown	Unknown
YGR226C	*YGR226C*	-0.67	Unknown	Unknown
YGR270W	*YTA7*	-1.54	Unknown	ATPase activity
YJL200C	*YJL200C*	-0.38	Unknown	Protein with homology to aconitase
YKL208W	*CBT1*	-0.53	Unknown	Unknown
YNL109W	*YNL109W*	-0.67	Unknown	Unknown
YNL120C	*YNL120C*	-0.49	Unknown	Unknown
YNL122C	*YNL122C*	-0.46	Unknown	Unknown
YNL303W	*YNL303W*	-0.45	Unknown	Unknown
YNR025C	*YNR025C*	-0.33	Unknown	Unknown
YOL109W	*YOL109W*	-0.5	Unknown	Unknown
YOR338W	*YOR338W*	-0.31	Unknown	Unknown
YPL182C	*YPL182C*	-0.29	Unknown	Unknown
