# Supplementary material for: Novel insights into iron metabolism by integrating deletome and transcriptome analysis in an iron deficiency model of the yeast Saccharomyces cerevisiae
Source: BMC Genomics. 2009 Mar 25;10:130. doi: 10.1186/1471-2164-10-130 (PMC2669097; doi:10.1186/1471-2164-10-130)
Supplement: Additional file 8 — Differentially-expressed genes in nutritional iron-deficient yeast model, data for each of three independent experiments. Yeast wild type was treated with 100 μM BPS for 1 hour. Genes that were up or down-regulated in any of three independent experiments are listed with log2 expression values. [file 1471-2164-10-130-S8.pdf]

Additional File 8: Differentially-expressed genes in wild type yeast after 1 h treatment with 100 μM BPS. The genes were differentially expressed in at least two out of three independent experiments at a p-value of less than 0.1. Expression data for each experiment (#1, 2 and 3) are given in logarithmic scale with base of two. Localization of gene products and their functions are from the *Saccharomyces* Genome Database (http://www.yeastgenome.org) and MIPS-Comprehensive Yeast Genome Database (http://mips.gsf.de/proj/yeast/CYGD/db/index.html).

UP REGULATED GENES (n = 100)

Gene ID	Gene Name	#1	#2	#3	Localization	Function
Carbohydrate metabolism (4)						
YAL038W	*CDC19*	1.03	0.59	0.88	cytosol	pyruvate kinase
YBR110W	*ALG1*		0.36	0.3	membrane	beta-1,4-mannosyltransferase
YCL040W	*GLK1*	0.69	0.44	0.33	cytosol	glucokinase
YGR254W	*ENO1*	0.74		0.86	Cytoplasm	Enolase
Iron metabolism (17)						
YDR270W	*CCC2*		0.51	0.75	Golgi	copper-transporting P-type ATPase
YDR534C	*FIT1*		1.5	1.41	Cell wall	Cell wall protein involved in iron uptake
YEL065W	*SIT1*	1.4	1.43	1.18	Endosome	Siderophore Iron Transport
YFL041W	*FET5*		0.62	0.51	vacuole	multicopper oxidase
YGL071W	*AFT1*	0.66	0.37		Nucleus; cytoplasm	iron responsive transcription activator
YKL220C	*FRE2*	0.69	0.8	1.07	Plasma membrane	Ferric reductase
YKR052C	*MRS4*		0.48	0.51	mitochondria	mitochondrial carrier protein
YLR136C	*TIS11*	0.92	1.62	1.59	Unknown	Zinc finger containing protein
YLR205C	*HMX1*		0.96	0.82	membrane	Homology to heme oxygenases
YLR214W	*FRE1*	1.72	0.61	0.46	Plasma membrane	Ferric (and cupric) reductase
YMR058W	*FET3*	0.97	0.64	0.68	Plasma membrane	multicopper oxidase
YMR319C	*FET4*	0.5	0.54	0.46	Plasma membrane	low-affinity iron transporter
YOL158C	*ARN4*	0.77	1.6	1.31	Endosome	Siderophore transporter
YOR381W	*FRE3*		1.36	0.99	plasma membrane	Ferric reductase activity
YOR382W	*FIT2*	1.08	2.65	2.66	Cell wall	Cell wall protein involved in iron transport

Gene ID	Gene Name	#1	#2	#3	Localization	Function
Iron metabolism (cont'd)						
YOR383C	*FIT3*	1.07	2.61	2.38	Cell wall	Cell wall protein involved in iron transport
YOR384W	*FRE5*		1.27	1.27	Unknown	Ferric reductase activity
Lipid metabolism (2)						
YGL055W	*OLE1*	0.62	0.45		ER	delta-9-fatty acid desaturase
YJR073C	*OPI3*	0.63	0.3		ER	phosphatidylcholine biosynthesis
Metal homeostasis (4)						
YGL255W	*ZRT1*	1.61	0.9	0.88	Plasma membrane	High-affinity zinc transport protein
YHR175W	*CTR2*		0.54	0.44	vacuole	Putative low-affinity copper transport protein
YKL175W	*ZRT3*	0.56	0.47		vacuole	Zinc transporter
YLR034C	*SMF3*		0.47	0.44	vacuole	Metal transporter (iron)
Nucleotide metabolism (15)						
YBL002W	*HTB2*		0.53	0.85	Nucleus	Histone H2B
YBL005W-A	*YBL005W-A*	1.17	0.62	0.94	nucleus	TyA Gag protein
YBL034C	*STU1*	0.67	0.92	0.97	cytoskeleton	Spindle formation
YBL101W-A	*YBL101W-A*		0.48	0.52	nucleus	TyA Gag protein
YBR012W-A	*YBR012W-A*	1.23		0.92	nucleus	TyA Gag protein
YDR118W	*APC4*		0.29	0.48	Nucleus	anaphase promoting complex (APC) subunit
YDR263C	*DIN7*		0.31	0.44	mitochondria	DNA damage inducible gene
YDR423C	*CAD1*		0.38	0.42	Nucleus	basic leucine zipper transcription factor responsive to cadimium
YEL042W	*GDA1*		0.51	0.53	Golgi apparatus	guanosine diphosphatase
YHL027W	*RIM101*	0.71	0.96	0.61	Nucleus	transcriptional activator required for entry into meiosis
YIL126W	*STH1*		0.25	0.43	Nucleus	helicase related protein
YJR105W	*ADO1*		0.43	0.62	Unknown	adenosine kinase
YKL043W	*PHD1*		0.41	0.49	Nucleus	putative TF
YML039W	*YML039W*		0.32	0.31	nucleus	TyB Gag-Pol protein
YMR287C	*MSU1*		0.3	0.41	mitochondria	Protein essential for mitochondrial biogenesis; exonuclease component
Others (9)						
YAL012W	*CYS3*	0.53	0.3		Unknown	cystathionine gamma-lyase
YDL234C	*GYP7*		0.44	0.46	Unknown	GTPase activating protein (GAP)
YDR264C	*AKR1*	0.92	0.77	0.5	membrane	ankyrin repeat-containing protein
YER024W	*YAT2*	1.02	1.28	1.21	Unknown	carnitine acetyltransferase
YLR109W	*AHP1*	0.91	0.77	0.47	Cytoplasm	thioredoxin peroxidase
YML121W	*GTR1*	1.03		0.94	Cytoplasm	putative small GTPase
YNL192W	*CHS1*	0.54	0.37		Plasma membrane	Chitin synthase 1

Gene ID	Gene Name	#1	#2	#3	Localization	Function
Others (cont'd)						
YOL016C	*CMK2*		0.46	0.72	Unknown	calmodulin-dependent protein kinase
YPL171C	*OYE3*		0.44	0.44	Cytoplasm	NADPH dehydrogenase
Protein metabolism (5)						
YBR118W	*TEF2*	0.84	0.6	0.83	Ribosome	translation elongation factor
YGL123W	*RPS2*		0.55	0.77	Cytoplasm	ribosomal protein
YJL138C	*TIF2*	0.56		0.81	Cytoplasm	translation initiation activity
YLR121C	*YPS3*		0.31	0.3	Plasma membrane	GPI-anchored aspartic protease
YPL154C	*PEP4*		0.44	0.44	Vacuole	vacuolar proteinase A
Stress (2)						
YDR171W	*HSP42*	0.55	0.51	0.52	Cytoplasm	Heat shock protein
YGL073W	*HSF1*	1.9	2.33	2.32	Nucleus	Heat shock transcription factor
Transporters (6)						
YEL063C	*CAN1*		0.77	0.81	Plasma membrane	arginine permease
YGL006W	*PMC1*		0.31	0.35	Vacuole	Calcium ion transporter
YGR138C	*TPO2*		0.42	0.43	vacuolar membrane; plasma membrane	Polyamine transporter
YHL035C	*YHL035C*		0.85	0.81	membrane	ABC transporter
YJL094C	*KHA1*		0.38	0.42	membrane	putative H+/K+ antiporter
YNL003C	*PET8*		0.32	0.34	mitochondria	Member of family of mitochondrial carrier proteins
Functionally unknown genes (36)						
YAL061W	*YAL061W*		0.37	0.39	Unknown	Unknown
YAL065C	*YAL065C*		0.28	0.34	Unknown	Unknown
YAR068W	*YAR068W*		0.37	0.44	Unknown	Unknown
YBL111C	*YBL111C*	0.53	0.24		Unknown	Unknown
YBR005W	*YBR005W*		0.35	0.4	Unknown	Unknown
YBR047W	*YBR047W*		0.52	0.59	Unknown	Unknown
YCR007C	*YCR007C*	0.58		0.35	Unknown	Unknown
YDL048C	*STP4*		0.32	0.36	Unknown	Involved in pre-tRNA splicing and in uptake of branched-chain amino acids
YDL124W	*YDL124W*		0.57	0.54	Unknown	Unknown
YDR033W	*MRH1*	0.94	0.52		Plasma membrane	Unknown
YDR271C	*YDR271C*		0.8	0.84	Unknown	Unknown
YEL076C-A	*YEL076C-A*		0.32	0.35	Unknown	Unknown
YEL076W-C	*YEL076W-C*		0.3	0.38	Unknown	Unknown

Gene ID	Gene Name	#1	#2	#3	Localization	Function
Functionally unknown genes (cont'd)						
YER053C	*YER053C*		0.27	0.36	vacuole	homology to mitochondrial phosphate transporter, but not itself
YFL010C	*WWM1*	0.52	0.34		Cytoplasm	Unknown
YFR024C	*YFR024C*		0.53	0.43	Unknown	Unknown
YFR024C-A	*LSB3*		0.41	0.42	Unknown	Unknown
YGR053C	*YGR053C*		0.47	0.38	Unknown	Unknown
YHR214W-A	*YHR214W-A*		0.42	0.51	Unknown	Unknown
YIL028W	*YIL028W*		0.58	0.38	Unknown	Unknown
YIL146C	*ECM37*		0.48	0.56	Unknown	Unknown
YJL078C	*PRY3*		0.33	0.36	Cell wall	Unknown
YKL153W	*YKL153W*		0.55	0.68	Unknown	Unknown
YLR126C	*YLR126C*		0.48	0.59	Unknown	Unknown
YLR194C	*YLR194C*		0.68	0.49	Unknown	Unknown
YLR280C	*YLR280C*		0.46	0.46	Unknown	Unknown
YLR297W	*YLR297W*		0.37	0.33	Unknown	Unknown
YLR327C	*YLR327C*		0.36	0.46	Unknown	Unknown
YLR443W	*ECM7*		0.64	0.91	membrane	Cell wall maintenance
YMR084W	*YMR084W*		0.93	0.5	Unknown	Unknown
YMR251W	*YMR251W*		1.51	1.28	Unknown	Unknown
YNL143C	*YNL143C*	0.74		0.34	Unknown	Unknown
YOL098C	*YOL098C*		0.57	0.82	Unknown	Unknown
YOR247W	*SRL1*	0.55	0.54		Unknown	Unknown
YOR291W	*YOR291W*	0.76	0.6	0.91	membrane	Unknown
YPR157W	*YPR157W*		0.51	0.35	Unknown	Unknown

DOWN REGULATED GENES (n = 42)

Gene ID	Gene Name	#1	#2	#3	Localization	Function
Energy metabolism (5)						
YDR178W	*SDH4*	-0.5	-0.32	-0.44	mitochondria	succinate dehydrogenase membrane anchor subunit
YKL087C	*CYT2*		-0.29	-0.34	mitochondria	cytochrome c1 heme lyase
YLR304C	*ACO1*		-0.77	-0.86	mitochondria	aconitase
YNL111C	*CYB5*		-0.29	-0.39	microsome	cytochrome b5
YPR191W	*QCR2*	-0.83		-0.33	mitochondria	ubiquinol cytochrome-c reductase core protein 2
Nucleotide metabolism (5)						
YGR092W	*DBF2*	-0.68		-0.3	Unknown	involved in nuclear division
YKL109W	*HAP4*	-0.68	-0.29	-0.39	Nucleus	Transcriptional activator involved in carbohydrate metabolism
YLR398C	*SKI2*		-0.51	-0.61	cytoplasm	helicase (putative)
YML061C	*PIF1*	-0.53		-0.4	Nucleus; mitochondria	DNA helicase
YPL001W	*HAT1*	-0.53	-0.44		cytoplasm; nucleus	histone acetyltransferase
Others (6)						
YBR093C	*PHO5*		-0.33	-0.43	Cell wall; periplasmic space	Acid phosphatase
YGL032C	*AGA2*	-0.62	-0.39	-0.44	Cell wall	a-agglutinin adhesion subunit
YGR174C	*CBP4*	-0.74	-0.33	-0.4	Mitochondria	Essential for the expression and activity of ubiquinol-cytochrome c reductase
YHR096C	*HXT5*		-0.41	-0.42	Plasma membrane	Hexose transporter
YLL009C	*COX17*	-0.49		-0.95	Cytosol; mitochondria	Intracellular copper transport
YMR015C	*ERG5*		-0.3	-0.41	ER	Cytochrome P450- involved in C-22 denaturation of the ergosterol side-chain
Protein metabolism (5)						
YDL184C	*RPL41A*	-0.55		-0.44	Cytoplasm	Ribosomal protein L41A
YGL009C	*LEU1*		-1.64	-0.74	Cytosol	Leucine biosynthesis

Gene ID	Gene Name	#1	#2	#3	Localization	Function
Protein metabolism (cont'd)						
YGR084C	*MRP13*		-1.41	-0.37	Mitochondria	Mitochondrial ribosomal protein
YIL018W	*RPL2B*	-0.71		-0.55	Cytosol	Ribosomal protein L2B
YLR325C	*RPL38*	-0.66		-0.37	Cytosol	Ribosomal protein L38
Stress (2)						
YER174C	*GRX4*		-0.58	-0.7	Unknown	Glutaredoxin
YKR066C	*CCP1*		-0.89	-0.97	Mitochondria	Cytochrome c peroxidase
Functionally unknown genes (19)						
YBL044W	*YBL044W*		-0.31	-0.75	Unknown	Unknown
YDL162C	*YDL162C*	-1.5	-0.29		Unknown	Unknown
YDR154C	*YDR154C*	-0.55		-0.37	Unknown	Unknown
YDR494W	*RSM28*		-0.28	-0.33	Mitochondria	Mitochondrial ribosomal protein
YER156C	*YER156C*		-0.36	-0.47	Unknown	Unknown
YFL012W	*YFL012W*	-0.86		-0.65	Unknown	Unknown
YGR226C	*YGR226C*		-0.54	-0.79	Unknown	Unknown
YGR270W	*YTA7*		-1.34	-1.73	Unknown	ATPase activity
YJL200C	*YJL200C*		-0.4	-0.35	Unknown	Homology to aconitase
YKL208W	*CBT1*	-0.63	-0.43		Unknown	Unknown
YNL109W	*YNL109W*	-0.83		-0.51	Unknown	Unknown
YNL120C	*YNL120C*	-0.64	-0.34		Unknown	Unknown
YNL122C	*YNL122C*	-0.62		-0.3	Unknown	Unknown
YNL303W	*YNL303W*	-0.47		-0.42	Unknown	Unknown
YNR025C	*YNR025C*		-0.26	-0.39	Unknown	Unknown
YOL109W	*YOL109W*	-0.63		-0.36	Unknown	Unknown
YOR338W	*YOR338W*		-0.32	-0.3	Unknown	Unknown
YPL170W	*DAP1*		-0.28	-0.35	Membrane	Unknown
YPL182C	*YPL182C*		-0.25	-0.33	Unknown	Unknown
