# Supplementary material for: Novel insights into iron metabolism by integrating deletome and transcriptome analysis in an iron deficiency model of the yeast Saccharomyces cerevisiae
Source: BMC Genomics. 2009 Mar 25;10:130. doi: 10.1186/1471-2164-10-130 (PMC2669097; doi:10.1186/1471-2164-10-130)
Supplement: Additional file 9 — Gene Ontology enrichment analysis of expression profiling data for up- and down-regulated genes. Gene Ontology molecular functions, biological processes and cellular components that were significantly enriched with genes identified by expression profiling. [file 1471-2164-10-130-S9.pdf]

Additional File 9: Gene Ontology enrichment analysis of differentially-expressed genes in iron deficiency. Gene Ontology molecular functions, biological processes and cellular components that were significantly enriched with genes identified by expression profiling were identified using Funspec (http://funspec.med.utoronto.ca/). In the tables, *k* represents the number of identified genes and *f* the total number of genes in the category. Only the categories with hypergeometric P < 0.01 are shown.

UP-REGULATED GENES

**GO Molecular Function**					
**Category**	**GO ID**	**p-value**	**In Category from Cluster**	**k**	**f**
Iron ion binding	0005506	9.91E-10	*FIT1 SIT1 FET5 OLE1 AFT1 FRE2 SMF3 HMX1 FRE1 FET3 FET4 FRE3 FIT2 FIT3 FRE5*	15	145
Copper ion transmembrane transporter activity	0005375	1.27E-06	*CCC2 CTR2 FRE2 FRE1 FET4*	5	15
Iron ion transmembrane transporter activity	0005381	2.37E-06	*MRS4 SMF3 FET3 FET4*	4	8
Ferric-chelate reductase activity	0000293	6.95E-06	*FRE2 FRE1 FRE3 FRE5*	4	10
Copper ion binding	0005507	5.36E-05	*CCC2 FET5 CTR2 FRE2 FRE1 FET3*	6	49
Metal ion binding	0046872	0.000297	*CDC19 BDH2 STP4 AKR1 CCC2 FET5 OLE1 AFT1 ENO1 RIM101 FRE2 SMF3 TIS11 HMX1 FRE1 FET3 YOL098C YOR291W FRE3 FRE5*	20	625
Ferroxidase activity	0004322	0.000571	*FET5 FET3*	2	3
Metal ion transmembrane transporter activity	0046873	0.000853	*CCC2 ZRT1 ZRT3*	3	14
P-P-bond-hydrolysis-driven transmembrane transporter activity	0015405	0.001131	*CCC2 YOR291W*	2	4
Primary active transmembrane transporter activity	0015399	0.001131	*CCC2 YOR291W*	2	4
FAD binding	0050660	0.00164	*FRE2 FRE1 FRE3 FRE5*	4	37
Hydrolase activity, acting on acid anhydrides, catalyzing transmembrane movement of substances	0016820	0.001836	*CCC2 PMC1 YOR291W*	3	18

Category	GO ID	p-value	In Category from Cluster	k	f
ATPase activity, coupled to transmembrane movement of ions, phosphorylative mechanism	0015662	0.00736	*CCC2 PMC1 YOR291W*	3	29
Zinc ion transmembrane transporter activity	0005385	0.008036	*ZRT1 ZRT3*	2	10
Antiporter activity	0015297	0.009733	*TPO2 KHA1*	2	11
Potassium ion binding	0030955	0.009733	*CDC19 KHA1*	2	11

**GO Biological Process**					
**Category**	**GO ID**	**p-value**	**In Category from Cluster**	**k**	**f**
Ion transport	0006811	1.00E-14	*MRH1 CCC2 FIT1 SIT1 FET5 PMC1 AFT1 ZRT1 CTR2 KHA1 ZRT3 FRE2 SMF3 FRE1 FET3 FET4 FRE3 FIT2 FIT3 FRE5*	20	117
Iron ion transport	0006826	4.47E-13	*FIT1 SIT1 FET5 FRE2 SMF3 FRE1 FET3 FET4 FRE3 FIT2 FIT3*	11	34
Copper ion import	0015677	2.37E-06	*CTR2 FRE2 FRE1 FET4*	4	8
Siderophore transport	0015891	2.62E-06	*FIT1 FIT2 FIT3*	3	3
Cellular iron ion homeostasis	0006879	9.36E-05	*CCC2 SIT1 YLR126C TIS11 HMX1 FRE3*	6	54
Copper ion transport	0006825	0.000117	*CCC2 CTR2 FRE2 FRE1*	4	19
Intracellular copper ion transport	0015680	0.000207	*CCC2 CTR2 FET4*	3	9
Transport	0006810	0.00024	*CCC2 FIT1 CAN1 SIT1 PIC2 FET5 PMC1 OLE1 ZRT1 TPO2 VMR1 CTR2 KHA1 ZRT3 FRE2 MRS4 SMF3 FRE1 GTR1 FET3 FET4 PET8 YOR291W FRE3 FIT2 FIT3 FRE5 YPR157W*	28	1031
Response to inorganic substance	0010035	0.001131	*OLE1 FET4*	2	4
Oxidation reduction	0055114	0.001388	*BDH2 YDL124W FET5 OLE1 FRE2 AHP1 FRE1 FET3 FRE3 FRE5 OYE3*	11	274
Metal ion transport	0030001	0.001545	*CCC2 ZRT1 ZRT3*	3	17
Zinc ion transport	0006829	0.002158	*ZRT1 ZRT3 FET4*	3	19
Siderophore-iron transport	0015892	0.002778	*SIT1 FRE3*	2	6
Biopolymer biosynthetic process	0043284	0.002869	*CYS3 CDC19 MRH1 ENO1 ECM7 FET3 SRL1*	7	137
Response to starvation	0042594	0.002903	*FIT1 PHD1 ZRT3*	3	21
High-affinity iron ion transport	0006827	0.003853	*AFT1 FET3*	2	7
Cation transport	0006812	0.005397	*PMC1 KHA1 YOR291W*	3	26

GO Cellular Component					
**Category**	**GO ID**	**p-value**	**In Category from Cluster**	**k**	**f**
Integral to membrane	0016021	9.35E-05	*YAL065C RCR1 ALG1 YCR007C MRH1 AKR1 CCC2 GDA1 CAN1 SIT1 PIC2 WWM1 FET5 PMC1 OLE1 ZRT1 TPO2 VMR1 CTR2 ECM37 KHA1 OPI3 ZRT3 FRE2 MRS4 SMF3 HMX1 FRE1 YLR297W ECM7 FET3 FET4 PET8 CHS1 YOR291W FRE3 FRE5 YPR157W*	38	1545
Anchored to membrane	0031225	0.000455	*FIT1 YPS3 YLR194C FIT2 FIT3*	5	47
Cell wall	0005618	0.001027	*FIT1 YLR194C SRL1 FIT2 FIT3*	5	56
Anchored to plasma membrane	0046658	0.002778	*YPS3 YLR194C*	2	6
Vacuolar membrane	0005774	0.006072	*PMC1 TPO2 CTR2 ZRT3 SMF3 YLR297W GTR1*	7	157
Fungal-type cell wall	0009277	0.006108	*FIT1 PRY3 YLR194C SRL1 FIT2 FIT3*	6	119
Extracellular region	0005576	0.008642	*FIT1 PRY3 YLR194C SRL1 FIT2 FIT3*	6	128

DOWN-REGULATED GENES

GO Molecular Function					
**Category**	**GO ID**	**p-value**	**In Category from Cluster**	**k**	**f**
Heme binding	0020037	1.70E-06	*SDH4 CCP1 ERG5 CYB5 DAP1*	5	33
Iron ion binding	0005506	2.80E-06	*SDH4 LEU1 ACO2 CYT2 CCP1 ACO1 ERG5 CYB5*	8	145
4 iron, 4 sulfur cluster binding	0051539	0.000253	*LEU1 ACO2 ACO1*	3	20
Aconitate hydratase activity	0003994	0.000583	*ACO2 ACO1*	2	6
Iron-sulfur cluster binding	0051536	0.00136	*LEU1 ACO2 ACO1*	3	35
Lyase activity	0016829	0.001435	*LEU1 ACO2 CYT2 ACO1*	4	78
Transition metal ion binding	0046914	0.001721	*CYB5 DAP1*	2	10
Metal ion binding	0046872	0.004832	*SDH4 LEU1 ACO2 CYT2 CCP1 COX17 ACO1 ERG5 CYB5 QCR2*	10	625
C-22 sterol desaturase activity	0000249	0.006361	*ERG5*	1	1

GO Biological Process					
**Category**	**GO ID**	**p-value**	**In Category from Cluster**	**k**	**f**
Tricarboxylic acid cycle	0006099	0.00499	*SDH4 ACO2 ACO1*	3	55
Regulation of ergosterol biosynthetic process	0032443	0.006361	*DAP1*	1	1

GO Cellular Component					
**Category**	**GO ID**	**p-value**	**In Category from Cluster**	**k**	**f**
Mitochondrial intermembrane space	0005758	0.005249	*CYT2 CCP1 COX17*	3	56
3-isopropylmalate dehydratase complex	0009316	0.006361	*LEU1*	1	1
