# Supplementary material for: Novel insights into iron metabolism by integrating deletome and transcriptome analysis in an iron deficiency model of the yeast Saccharomyces cerevisiae
Source: BMC Genomics. 2009 Mar 25;10:130. doi: 10.1186/1471-2164-10-130 (PMC2669097; doi:10.1186/1471-2164-10-130)
Supplement: Additional file 10 — Summary of differentially-expressed genes from individual experiments in the dap1Δ, mrs4Δ and yhr045wΔ. Gene expression profiling was performed in the deletion mutants and wild type strain grown in YPD media. [file 1471-2164-10-130-S10.pdf]

Additional File 10: Summary of differentially-expressed genes from individual experiments in the *DAP1*, *MRS4* and *YHR045W* deletion mutants. The expression level is shown in logarithmic scale with base of two.

UP-REGULATED GENES IN *dap1Δ*

ORF	#1	#2	#3	#4
YAL002W	1.21	1.08	0.91	
YAL038W	0.92	1.33	0.91	
YAR068W	2.04	2.13	2.12	2.70
YBR093C	1.18	0.74	1.00	0.76
YBR118W	0.85	1.34	0.98	
YBR182C	1.43	1.27	1.06	
YCR012W	0.96	1.35	0.83	
YCR013C	1.27	1.28	0.84	
YDL228C	1.74	1.43	1.08	
YDR012W	1.18	1.13	0.90	
YDR077W	0.95	0.82	1.00	0.68
YER067W	0.60	0.69	0.66	0.84
YGL055W	1.36	0.91	1.14	1.02
YGR085C	1.18	1.18	0.93	
YGR139W	0.99		0.95	0.59

ORF	#1	#2	#3	#4
YHR214W	1.59	2.11	1.51	1.86
YHR214W-A	2.27	1.11	1.24	2.46
YIL131C		0.61	0.65	0.56
YIL146C	2.46	2.77	2.01	3.19
YIR042C	1.56	1.05	0.62	
YJL078C	0.79	0.62	0.73	
YJL138C	1.18	1.31	0.87	
YLR303W		3.38	3.21	5.04
YLR386W	1.98	1.27	0.67	
YLR390W	2.00	1.06	1.41	
YNL070W	1.16	1.33	0.85	
YOL098C	1.06	1.08	0.70	
YOL155C	1.71	1.58	1.42	1.56
YOR366W	0.98	1.06	1.08	
YPR102C	1.02	1.29	0.93	

DOWN-REGULATED GENES IN *dap1Δ*

ORF	#1	#2	#3	#4
YBL005W-B	-1.07	-0.70		-0.83
YBL034C	-0.94	-0.70	-0.94	-0.62
YBL101W-A	-0.72	-0.75		-0.89
YBL101W-B	-1.09	-0.79		-1.02
YBR012C	-1.29	-0.77	-0.68	-0.79
YBR073W	-0.70	-1.79	-1.14	-1.41
YBR083W	-1.24	-1.02	-1.34	-1.03
YBR157C	-1.91	-1.00	-1.32	-1.41
YBR158W		-1.16	-0.88	-1.01

ORF	#1	#2	#3	#4
YBR208C	-1.48	-0.95	-0.68	
YBR293W	-1.29	-1.08	-1.03	-0.68
YBR296C	-0.83	-0.78	-0.78	
YCL019W	-1.11		-0.93	-1.19
YCL020W		-1.26	-0.96	-0.64
YCL040W	-1.23	-1.17	-1.09	-0.60
YCLX07W	-2.39	-1.13	-1.35	-1.46
YCR097WB	-1.38	-0.83	-0.79	-0.79
YDR024W	-2.11	-1.03		-1.46

ORF	#1	#2	#3	#4
YDR128W		-0.84	-0.78	-0.83
YDR158W	-1.36	-0.83	-0.94	-1.02
YDR309C	-0.90	-0.77	-0.96	-0.55
YDR340W	-1.46		-0.60	-0.70
YEL065W	-0.89	-1.39	-0.78	-0.70
YER138C	-1.52	-1.36	-0.97	-1.40
YER160C	-1.39	-1.19	-0.94	-1.24
YER189W	-0.89	-0.78		-0.72
YFL026W	-4.93	-3.94	-4.11	-4.18
YFL027C	-0.77	-1.29	-0.96	-0.75
YFL059W	-1.65	-1.98	-1.62	-1.35
YFL061W	-0.88		-0.94	-0.72
YFL064C	-0.91	-0.92		-0.71
YFL-TYA	-0.95	-1.57	-1.12	-1.09
YFL-TYB	-1.18	-1.32	-0.98	-1.21
YGL032C	-4.32	-1.73	-1.91	-2.55
YGL073W	-1.79	-2.01		-1.87
YGL086W	-1.07	-1.39	-1.08	-1.11
YGL193C	-1.70	-0.90	-0.65	-0.84
YGL248W	-0.72	-1.30	-0.85	-0.59
YGR143W		-0.86	-0.96	-2.72
YHL027W	-1.14	-1.55	-0.96	-0.86
YHR056C	-1.32	-0.62	-1.16	
YHR185C	-0.65	-1.19	-1.00	-0.86
YHR214C-B	-1.36	-1.13	-1.09	-1.29
YIL015W	-4.38	-3.13	-3.53	-3.90
YJL130C		-0.97	-0.79	-0.94
YJR025C		-0.66	-0.62	-0.84
YJR027W	-1.35	-1.28	-0.86	-1.40
YJR029W	-1.00	-0.75	-0.91	-1.25
YJR086W	-2.14	-1.32	-1.08	-1.37
YJR159W	-1.33		-0.88	-0.98
YKL142W		-1.39	-1.01	-0.57
YKL178C	-0.88	-0.92	-0.98	-0.96
YLR136C	-1.55	-1.16	-1.50	-1.61
YLR256W	-0.86	-0.93	-0.85	-1.24
YLR265C	-2.66	-1.44	-1.57	-2.02

ORF	#1	#2	#3	#4
YLR343W	-1.79	-1.04	-1.08	-1.60
YLR346C	-1.34		-0.61	-1.00
YML039W	-1.03	-0.92	-0.72	-0.97
YML040W	-1.18	-1.72	-1.04	-1.63
YML045W	-1.63	-1.50	-1.22	-1.59
YML061C	-1.70	-1.61	-1.64	-1.58
YMR045C	-1.37	-1.42	-1.16	-1.21
YMR055C		-0.81	-1.04	-1.19
YMR173W	-1.16		-0.92	-1.16
YMR173W-A		-1.06	-1.27	-0.99
YMR287C	-0.84		-0.56	-0.71
YNL078W	-1.01	-0.74	-0.74	-0.60
YNL127W	-0.59	-0.86		-0.65
YNL145W	-5.36	-2.87	-2.80	-3.93
YNL155W	-0.83		-0.70	-0.93
YNL160W	-1.21		-1.34	-0.73
YNL333W	-1.52	-1.26	-1.77	-1.34
YNL334C	-2.14	-1.12	-1.47	-1.67
YOL158C	-1.17	-1.33	-1.43	-1.24
YOR072W		-0.64	-0.71	-0.69
YOR135C	-0.64	-0.65		-0.63
YOR136W	-0.63	-0.94	-0.69	-0.69
YOR212W	-2.96	-2.53	-2.52	-3.02
YOR284W	-0.81		-0.68	-0.64
YOR289W	-0.82	-1.12	-0.73	-0.57
YOR382W	-2.96	-1.96	-1.91	-2.47
YOR383C	-1.86	-1.25	-1.25	-1.50
YOR384W	-0.79	-0.97	-0.90	-0.57
YPL057C	-1.07	-1.14	-0.91	-0.89
YPL058C	-1.47	-0.65		-0.97
YPL107W		-1.02	-1.09	-0.89
YPL170W	-3.75	-2.30	-2.90	-3.26
YPL256C	-1.03	-0.68	-0.58	-0.52
YPR027C	-0.99	-1.28	-0.84	-1.25
YPR122W	-1.78	-1.73	-1.34	-0.98

UP-REGULATED GENES IN *mrs4Δ*

ORF	#1	#2	#3
YAL067C	1.43		1.82
YBL034C	1.66		1.66
YBR024W	1.19		1.36
YBR046C	1.70	2.38	
YBR047W	2.93	2.42	
YBR054W	3.62	3.06	
YBR126C	2.03		2.37
YCL040W	3.95	3.25	3.36
YCL042W	3.70	1.91	
YCL049C	1.75	1.51	
YCL064C		1.36	1.43
YCR005C		1.59	1.73
YCR012W		1.40	1.74
YCR021C	4.17	4.38	
YDL021W	2.84	2.69	3.91
YDL022W	1.63	1.55	
YDL023C	1.46	2.60	
YDL048C	2.53		2.17
YDL124W	2.59	2.39	
YDL169C	2.86	3.64	
YDL232W		1.37	1.43
YDL234C	1.40	1.90	1.85
YDR043C	1.37	1.42	
YDR055W	2.65	2.34	
YDR059C	1.63	1.42	2.14
YDR074W	1.69		1.30
YDR171W	2.65	1.88	2.89
YDR247W	1.56	1.92	
YDR258C	1.99		1.72
YDR270W	1.56	1.64	1.63
YDR277C	1.92		1.34
YDR342C	2.95	2.53	2.62
YDR391C	1.57	3.10	
YEL011W	3.41		3.20
YEL060C	1.53		2.05
YER024W	1.73	2.46	
YER053C	4.88	2.75	
YER062C	2.92	2.09	

ORF	#1	#2	#3
YER067W	5.21	4.72	
YER098W	1.27		2.35
YER175C	2.67	2.49	
YFL014W	3.95		3.26
YFL020C	1.61	3.32	1.91
YFL026W	1.90		1.68
YFR015C	3.22		3.07
YFR053C	1.99	3.15	3.32
YGL032C	1.57		2.80
YGL037C	2.77	1.51	
YGL064C	0.98	4.39	
YGL073W	3.85	2.87	4.01
YGL248W	1.76		2.02
YGR008C	3.44		3.56
YGR138C	1.84	1.75	
YHL027W	3.26		2.84
YHR016C	1.89	2.50	2.17
YHR044C	1.59		1.73
YHR053C	3.52	3.18	3.32
YHR179W		1.38	1.59
YIL136W	1.39		1.50
YIR017C	1.94		1.66
YIR038C	1.62	1.83	
YJL052W	2.18	1.43	1.87
YJL079C	2.29	1.80	2.88
YJL116C	1.95		1.54
YJR148W	1.96	2.63	1.81
YKL026C	1.70	1.50	
YKL062W	2.63		2.07
YKL103C	2.10	1.83	2.79
YKL109W	2.16	2.28	1.74
YKL142W	1.95	2.46	1.84
YKL150W	1.34		1.62
YKL178C	2.92		2.82
YKL220C	1.33		1.79
YKR058W	1.78		2.22
YKR075C	1.64	2.19	
YKR091W	1.16	1.57	

ORF	#1	#2	#3
YKR093W	1.23	2.39	1.34
YKR098C	1.76		1.56
YLL026W	1.49		1.83
YLL056C	2.56	2.57	
YLR136C	2.69	2.35	2.84
YLR178C	3.05		2.58
YLR258W	2.18		2.12
YLR270W	1.28	1.88	
YLR286C		1.74	1.54
YLR297W	1.66	1.89	
YLR327C	4.63	4.77	
YLR345W	1.14	1.97	
YLR346C	3.34	2.10	
YML121W		1.40	1.35
YMR008C		1.52	1.79
YMR009W	1.29	2.00	
YMR011W		1.41	1.49
YMR020W	1.31		1.47
YMR058W		1.71	1.75
YMR105C	3.49	3.11	3.65
YMR173W	2.14	2.23	
YMR173W-A	1.68	2.49	
YMR175W	1.10	1.53	
YMR181C	2.28	2.34	
YMR251W-A	2.48	1.98	
YMR315W	1.48	1.47	
YNL015W	1.92		1.96
YNL037C	2.62	3.55	

ORF	#1	#2	#3
YNL144C	1.761	1.76	
YNL145W	4.491	1.65	3.95
YNL160W	5.38	5.19	5.76
YNL200C	2.32	2.35	
YNL208W	1.60	2.24	
YNR001C	2.28	3.03	2.71
YOL059W	2.23		2.10
YOL158C	2.05	2.78	
YOL163W	2.34	1.39	
YOR036W	1.43	1.42	
YOR120W	1.37		1.64
YOR135C	2.60		2.00
YOR153W	1.74	2.04	2.43
YOR215C	1.20	2.32	
YOR237W	1.66		1.89
YOR285W	1.91	1.32	
YOR289W	3.78	1.70	
YOR338W	4.00	2.84	
YOR347C	1.31	1.69	
YOR360C		1.44	1.41
YOR381W	1.54	1.66	
YPL004C	1.47	2.18	
YPL087W	1.51	1.77	
YPL154C	2.01		1.98
YPL171C	1.15		1.68
YPL250C	2.47	1.94	
YPR036W		1.62	1.43
YPR160W	3.64		4.33

DOWN-REGULATED GENES IN *mrs4Δ*

ORF	#1	#2	#3
YAR071W	-1.64	-1.72	
YBR021W	-1.28		-1.36
YBR084W	-1.12		-1.46
YBR093C	-1.81		-2.19
YBR296C	-1.30	-1.36	
YCR058C	-2.16		-1.47

ORF	#1	#2	#3
YDR135C	-1.77		-1.95
YDR212W	-1.72		-1.92
YDR234W	-1.59	-1.43	-1.85
YDR281C	-1.10	-1.78	
YEL033W	-1.57	-2.42	
YEL034W	-1.72	-1.52	

ORF	#1	#2	#3
YER001W	-1.60	-2.51	
YER156C	-1.75	-1.33	
YGL009C	-3.50	-2.22	-2.63
YGL077C	-1.28		-1.43
YGR109C	-1.77		-2.00
YGR123C	-1.24		-1.72
YGR164W	-1.04	-2.06	
YGR286C	-1.54		-1.61
YHR007C	-0.93		-1.48
YHR208W	-1.88	-2.02	
YHR215W	-1.76	-1.49	-2.17

ORF	#1	#2	#3
YJL033W		-1.40	-1.44
YKL106W	-1.35		-1.72
YKR052C	-2.58	-2.39	-2.88
YLR083C	-1.21		-1.41
YLR355C	-1.15		-2.76
YLR398C	-1.67		-2.02
YMR230W	-1.12	-1.41	
YMR260C	-1.18		-2.10
YNL111C	-1.61	-1.59	-1.67
YNL114C	-1.06	-1.55	
YPL060W	-1.35	-1.77	

UP-REGULATED GENES IN *ydr045wΔ*

ORF	#1	#2
YAL067C	1.19	1.19
YAL068C	1.53	1.27
YAR020C	1.74	1.53
YBL005W	1.87	1.90
YBL034C	1.59	1.45
YBL034C	1.19	1.14
YBL043W	2.23	2.61
YBL049W	1.43	1.41
YBL078C	1.19	1.11
YBR005W	1.01	1.02
YBR046C	1.01	1.17
YBR046C	1.24	
YBR047W	2.03	2.01
YBR047W	2.26	2.16
YBR066C	0.88	0.90
YBR105C	2.31	2.15
YBR126C	1.20	1.16
YBR149W	1.25	1.12
YBR183W	1.51	1.27
YBR269C	1.08	0.90
YCL019W	0.99	0.95
YCL040W	1.38	1.76
YCL049C	1.00	0.94
YCL061C	0.99	1.04
YCR005C	1.67	1.76
YDL021W	2.35	2.25
YDL022W	1.07	0.88
YDL023C	1.33	1.41
YDL038C	1.48	1.22
YDL048C	1.65	1.52
YDL110C	0.85	0.77
YDL124W	1.67	1.54
YDL234C	1.31	1.16
YDR011W	1.26	1.20
YDR032C	1.13	1.04
YDR055W	1.22	1.28
YDR058C	1.01	0.93
YDR072C	0.92	0.86

ORF	#1	#2
YDR074W	1.16	0.83
YDR158W	0.92	0.88
YDR171W	1.30	1.25
YDR258C	1.31	1.38
YDR264C	0.80	1.38
YDR271C	1.77	1.49
YDR516C	0.88	1.02
YEL049W	1.32	1.23
YEL065W	2.08	1.86
YEL066W	1.32	1.30
YEL067C	0.99	1.19
YEL071W	1.26	1.10
YER024W	1.92	1.97
YER035W	1.56	1.67
YER038C	1.83	1.55
YER079W	1.77	1.63
YER124C	1.21	1.32
YER138C	0.84	0.79
YER160C	0.85	0.81
YER175C	2.09	2.28
YER178W	0.87	0.86
YFL014W	4.24	4.10
YFL020C	1.34	1.46
YFL041W	1.53	1.46
YFL067W	1.12	1.07
YFR017C	1.81	1.64
YFR053C	1.76	1.75
YGL037C	1.31	1.28
YGL073W	3.68	3.74
YGL086W	0.77	0.87
YGL156W	1.86	1.45
YGL157W	1.65	1.72
YGL260W	1.01	0.92
YGR008C	1.44	1.48
YGR017W	0.94	1.00
YGR131W	1.38	1.20
YGR141W	0.84	0.84
YGR146C	0.97	0.96

ORF	#1	#2
YGR146C	1.02	1.10
YGR161C	1.84	1.82
YGR161C	2.01	2.10
YGR175C	1.20	1.03
YGR213C	1.36	1.40
YGR239C	1.23	1.08
YGR244C	0.79	0.77
YGR257C	1.01	0.89
YHL027W	3.10	3.06
YHL035C	1.46	1.43
YHR006W	1.40	1.33
YHR016C	0.87	1.06
YHR038W	0.79	0.87
YHR046C	1.32	1.91
YHR047C	1.51	1.57
YHR053C	1.54	1.92
YHR070W	1.29	1.28
YHR113W	1.59	1.65
YHR138C	1.17	1.24
YHR140W	0.94	0.89
YHR179W	1.06	0.95
YHR214C-B	0.90	0.92
YIL015C-A	1.03	1.02
YIL070C	0.93	0.88
YIL095W	1.54	1.64
YIR018W	1.54	1.20
YIR038C	1.36	1.42
YJL052W	0.89	1.03
YJL066C	0.98	0.93
YJL067W	1.01	0.95
YJL079C	1.41	1.34
YJL088W	1.08	1.27
YJL116C	1.53	1.62
YJR148W	1.62	1.62
YJR150C	2.15	2.22
YKL008C	1.27	1.22
YKL026C	1.15	1.05
YKL026C		1.01
YKL071W	1.12	1.08
YKL085W	1.09	0.96

ORF	#1	#2
YKL103C	1.10	1.19
YKL109W	1.45	1.55
YKL142W	1.64	1.65
YKL150W	0.96	0.94
YKL151C	1.59	1.42
YKR053C	1.21	1.03
YKR076W	1.19	1.58
YKR093W	1.20	1.17
YLL019C	1.34	1.18
YLL025W	1.52	1.23
YLL056C	1.95	2.07
YLR037C	1.60	1.71
YLR056W	1.61	1.51
YLR089C	1.04	0.88
YLR099C	1.33	1.38
YLR120C	0.83	0.99
YLR136C	3.06	3.00
YLR152C	1.16	1.15
YLR178C	2.14	2.24
YLR194C	1.44	1.48
YLR270W	1.36	1.25
YLR286C	1.50	1.30
YLR297W	1.47	1.38
YLR303W	1.44	1.48
YLR304C	2.00	2.19
YLR327C	3.27	3.26
YLR327C	2.71	3.14
YLR345W	1.05	0.98
YLR346C	3.63	3.64
YLR346C	3.29	2.8
YML008C	0.98	0.95
YML039W	0.81	0.84
YML047C	1.00	0.91
YMR008C	1.02	0.97
YMR041C	0.83	0.86
YMR045C	0.81	0.85
YMR095C	4.33	4.66
YMR096W	3.22	3.17
YMR102C	2.56	2.69
YMR105C	1.88	1.87

ORF	#1	#2
YMR134W	0.92	0.82
YMR145C	1.33	1.22
YMR173W	1.64	1.71
YMR173W-A	1.79	1.68
YMR195W	1.48	1.68
YMR244C-A	1.42	1.34
YMR245W	1.07	1.00
YMR251W-A	1.25	1.26
YMR291W	1.16	1.14
YMR316C-A	1.40	1.4
YMR316W	1.49	1.53
YNL015W	1.32	1.23
YNL040W	0.84	1.00
YNL055C	1.09	1.05
YNL160W	3.92	3.79
YNL192W	0.88	0.84
YNL200C	1.20	1.35
YNL208W	1.26	1.18
YNL234W	1.05	0.98
YNL242W	0.97	1.10
YNR001C	1.78	1.66
YOL053C-A	2.05	1.93
YOL118C	1.07	0.96
YOL136C	0.92	0.94
YOL150C	1.79	1.76
YOL151W	1.86	1.89
YOL158C	1.97	1.99
YOL161C	1.20	1.23
YOR036W	0.78	0.82
YOR049C	3.31	3.71
YOR135C	3.05	2.96

ORF	#1	#2
YOR136W	2.78	2.7
YOR137C	0.99	0.91
YOR153W	2.85	2.8
YOR173W	1.33	1.29
YOR185C	1.30	0.97
YOR237W	3.09	3.22
YOR289W	2.00	2.61
YOR303W	1.44	1.32
YOR306C	0.85	1.03
YOR338W	3.68	3.78
YOR344C	1.10	1.14
YOR347C	1.39	1.20
YOR360C	0.82	0.84
YOR363C	1.10	1.30
YOR382W	5.30	3.22
yor383c	4.92	5.10
YOR383C	4.55	4.45
YOR384W	1.53	2.21
YOR394W	1.83	1.78
YPL059W	1.09	1.00
YPL086C	1.30	1.33
YPL087W	1.16	1.07
YPL087W	1.31	1.39
YPL154C	1.64	1.58
YPL159C	0.91	0.95
YPL250C	1.58	1.69
YPL272C	2.33	2.31
YPL282C	1.06	1.73
YPR002W	2.30	2.30
YPR149W	0.98	0.89
YPR156C	0.82	0.78

DOWN-REGULATED GENES IN *ydr045wΔ*

ORF	#1	#2
YAR070C	-1.08	-1.60
YBR021W	-0.90	-1.06
YBR092C	-1.26	-1.29

ORF	#1	#2
YBR093C	-1.39	-1.46
YBR108W	-1.05	-1.75
YBR244W	-1.15	-1.18

ORF	#1	#2
YCR102C	-1.13	-1.3
YDL184C	-1.32	-1.37
YDL241W	-1.24	-1.12
YDR111C	-0.98	-0.87
YDR216W	-0.85	-0.92
YDR382W	-0.88	-0.84
YDR399W	-1.01	-1.08
YEL033W	-1.06	-1.14
YER060W-A	-1.29	-1.40
YER174C	-0.96	-0.90
YGL009C	-1.24	-1.32
YGL010W	-0.80	-0.79
YGL234W	-0.78	-0.78
YGL241W	-2.88	-3.29
YGR034W	-1.23	-1.40
YGR043C	-3.15	-2.71
YGR109C	-1.33	-1.28
YGR109C	-1.07	-1.05
YGR164W	-1.12	-1.50
YGR164W	-1.72	-1.88
YGR270W	-1.36	-1.79
YHL001W	-0.93	-0.88
YHL037C	-1.50	-1.64
YHR010W	-0.93	-0.94
YHR021C	-1.05	-1.07
YHR051W	-0.88	-0.83
YHR096C	-2.98	-3.44
YIL025C	-2.06	-2.96
YIL146C	-1.54	-1.52
YIR014W	-1.15	-1.15
YJL136C	-0.99	-1.04
YJL188C	-0.91	-0.96
YJL189W	-1.03	-1.01

ORF	#1	#2
YJL200C	-1.00	-0.92
YJR094W-A	-1.03	-1.05
YKL096W	-0.87	-1.06
YKR057W	-1.10	-1.14
YLL014W	-0.84	-0.82
YLR167W	-1.04	-1.15
YLR264W	-1.13	-1.00
YLR325C	-1.27	-1.34
YLR344W	-1.13	-1.32
YLR359W	-0.98	-0.96
YLR359W	-1.07	-1.14
YLR449W	-0.86	-0.89
YML024W	-0.87	-0.93
YML026C	-1.08	-1.01
YMR230W	-1.29	-1.24
YMR321C	-0.94	-1.03
YNL109W	-1.2	-1.18
YNL303W	-1.35	-1.42
YOL109W	-0.82	-0.90
YOL155C	-1.04	-1.08
YOR096W	-1.49	-1.09
YOR167C	-1.37	-1.42
YOR271C	-0.85	-0.89
YOR366W	-1.88	-1.95
YPL143W	-1.00	-1.14
YPL263C	-0.82	-0.80
YPL274W	-1.44	-1.51
YPR044C	-0.97	-0.90
YPR065W	-1.48	-1.64
YPR077C	-2.10	-1.70
YPR167C	-2.08	-2.55
