# Supplementary material for: Novel insights into iron metabolism by integrating deletome and transcriptome analysis in an iron deficiency model of the yeast Saccharomyces cerevisiae
Source: BMC Genomics. 2009 Mar 25;10:130. doi: 10.1186/1471-2164-10-130 (PMC2669097; doi:10.1186/1471-2164-10-130)
Supplement: Additional file 11 — List of differentially-expressed genes in cti6Δ. Gene expression profiling was performed in cti6Δ and wild type strain grown in YPD media. Cti6p relieves transcriptional repression by binding to the Cyc8p-Tup1p corepressor and recruiting the SAGA complex to repressed promoters. [file 1471-2164-10-130-S11.pdf]

Additional File 11: List of differentially-expressed genes in the *cti6Δ* (*ypl181wΔ*) mutant. Differentially-expressed genes in one experiment are shown with expression levels in logarithmic scale with base of two. The GO biological process from FunSpec was used to categorize genes according to their cellular functions.

UP-REGULATED GENES

Gene ID	Gene Name	Average expression	Function/ Activity
Carbohydrate metabolism and energy metabolism (12)			
YAL038W	*CDC19*	1.45	Pyruvate kinase
YBL082C	*RHK1*	0.62	Mannosyltransferase involved in N-glycosylation
YCR012W	*PGK1*	1.36	Phosphoglycerate kinase
YDL066W	*IDP1*	0.76	Isocitrate dehydrogenase
YGR192C	*TDH3*	1.36	Glyceraldehyde-3-phosphate dehydrogenase 3
YGR240C	*PFK1*	1.48	Phosphofructokinase alpha subunit
YGR254W	*ENO1*	1.30	Enolase 1
YJL137C	*GLG2*	0.62	Self-glucosylating initiator of glycogen synthesis
YKL085W	*MDH1*	0.84	Malate dehydrogenase, mitochondrial
YLR304C	*ACO1*	0.75	Aconitase
YML110C	*COQ5*	0.71	Mitochondrial C-methyltransferase
YOL059W	*GPD2*	1.04	Glycerol-3-phosphate dehydrogenase
Metal homeostasis (4)			
YBR295W	*PCA1*	0.88	P-type copper-transporting ATPase
YGL166W	*ACE1*	2.14	Copper-dependent transcription factor
YLR136C	*TIS11*	1.48	Protein of the inducible CCCH zinc finger family
YMR319C	*FET4*	0.78	Low-affinity Fe(II) transport protein
Nucleotide metabolism (14)			
YBL002W	*HTB2*	1.30	Histone H2B
YBL088C	*TEL1*	1.08	Phosphatidylinositol 3-kinase
YBR010W	*HHT1*	1.24	Histone H3
YBR253W	*SRB6*	0.62	Component of the RNA polymerase II holoenzyme
YDL160C	*DHH1*	0.84	Putative RNA helicase
YDR224C	*HTB1*	1.40	Histone H2B
YDR225W	*HTA1*	1.22	Histone H2A
YGL073W	*HSF1*	1.03	Heat shock transcription factor
YHL027W	*RIM101*	1.03	Transcription factor for sporulation related genes
YIL066C	*RNR3*	0.87	Ribonucleotide reductase
YLR398C	*SKI2*	0.72	Antiviral protein and probable helicase
YMR283C	*RIT1*	2.29	Initiator tRNA phosphoribosyl-transferase
YPL089C	*RLM1*	1.62	Transcription factor of the MADS box family
YPR018W	*RLF2*	0.64	Chromatin assembly complex subunit 1
Others (4)			
YAL002W	*VPS8*	1.22	Protein involved in vacuolar sorting
YBR222C	*PCS60*	0.78	Peroxisomal AMP-binding protein
YGL205W	*POX1*	1.28	Acyl-CoA oxidase

Gene ID	Gene Name	Average expression	Function/ Activity
Others			
YOR370C	*MRS6*	0.70	Rab geranylgeranyltransferase regulatory component and rab guanine nucleotide dissociation inhibitor
Protein metabolism (25)			
YBR118W	*TEF2*	1.46	Translation elongation factor EF-1alpha
YBR181C	*RPS6B*	1.20	Ribosomal protein S6
YBR189W	*RPS9B*	1.39	Ribosomal protein S9
YBR191W	*RPL21A*	1.38	Ribosomal protein L21
YDL075W	*RPL31A*	1.31	Ribosomal protein L31
YDL130W	*RPP1B*	1.30	Acidic ribosomal protein P1B
YGL031C	*RPL24A*	1.39	Ribosomal protein L24A
YGL135W	*RPL1B*	1.51	Large subunit ribosomal protein L1
YGL189C	*RPS26A*	1.10	Ribosomal protein S26
YGR085C	*RPL11B*	1.50	Ribosomal protein L11
YGR148C	*RPL24B*	1.43	Ribosomal protein L24B
YHL033C	*RPL8A*	1.46	Ribosomal protein L8
YJL138C	*TIF2*	1.37	Translation initiation factor 4A
YJR123W	*RPS5*	1.04	Ribosomal protein Rps5p
YJR145C	*RPS4A*	1.29	Ribosomal protein S4
YKL006W	*RPL14A*	1.10	Ribosomal protein L14
YKR059W	*TIF1*	1.44	Translation initiation factor 4A
YLR075W	*RPL10*	1.42	Ribosomal protein L10
YLR264W	*RPS28B*	1.19	Ribosomal protein S28
YLR303W	*MET17*	4.51	O-Acetylhomoserine sulfhydrylase
YLR333C	*RPS25B*	1.25	Ribosomal protein S25B
YNL178W	*RPS3*	1.31	Ribosomal protein S3
YNL244C	*SUI1*	1.23	Translation initiation factor eIF3
YPL037C	*EGD1*	1.45	Beta subunit of the nascent polypeptide-associated complex
YPR132W	*RPS23B*	1.35	Ribosomal protein S23
Transporters (4)			
YDR011W	*SNQ2*	0.78	Drug-efflux pump involved in resistance to multiple drugs
YDR039C	*ENA2*	0.59	Member of the Na[+]-transporting P-type ATPases family
YNL070W	*TOM7*	1.24	Subunit of the translocase involved in mitochondrial protein import
YOR153W	*PDR5*	0.60	Drug-efflux pump
Functionally unknown genes (3)			
YDR077W	*SED1*	1.02	Abundant cell surface glycoprotein
YKL095W	*YJU2*	0.66	Protein possibly involved in pre-mRNA splicing
YOR135C	*YOR135C*	1.03	Protein possibly required for full induction of IME1 during early meiosis

DOWN-REGULATED GENES

Gene ID	Gene Name	Average expression	Function/ Activity
Carbohydrate metabolism (12)			
YBL015W	*ACH1*	-0.73	Acetyl-CoA hydrolase
YBR019C	*GAL10*	-0.95	UDP-glucose 4-epimerase
YBR297W	*MAL33*	-1.11	Maltose fermentation regulatory protein
YDL021W	*GPM2*	-1.60	Phosphoglycerate mutase
YER178W	*PDA1*	-0.82	Pyruvate dehydrogenase complex
YFL018C	*LPD1*	-0.79	Dihydrolipoamide dehydrogenase
YFR015C	*GSY1*	-0.83	Glycogen synthetase isoform 1
YGR143W	*SKN1*	-1.14	Glucan synthase
YHR044C	*DOG1*	-1.08	2-Deoxyglucose-6-phosphate phosphatase
YJR158W	*HXT16*	-1.02	Member of the hexose transporter family of the major facilitator superfamily (MFS)
YJR159W	*SOR1*	-2.63	Sorbitol dehydrogenase
YLR273C	*PIG1*	-1.01	Involved in glycogen synthesis
Cell growth and maintenance (23)			
YBL016W	*FUS3*	-1.90	Serine/threonine protein kinase
YBR073W	*RDH54*	-1.67	Required for mitotic diploid-specific recombination and repair and for meiosis
YDL240W	*LRG1*	-0.68	GTPase-activating protein of the rho/rac family
YFL003C	*MSH4*	-1.24	Meiosis-specific protein
YFL026W	*STE2*	-3.70	Pheromone alpha-factor G protein-coupled receptor
YGL032C	*AGA2*	-4.74	Binding subunit of a-agglutinin
YGL086W	*MAD1*	-1.32	Involved in spindle-assembly checkpoint
YGR044C	*RME1*	-2.82	Transcription factor that represses meiosis in non-a/alpha cells
YHR084W	*STE12*	-0.77	Transcription factor that binds to pheromone response element
YHR086W	*NAM8*	-1.04	U1 snRNA-associated protein
YIL015W	*BAR1*	-5.85	Secreted pepsin-like protease that degrades alpha-factor
YIL140W	*AXL2*	-0.80	Glycoprotein localized at site of bud emergence
YJR086W	*STE18*	-1.30	Mediates signal transduction by pheromones during mating
YKL178C	*STE3*	-1.46	Pheromone a-factor G protein-coupled receptor
YKL209C	*STE6*	-1.26	Membrane transporter responsible for export of a factor mating pheromone
YLR452C	*SST2*	-4.07	Negatively regulates the mating pheromone response pathway
YMR055C	*BUB2*	-1.28	Checkpoint protein required for cell cycle arrest
YNL145W	*MFA2*	-6.66	a-Factor mating pheromone precursor
YNL289W	*PCL1*	-0.82	G1/S-specific cyclin
YNR044W	*AGA1*	-1.22	Anchor subunit of a-agglutinin
YOR212W	*STE4*	-3.79	Mediates signal transduction by pheromones
YPL121C	*MEI5*	-0.80	Meiotic protein required for synapsis and meiotic recombination
YPL256C	*CLN2*	-0.95	G1/S-specific cyclin
Nucleotide metabolism (13)			
YBR083W	*TEC1*	-2.31	Transcriptional activator
YCR097WB	*HMRA1*	-0.85	Homeodomain regulatory protein
YER172C	*BRR2*	-1.26	RNA helicase-related protein
YGL248W	*PDE1*	-0.69	3',5'-Cyclic-nucleotide phosphodiesterase
YGR091W	*PRP31*	-0.62	Required for pre-mRNA splicing

Gene ID	Gene Name	Average expression	Function/ Activity
Nucleotide metabolism			
YIR017C	*MET28*	-0.96	Transcriptional activator regulating sulfur amino acid metabolism
YJL130C	*URA2*	-1.44	Multifunctional protein of pyrimidine biosynthesis pathway
YKL062W	*MSN4*	-0.92	Transcriptional activator for genes regulated through Snf1p,
YLR256W	*HAP1*	-1.17	Transcription factor with heme-dependent DNA-binding activity
YML061C	*PIF1*	-1.72	DNA-dependent ATPase and 5'-3' DNA helicase
YNL261W	*ORC5*	-0.67	Origin recognition complex
YOL004W	*SIN3*	-1.05	Component of histone deacetylase B
YPR047W	*MSF1*	-0.62	Phenylalanyl-tRNA synthetase
Others (31)			
YDR058C	*TGL2*	-1.09	Triglyceride lipase
YDR127W	*ARO1*	-1.24	Arom pentafunctional enzyme
YDR212W	*TCP1*	-1.13	Component of chaperonin-containing T-complex
YDR538W	*PAD1*	-0.98	Phenylacrylic acid decarboxylase
YER120W	*SCS2*	-0.80	Suppressor of choline synthesis
YER155C	*BEM2*	-1.08	GTPase-activating (GAP) protein involved in cell organization
YFL058W	*THI5*	-1.97	Involved in the production of thiamine precursor
YGL252C	*RTG2*	-0.67	Involved in inter-organelle communication
YGR144W	*THI4*	-0.92	Thiamine-repressed protein
YGR286C	*BIO2*	-0.67	Biotin synthetase
YHL003C	*LAG1*	-0.60	Required for ceramide synthesis and ER-to-Golgi transport of GPI-anchored proteins
YHR215W	*PHO12*	-1.36	Acid phosphatase
YIL114C	*POR2*	-0.86	Outer mitochondrial membrane porin
YIR028W	*DAL4*	-2.15	Allantoin permease
YJL165C	*HAL5*	-0.71	Serine/threonine protein kinase involved in salt and pH tolerance
YJR121W	*ATP2*	-1.30	Beta subunit of the F1 subunit of ATP synthase
YLL001W	*DNM1*	-1.08	Dynamin-related protein
YLR078C	*BOS1*	-0.74	Synaptobrevin homolog
YLR113W	*HOG1*	-0.67	MAP kinase
YLR248W	*RCK2*	-0.70	Calcium/calmodulin-dependent serine/threonine protein kinase
YLR292C	*SEC72*	-0.61	Component of ER protein-translocation subcomplex
YLR393W	*ATP10*	-1.05	Protein required for F1-F0 ATP synthase assembly
YMR008C	*PLB1*	-1.03	Phospholipase B
YMR056C	*AAC1*	-0.73	ADP/ATP carrier protein of the mitochondrial carrier family (MCF) of membrane transporters
YMR169C	*ALD3*	-1.12	Stress inducible aldehyde dehydrogenase
YNL093W	*YPT53*	-0.87	Involved in endocytosis and transport of proteins to the vacuole
YNL329C	*PEX6*	-0.70	Peroxisomal biogenesis protein
YNR015W	*SMM1*	-0.76	Member of conserved dihydrouridine synthase family
YNR033W	*ABZ1*	-0.92	Para-aminobenzoate synthase
YPL057C	*SUR1*	-0.80	Required for the synthesis of mannosylated sphingolipids
YPL177C	*CUP9*	-0.80	Involved in copper homeostasis
Protein metabolism (3)			
YDR158W	*HOM2*	-1.39	Aspartate-semialdehyde dehydrogenase
YHR010W	*RPL27A*	-0.76	Ribosomal protein L27

Gene ID	Gene Name	Average expression	Function/ Activity
Protein metabolism (cont'd)			
YKL210W	*UBA1*	-1.04	Ubiquitin-activating (E1) enzyme
Functionally unknown genes (10)			
YAL067C	*SEO1*	-0.78	Member of the allantoate permease family of the major facilitator superfamily (MFS)
YCR097WA	*YCR097WA*	-0.72	Unknown
YDL048C	*STP4*	-0.59	Protein with strong similarity to Stp1p
YEL049W	*PAU2*	-0.95	Protein with strong similarity to S. cerevisiae Yal068p
YER101C	*AST2*	-0.66	Protein involved in sorting of membrane proteins
YFL020C	*PAU5*	-0.79	Member of the seripauperin (PAU) family
YIR030C	*DCG1*	-2.70	Protein possibly involved in cell wall structure or biosynthesis
YLL019C	*KNS1*	-0.77	Serine/threonine protein kinase with unknown role
YOR156C	*NFI1*	-0.71	Chromatin protein
YPL058C	*PDR12*	-0.78	Member of the ATP-binding cassette (ABC) superfamily of membrane transporters
