# Supplementary material for: Novel insights into iron metabolism by integrating deletome and transcriptome analysis in an iron deficiency model of the yeast Saccharomyces cerevisiae
Source: BMC Genomics. 2009 Mar 25;10:130. doi: 10.1186/1471-2164-10-130 (PMC2669097; doi:10.1186/1471-2164-10-130)
Supplement: Additional file 12 — List of differentially-expressed genes in the dap1Δ. Gene expression profiling was performed in dap1Δ and wild type strain grown in YPD media. Dap1p is a heme-binding protein involved in regulation of cytochrome P450 protein Erg11p, related to mammalian membrane progesterone receptors. [file 1471-2164-10-130-S12.pdf]

Additional File 12: List of differentially-expressed genes in the *dap1Δ* mutant. Genes that were up- or down-regulated in at least three out of four independent experiments are listed and categorized according to their cellular functions using the GO biological process from Funspec. The average expression level is shown in logarithmic scale with base of two.

UP-REGULATED GENES

Gene ID	Gene Name	Average expression	Function / Activity
Cell growth and maintenance (3)			
YIL131C	*FKH1*	0.30	Homolog of Drosophila forkhead protein, involved in transcriptional silencing, cell morphology and cell cycle
YIL146C	*ECM37*	2.61	Protein possibly involved in cell wall structure or biosynthesis
YLR390W	*ECM19*	1.49	Protein possibly involved in cell wall structure or biosynthesis
Others (8)			
YAL002W	*VPS8*	1.07	Protein involved in vacuolar sorting
YAL038W	*CDC19*	1.05	Pyruvate kinase, catalyzes final step in glycolysis
YBR093C	*PHO5*	0.92	Acid phosphatase
YBR182C	*SMP1*	1.25	Transcription factor and target of the Hog1p (MAPK) high osmolarity signal transduction pathway
YCR012W	*PGK1*	1.05	Phosphoglycerate kinase in glycolysis
YGL055W	*OLE1*	1.11	Stearoyl-CoA desaturase, required for synthesis of unsaturated fatty acids
YLR386W	*VAC14*	1.31	Protein involved in Fab1p-dependent phosphatidylinositol(3,5) bisphosphate synthesis, vacuolar size and acidity, and transport of Ppn5p to the multivesicular body
YNL070W	*TOM7*	1.11	Small subunit of the translocase involved in mitochondrial protein import
Protein metabolism (6)			
YBR118W	*TEF2*	1.06	Translation elongation factor EF-1alpha
YDR012W	*RPL4B*	1.07	Ribosomal protein L4
YGR085C	*RPL11B*	1.11	Ribosomal protein L11
YJL138C	*TIF2*	1.12	Translation initiation factor 4A
YLR303W	*MET17*	3.88	O-Acetylhomoserine sulfhydrylase
YPR102C	*RPL11A*	1.08	Ribosomal protein L11
Functionally unknown genes (13)			
YAR068W	*YAR068W*	2.25	Unknown
YCR013C	*YCR013C*	1.13	Unknown
YDL228C	*YDL228C*	1.42	Unknown

Gene ID	Gene Name	Average expression	Function / Activity
Functionally unknown genes (cont'd)			
YDR077W	*SED1*	0.86	Abundant cell surface glycoprotein that may contribute to cell wall integrity and stress resistance
YER067W	*YER067W*	0.70	Unknown
YGR139W	*YGR139W*	0.84	Unknown
YHR214W	*YHR214W*	1.83	Unknown
YHR214W-A	*YHR214W-A*	2.12	Unknown
YIR042C	*YIR042C*	1.26	Member of the acetyltransferase (GNAT) family
YJL078C	*PRY3*	0.71	Protein with similarity to plant pathogenesis-related proteins
YOL098C	*YOL098C*	0.95	Member of the insulinase (peptidase M16) family
YOL155C	*YOL155C*	1.57	Protein with similarity to S. cerevisiae glucan 1,4-alpha-glucosidase
YOR366W	*YOR366W*	1.04	Unknown

DOWN-REGULATED GENES

Gene ID	Gene Name	Average expression	Function / Activity
Cell growth and maintenance (17)			
YDR309C	*GIC2*	-0.80	Putative effector of Cdc42p, important for bud emergence
YFL026W	*STE2*	-4.29	Pheromone alpha-factor G protein-coupled receptor
YGL032C	*AGA2*	-2.63	Binding subunit of a-agglutinin
YGL086W	*MAD1*	-1.16	Protein involved in spindle-assembly checkpoint, required for cell cycle delay in response to impaired kinetochore function
YGR143W	*SKN1*	-1.51	Glucan synthase subunit involved in synthesis of beta-1,6-glucan
YHR056C	*RSC30*	-0.81	Component of the abundant RSC chromatin remodeling complex
YHR185C	*PFS1*	-0.93	Protein required for prospore membrane formation during sporulation
YIL015W	*BAR1*	-2.89	Secreted pepsin-like protease that degrades alpha-factor
YJR086W	*STE18*	-1.48	Gamma subunit of the guanine nucleotide-binding protein that mediates signal transduction by pheromones during mating
YKL178C	*STE3*	-0.94	Pheromone a-factor G protein-coupled receptor
YLR265C	*NEJ1*	-1.92	Protein involved in nonhomologous end-joining (NHEJ) pathway for repair of DNA double-strand breaks
YMR055C	*BUB2*	-1.01	Checkpoint protein required for cell cycle arrest in response to loss of microtubule function
YNL127W	*FAR11*	-0.70	Component of a high-molecular-weight complex containing Far3p
YNL145W	*MFA2*	-3.74	Mating pheromone a-factor
YOR212W	*STE4*	-2.76	Beta subunit of the trimeric G protein that mediates signal transduction by pheromones
YPL256C	*CLN2*	-0.70	G1/S-specific cyclin
YPR122W	*AXL1*	-1.46	Protease involved in second proteolytic step of a-factor N-terminal processing
Iron metabolism (7)			
YEL065W	*ARN3*	-0.77	Siderophore iron permease
YLR136C	*TIS11*	-1.46	Zinc finger containing protein that belongs to iron regulon
YMR058W	*FET3*	-0.83	Cell surface ferroxidase, required for high-affinity ferrous iron uptake
YOL158C	*ARN4*	-1.29	Protein involved in iron uptake via a siderophore enterobactin
YOR382W	*FIT2*	-2.33	Cell wall mannoprotein of iron transport facilitator
YOR383C	*FIT3*	-1.41	Cell wall mannoprotein of iron transport facilitator
YOR384W	*FRE5*	-0.81	Protein with similarity to Fre2p, ferric reductase
Nucleotide metabolism (9)			
YBR083W	*TEC1*	-1.16	Transcriptional activator, involved with Ste12p in pseudohyphal formation

Gene ID	Gene Name	Average expression	Function / Activity
Nucleotide metabolism (cont'd)			
YBR158W	*AMN1*	-1.02	Protein involved in the AMEN pathway (Antagonist of Mitotic Exit Network) in daughter cells required for exit from mitotic exit and cell cycle reset
YGL073W	*HSF1*	-1.89	Heat shock transcription factor that binds to the heat shock DNA element at both normal and elevated temperatures
YGL248W	*PDE1*	-0.87	3',5'-Cyclic-nucleotide phosphodiesterase
YHL027W	*RIM101*	-1.13	Transcription factor involved in induction of IME1, IME2, DIT1, and DIT2 transcription, has three C2H2-type zinc fingers
YJL130C	*URA2*	-0.16	Multifunctional protein of pyrimidine biosynthesis pathway
YLR256W	*HAP1*	-0.97	Transcription factor with heme-dependent DNA-binding activity, responsible for heme-dependent activation of many genes
YML061C	*PIF1*	-1.63	Single-stranded DNA-dependent ATPase and 5'-3' DNA helicase required for maintenance and repair of mitochondrial DNA, also functions in nucleus to regulate telomere length
YMR287C	*MSU1*	-0.70	Component of a mitochondrial 3'-5' exonuclease complex that is essential for mitochondrial biogenesis
Others (15)			
YBR208C	*DUR1,2*	-1.04	Urea amidolyase, contains urea carboxylase and allophanate hydrolase activities fused together in a single polypeptide
YCL040W	*GLK1*	-1.02	Glucokinase
YDR158W	*HOM2*	-1.04	Aspartate-semialdehyde dehydrogenase, in common pathway for methionine and threonine biosynthesis
YFL059W	*SNZ3*	-1.65	Putative pyridoxine (vitamin B6) biosynthetic enzyme
YJR025C	*BNA1*	-0.71	3-hydroxyanthranilate 3,4-dioxygenase, in biosynthesis of nicotinic acid
YJR159W	*SOR1*	-1.06	Sorbitol dehydrogenase
YKL142W	*MRP8*	-0.99	Mitochondrial ribosomal protein of the small subunit
YMR173W	*DDR4*[illegible]	-1.08	Stress protein induced by heat shock, DNA damage, or osmotic stress
YNL160W	*YGP1*	-1.09	Secreted glycoprotein produced in response to nutrient limitation
YNL333W	*SNZ2*	-1.47	Putative pyridoxine (vitamin B6) biosynthetic enzyme
YNL334C	*SNO2*	-1.60	Putative pyridoxine (vitamin B6) biosynthetic enzyme with similarity to glutamine aminotransferases
YOR136W	*IDH2*	-0.74	Isocitrate dehydrogenase, in the tricarboxylic acid cycle
YPL057C	*SUR1*	-1.00	Protein required for the synthesis of mannosylated sphingolipids
YPL058C	*PDR12*	-1.03	Protein required for weak organic acid resistance, member of the ATP-binding cassette (ABC) superfamily of membrane transporters

Gene ID	Gene Name	Average expression	Function / Activity
Others (cont'd)			
YPL170W	*DAP1*	-3.05	Protein involved in ergosterol biosynthesis, response to UV, mitochondrial genome maintenance, and telomere maintenance, member of the membrane-associated progesterone receptor family
Functionally unknown genes (39)			
YBL005W-B	*YBL005W-B*	-0.87	Unknown
YBL034C	*STU1*	-0.80	Suppressor of beta-tubulin mutation that is required for assembly of the mitotic spindle
YBL101W-A	*YBL101W-A*	-0.79	Unknown
YBL101W-B	*YBL101W-B*	-0.97	Unknown
YBR012C	*YBR012C*	-0.88	Unknown
YBR073W	*RDH54*	-1.26	Protein required for mitotic diploid-specific recombination and repair and for meiosis
YBR157C	*ICS2*	-1.41	Protein required for normal resistance to copper
YBR293W	*YBR293W*	-1.02	Unknown
YBR296C	*PHO89*	-0.80	Na+/Pi symporter (putative)
YCL019W	*YCL019W*	-1.08	Unknown
YCL020W	*YCL020W*	-0.95	Unknown
YDR024W	*FYV1*	-1.53	Unknown
YDR128W	*YDR128W*	-0.82	Unknown
YDR340W	*YDR340W*	-0.92	Unknown
YER138C	*YER138C*	-1.31	Unknown
YER160C	*YER160C*	-1.19	Unknown
YER189W	*YER189W*	-0.80	Unknown
YFL027C	*GYP8*	-0.94	Possible GTPase activating protein
YFL061W	*YFL061W*	-0.85	Unknown
YFL064C	*YFL064C*	-0.85	Unknown
YGL193C	*YGL193C*	-1.02	Unknown
YHR214C-B	*YHR214C-B*	-1.22	Unknown
YJR027W	*YJR027W*	-1.17	Unknown
YJR029W	*YJR029W*	-0.95	Unknown
YLR343W	*YLR343W*	-1.38	Unknown
YLR346C	*YLR346C*	-0.98	Unknown
YML039W	*YML039W*	-0.91	Unknown
YML040W	*YML040W*	-1.28	Unknown
YML045W	*YML045W*	-1.45	Unknown
YMR045C	*YMR045C*	-1.29	Unknown
YMR173W-A	*YMR173W-A*	-1.11	Unknown
YNL078W	*NIS1*	-0.77	Protein that interacts with septins and interacts with Gin4p, Kcc4p, and Nap1p, suggesting a possible role in the mitotic signaling network
YNL155W	*YNL155W*	-0.82	Unknown
YOR072W	*YOR072W*	-0.68	Unknown

Gene ID	Gene Name	Average expression	Function / Activity
Functionally unknown genes (cont'd)			
YOR135C	*YOR135C*	-0.64	Unknown
YOR284W	*HUA2*	-0.71	Protein that may have a role in assembly of cortical actin patches
YOR289W	*YOR289W*	-0.81	Unknown
YPL107W	*YPL107W*	-1.00	Unknown
YPR027C	*YPR027C*	-1.09	Unknown
