# Supplementary material for: Novel insights into iron metabolism by integrating deletome and transcriptome analysis in an iron deficiency model of the yeast Saccharomyces cerevisiae
Source: BMC Genomics. 2009 Mar 25;10:130. doi: 10.1186/1471-2164-10-130 (PMC2669097; doi:10.1186/1471-2164-10-130)
Supplement: Additional file 13 — List of differentially-expressed genes in mrs4Δ. Gene expression profiling was performed in mrs4Δ and wild type strain grown in YPD media. Mrs4p is a mitochondrial iron transporter that functions under low-iron conditions. [file 1471-2164-10-130-S13.pdf]

Additional File 13: List of differentially-expressed genes in the *mrs4Δ* mutant. Genes that were up- or down-regulated in at least two out of three independent experiments are listed and categorized according to their cellular functions using the GO biological process from Funspec. The average expression level is shown in logarithmic scale with base of two.

UP-REGULATED GENES

Gene ID	Gene Name	Average expression	Function / Activity
Carbohydrate and energy metabolism (25)			
YBR126C	*TPS1*	2.20	Trehalose-6-phosphate synthase
YCL040W	*GLK1*	3.52	Glucokinase
YCR005C	*CIT2*	1.66	Citrate synthase
YDL021W	*GPM2*	3.15	Phosphoglycerate mutase
YDL022W	*GPD1*	1.59	Glycerol-3-phosphate dehydrogenase
YDL124W	*YDL124W*	2.49	NAD(P)H-dependent reductase
YDR074W	*TPS2*	1.50	Trehalose-6-phosphate phosphatase
YDR342C	*HXT7*	2.70	High-affinity hexose transporter
YEL011W	*GLC3*	3.31	Glycogen branching enzyme
YER062C	*HOR2*	2.51	DL-glycerol phosphate phosphatase
YER175C	*TMT1*	2.58	Trans-aconitate methyltransferase
YFR015C	*GSY1*	3.15	Glycogen synthetase isoform 1
YFR053C	*HXK1*	2.82	Hexokinase I
YHR044C	*DOG1*	1.66	2-Deoxyglucose-6-phosphate phosphatase
YJL052W	*TDH1*	1.83	Glyceraldehyde-3-phosphate dehydrogenase 1
YKL150W	*MCR1*	1.48	NADH-cytochrome b5 reductase
YKR058W	*GLG1*	2.00	Self-glucosylating initiator of glycogen synthesis
YLR258W	*GSY2*	2.15	Glycogen synthetase isoform 2
YMR011W	*HXT2*	1.45	High-affinity hexose transporter
YMR105C	*PGM2*	3.42	Phosphoglucomutase
YNL037C	*IDH1*	3.09	Isocitrate dehydrogenase subunit 1
YNR001C	*CIT1*	2.67	Citrate synthase
YOL059W	*GPD2*	2.17	Glycerol-3-phosphate dehydrogenase
YOR347C	*PYK2*	1.50	Pyruvate kinase
YPR160W	*GPH1*	3.99	Glycogen phosphorylase
Iron metabolism (11)			
YDR270W	*CCC2*	1.61	Copper-transporting P-type ATPase
YDR534C	*FIT1*	4.60	Cell wall mannoprotein of iron transport facilitator
YHL040C	*ARN1*	2.58	Protein involved in iron uptake via a siderophore
YHL047C	*ARN2*	2.56	Protein involved in iron uptake via a siderophore
YKL220C	*FRE2*	1.56	Ferric and cupric reductase
YLR136C	*TIS11*	2.63	Zinc finger containing protein that belongs to iron regulon
YMR058W	*FET3*	1.73	Cell surface ferroxidase, required for high-affinity ferrous iron uptake

Gene ID	Gene Name	Average expression	Function / Activity
Iron metabolism (cont'd)			
YOL158C	*ENB1; ARN4*	2.42	Protein involved in iron uptake via a siderophore
YOR381W	*FRE3*	1.60	Ferric reductase involved in siderophore iron transport
YOR382W	*FIT2*	4.99	Cell wall mannoprotein of iron transport facilitator
YOR383C	*FIT3*	5.23	Cell wall mannoprotein of iron transport facilitator
Nucleotide metabolism (6)			
YDL048C	*STP4*	2.35	Strong similarity to Stp1p
YDR043C	*NRG1*	1.40	Transcriptional repressor involved in glucose repression
YGL073W	*HSF1*	3.58	Heat shock transcription factor
YHL027W	*RIM101*	3.05	Transcription factor for sporulation related genes
YKL062W	*MSN4*	2.35	Zinc-finger transcriptional activator
YKL109W	*HAP4*	2.06	Transcription factor with acidic activation domain, component of Hap2p-Hap3p-Hap4p-Hap5p complex involved in activation of CCAAT box-containing genes
Others (15)			
YBL034C	*STU1*	1.66	Suppressor of beta-tubulin mutation
YBR046C	*ZTA1*	2.04	Zeta-crystallin homolog
YDL234C	*GYP7*	1.72	GTPase-activating protein for Ypt7p
YER024W	*YAT2*	2.10	Protein with carnitine acetyltransferase function
YGR008C	*STF2*	3.50	ATPase stabilizing factor
YHR053C	*CUP1A*	3.34	Copper metallothionein
YLR178C	*TFS1*	2.82	Cell cycle regulator
YLR286C	*CTS1*	1.64	Endochitinase
YMR008C	*PLB1*	1.66	Phospholipase B
YNL145W	*MFA2*	3.36	Mating pheromone a-factor
YOR036W	*PEP12*	1.43	Syntaxin homolog
YOR360C	*PDE2*	1.43	3',5'-Cyclic-nucleotide phosphodiesterase
YPL087W	*YDC1*	1.64	Alkaline ceramidase
YPL154C	*PEP4*	2.00	Proteinase A
YPL171C	*OYE3*	1.42	NADPH dehydrogenase isoform 3
Protein metabolism (8)			
YCL064C	*CHA1*	1.40	L-serine/L-threonine deaminase
YDR059C	*UBC5*	1.73	Ubiquitin-conjugating enzyme
YDR247W	*YDR247W*	1.74	Serine/threonine protein kinase
YER098W	*UBP9*	1.81	Ubiquitin C-terminal hydrolase
YJR148W	*BAT2*	2.13	Branched-chain amino acid transaminase
YKL103C	*LAP4*	2.24	Aminopeptidase I
YKL142W	*MRP8*	2.08	Ribosomal protein of the small subunit
YKR093W	*PTR2*	1.65	Peptide permease
Stress response (9)			
YBR054W	*YRO2*	3.35	Protein paralog of Mrh1p
YCR021C	*HSP30: YRO1*	4.28	Heat shock protein
YDR171W	*HSP42*	2.47	Heat shock protein
YDR258C	*HSP78*	1.86	Heat shock protein
YFL014W	*HSP12*	3.61	Heat shock protein
YGL037C	*PNC1*	2.14	Pyrazinamidase and nicotinamidase
YIR038C	*GTT1*	1.72	Glutathione transferase
YKL026C	*GPX1*	1.60	Glutathione peroxidase
YMR173W	*DDR48*	2.19	Induced by heat shock, DNA damage, or osmotic stress
Transporters (4)			
YER053C	*YER053C*	3.82	Member of the mitochondrial carrier family of membrane transporters

Gene ID	Gene Name	Average expression	Function / Activity
Transporters (cont'd)			
YGR138C	*TPO2*	1.80	Polyamine transport protein
YOR153W	*PDR5*	2.07	Drug-efflux pump involved in resistance to multiple drugs
YPR036W	*VMA13*	1.53	Vacuolar H(+)-ATPase
Functionally unknown genes (30)			
YBR047W	*YBR047W*	2.68	Unknown
YCL042W	*YCL042W*	2.81	Unknown
YCL049C	*YCL049C*	1.63	Unknown
YDL023C	*YDL023C*	2.03	Unknown
YDL169C	*UGX2*	3.25	Unknown
YDR055W	*PST1*	2.50	Member of the Sps2p-Ecm33p-Ycl048p family
YDR391C	*YDR391C*	2.34	Unknown
YER067W	*YER067W*	4.97	Unknown
YFL020C	*PAU5*	2.28	Member of the seripauperin (PAU) family
YHR016C	*YSC84*	2.19	Protein involved in cortical actin patch polarization with Lsb5p
YJL079C	*PRY1*	2.32	Protein with similarity to plant pathenogenesis-related proteins
YKR075C	*YKR075C*	1.92	Unknown
YLR297W	*YLR297W*	1.78	Unknown
YLR327C	*YLR327C*	4.70	Protein with high similarity to S. cerevisiae Stf2p
YLR346C	*YLR346C*	2.72	Unknown
YMR020W	*FMS1*	1.39	Protein involved in the biosynthesis of pantothenic acid
YMR173W-A	*YMR173W-A*	2.09	Unknown
YMR181C	*YMR181C*	2.31	Unknown
YMR251W-A	*HOR7*	2.23	Protein involved in responsiveness to hyperosmolarity
YMR315W	*YMR315W*	1.48	Unknown
YNL160W	*YGP1*	5.44	Secreted glycoprotein produced in response to nutrient limitation
YNL200C	*YNL200C*	2.34	Unknown
YNL208W	*YNL208W*	1.92	Unknown
YOL163W	*YOL163W*	1.87	Unknown
YOR135C	*YOR135C*	2.30	Unknown
YOR285W	*YOR285W*	1.62	Unknown
YOR289W	*YOR289W*	2.74	Unknown
YOR338W	*YOR338W*	3.42	Unknown
YPL004C	*YPL004C*	1.83	Protein with weak similarity to tropomyosin
YPL250C	*YPL250C*	2.21	Unknown

DOWN-REGULATED GENES

Gene ID	Gene Name	Average expression	Function / Activity
Nucleotide metabolism (3)			
YGR109C	*CLB6*	-1.89	Involved in initiation of DNA synthesis
YJL033W	*HCA4*	-1.42	Involved in ribosomal RNA processing
YMR260C	*TIF11*	-1.64	Translation initiation factor eIF1A
Others (7)			
YAR071W	*PHO11*	-1.68	Acid phosphatase
YBR093C	*PHO5*	-2.00	Acid phosphatase
YHR215W	*PHO12*	-1.81	Acid phosphatase
YIL011W	*TIR3*	-0.16	Member of the seripauperin family
YKR052C	*MRS4*	-2.62	Mitochondrial iron transporter
YNL111C	*CYB5*	-1.62	Cytochrome b5
YPL060W	*LPE10; (MRS7)*	-0.67	Involved in mitochondrial magnesium homeostasis
Protein metabolism (4)			
YDR234W	*LYS4*	-1.62	Homoaconitate hydratase
YGL009C	*LEU1*	-2.78	3-Isopropylmalate dehydratase
YHR208W	*BAT1*	-1.95	Mitochondrial branched-chain amino acid transaminase
YMR230W	*RPS10B*	-1.27	Ribosomal protein S10
Functionally unknown genes (6)			
YDR281C	*PHM6*	-1.44	Predicted to have a role in phosphate metabolism
YEL033W	*YEL033W*	-1.99	Required for invasive growth and pseudohyphal development
YER156C	*YER156C*	-1.54	Unknown
YGR164W	*YGR164W*	-1.55	Unknown
YLR205C	*HMX1*	-0.02	Unknown
YOR237W	*HES1*	-0.66	Protein implicated in ergosterol biosynthesis
