# Supplementary material for: Novel insights into iron metabolism by integrating deletome and transcriptome analysis in an iron deficiency model of the yeast Saccharomyces cerevisiae
Source: BMC Genomics. 2009 Mar 25;10:130. doi: 10.1186/1471-2164-10-130 (PMC2669097; doi:10.1186/1471-2164-10-130)
Supplement: Additional file 14 — List of differentially-expressed genes in the yhr045wΔ. Gene expression profiling was performed in yhr045wΔ and wild type strain grown in YPD media. Yhr045wp is a putative protein of unknown function. [file 1471-2164-10-130-S14.pdf]

Additional File 14: List of differentially-expressed genes in the *yhr045wΔ* mutant. Genes that were up- or down-regulated in at two independent experiments are listed and categorized according to their cellular functions using the GO biological process from Funspec. The average expression level is shown in logarithmic scale with base of two.

UP-REGULATED GENES

Gene ID	Gene Name	Average expression	Function
Carbohydrate metabolism and energy metabolism (23)			
YAL060W	*BDH1*	1.17	Butanediol dehydrogenase
YBR126C	*TPS1*	1.18	Trehalose-6-phosphate synthase
YBR149W	*ARA1*	1.19	Arabinose dehydrogenase
YCL040W	*GLK1*	1.57	Glucokinase, specific for aldohexoses
YCR005C	*CIT2*	1.72	Citrate synthase
YDL021W	*GPM2*	2.30	Phosphoglycerate mutase
YDL022W	*GPD1*	0.98	Glycerol-3-phosphate dehydrogenase
YDR074W	*TPS2*	1.00	Trehalose-6-phosphate phosphatase
YEL071W	*DLD3*	1.18	D-lactate dehydrogenase
YER178W	*PDA1*	0.87	Pyruvate dehydrogenase complex alpha subunit
YFR053C	*HXK1*	1.76	Hexokinase I
YGL156W	*AMS1*	1.66	Alpha-Mannosidase
YIL070C	*MAM33*	0.91	Mitochondrial acidic matrix protein
YJL052W	*TDH1*	0.96	Glyceraldehyde-3-phosphate dehydrogenase 1
YKL085W	*MDH1*	1.03	Malate dehydrogenase, mitochondrial
YKL150W	*MCR1*	0.95	NADH-cytochrome b5 reductase
YLR304C	*ACO1*	1.83	Aconitate hydratase
YMR105C	*PGM2*	1.88	Phosphoglucomutase
YMR145C	*NDE1*	1.28	Mitochondrial NADH dehydrogenase
YNR001C	*CIT1*	1.72	Citrate synthase
YOL136C	*PFK27*	0.93	6-Phosphofructose-2-kinase
YOR136W	*IDH2*	2.74	Isocitrate dehydrogenase subunit 2
YOR347C	*PYK2*	1.30	Pyruvate kinase
Iron metabolism (8)			
YDR270W	*CCC2*	1.55	Copper-transporting P-type ATPase
YEL065W	*SIT1*	1.97	Siderophore iron permease
YFL041W	*FET5*	1.50	Multicopper oxidase involved in ferrous iron transport
YLR136C	*TIS11*	3.03	Zinc finger containing protein that belongs to iron regulon
YOL158C	*ENB1*	1.98	Siderophore iron permease
YOR382W	*FIT2*	4.26	Cell wall mannoprotein of iron transport facilitator
YOR383C	*FIT3*	4.76	Cell wall mannoprotein of iron transport facilitator
YOR384W	*FRE5*	1.87	Ferric reductase homologue
Lipid metabolism (7)			
YDR058C	*TGL2*	0.97	Triglyceride lipase
YGR175C	*ERG1*	1.12	Squalene monooxygenase
YLR056W	*ERG3*	1.36	C-5 sterol desaturase
YML008C	*ERG6*	0.97	S-adenosylmethionine delta-24-sterol-C-methyltransferase
YMR008C	*PLB1*	1.00	Phospholipase B

Gene ID	Gene Name	Average expression	Function
Lipid metabolism (cont'd)			
YOR049C	*RSB1*	3.51	Involved in the release of sphingoid long-chain bases
YOR237W	*HES1*	3.16	Protein implicated in ergosterol biosynthesis
Metal homeostasis (2)			
YGR257C	*MTM1*	0.95	Putative metal chaperone for Sod2p
YHR053C	*CUP1A*	1.73	Metallothionein (copper chelatin)
Nucleotide metabolism (14)			
YBL005W	*PDR3*	1.89	Zinc-finger transcription factor
YBR066C	*NRG2*	0.89	Suppressor of snf mutations
YCL061C	*MRC1*	1.02	Component of the replication fork
YEL066W	*HPA3*	1.31	Histone and other protein acetyltransferase
YER035W	*EDC2*	1.62	Protein involved in mRNA decapping
YGL037C	*PNC1*	1.30	Protein with pyrazinamidase and nicotinamidase activity
YHL027W	*RIM101*	3.08	Transcription factor for sporulation relatad genes
YHR006W	*STP2*	1.37	Transcription factor regulating amino acid permease genes
YIR018W	*YAP5*	1.30	Transcription factor of the basic leucine zipper type
YKL109W	*HAP4*	1.50	Transcription factor with acidic activation domain
YOR344C	*TYE7*	1.12	Basic helix-loop-helix transcription factor
YOR360C	*PDE2*	0.83	3',5'-Cyclic-nucleotide phosphodiesterase
YOR363C	*PIP2*	1.20	Transcription factor required for induction of peroxisomal proteins
YPL086C	*ELP3*	1.32	Subunit of the RNA Polymerase II elongator holoenzyme
Others (34)			
YBL034C	*STU1*	1.34	Suppressor of beta-tubulin mutation
YBL043W	*ECM13*	2.42	Possibly involved in cell wall structure or biosynthesis
YBL078C	*AUT7*	1.15	Delivery of autophagic vesicles
YBR105C	*VID24*	2.23	Vacuolar protein targeting
YBR183W	*YPC1*	1.39	Alkaline ceramidase
YDL124W	*YDL124W*	1.61	NAD(P)H-dependent reductase
YDL234C	*GYP7*	1.24	GTPase-activating protein for Ypt7p
YDR011W	*SNQ2*	1.25	Drug-efflux pump (ATP-binding cassette superfamily)
YDR072C	*IPT1*	0.89	Inositolphosphotransferase 1
YER024W	*YAT2*	1.95	Carnitine acetyltransferase function
YER044C-A	*MEI4*	1.00	Protein required early in meiosis
YER124C	*DSE1*	1.27	Protein involved in cell wall metabolism
YER175C	*TMT1*	2.19	Trans-aconitate methyltransferase
YGL086W	*MAD1*	0.82	Protein involved in spindle-assembly checkpoint
YGR008C	*STF2*	1.46	ATPase stabilizing factor
YGR141W	*VPS62*	0.84	Class F vacuolar protein sorting protein
YGR239C	*PEX21*	1.16	Peroxisomal biogenesis protein
YHR046C	*INM1*	1.62	Inositol monophosphatase
YHR179W	*OYE2*	1.01	NADPH dehydrogenase
YIR038C	*GTT1*	1.39	Glutathione transferase
YKL008C	*LAC1*	1.25	Longevity-assurance gene 1 cognate
YKL026C	*GPX1*	1.09	Glutathione peroxidase
YKR053C	*YSR3*	1.12	Sphingoid base-phosphate phosphatase
YLR178C	*TFS1*	2.19	Carboxypeptidase Y inhibitor
YLR286C	*CTS1*	1.40	Endochitinase
YMR316W	*DIA1*	1.51	Protein involved in invasive growth
YNL192W	*CHS1*	0.86	Chitin synthase I
YNL242W	*APG2*	1.04	Vacuolar protein targeting
YOR036W	*PEP12*	0.8	Syntaxin homolog involved in Golgi to vacuole transport

Gene ID	Gene Name	Average expression	Function
Others (cont'd)			
YOR153W	*PDR5*	2.83	Drug-efflux pump (ATP-binding cassette superfamily)
YOR185C	*GSP2*	1.14	GTP-binding protein member of the ras superfamily
YPL059W	*GRX5*	1.05	Glutaredoxin
YPL087W	*YDC1*	1.23	Alkaline ceramidase
YPR149W	*NCE102*	0.94	Involved in secretion of proteins that lack secretory signal
Protein metabolism (13)			
YDR158W	*HOM2*	0.90	Aspartate-semialdehyde dehydrogenase
YHR038W	*RRF1*	0.83	Mitochondrial ribosome recycling factor
YHR047C	*AAP1*	1.54	Alanine/arginine aminopeptidase
YIL095W	*PRK1*	1.59	Serine/threonine protein kinase
YJL088W	*ARG3*	1.18	Ornithine carbamyltransferase
YJR148W	*BAT2*	1.62	Cytosolic branched-chain amino acid transaminase
YKL103C	*LAP4*	1.15	Aminopeptidase I of the vacuole
YKL142W	*MRP8*	1.65	Mitochondrial ribosomal protein
YLR120C	*YPS1*	0.91	Yapsin 1, GPI-anchored protease
YLR303W	*MET17*	1.46	O-Acetylhomoserine sulfhydrylase
YNL015W	*PBI2*	1.28	Protease B
YOR303W	*CPA1*	1.38	Carbamoylphosphate synthetase
YPL154C	*PEP4*	1.61	Proteinase A
Stress response (9)			
YDR171W	*HSP42*	1.28	Heat shock protein
YDR258C	*HSP78*	1.35	Mitochondrial heat shock protein
YFL014W	*HSP12*	4.17	Heat shock protein
YGL073W	*HSF1*	3.71	Heat shock transcription factor
YMR173W	*DDR48*	1.68	Induced by heat shock, DNA damage, or osmotic stress
YMR251W-A	*HOR7*	1.26	Protein involved in responsiveness to hyperosmolarity
YNL160W	*YGP1*	3.86	Secreted glycoprotein in response to nutrient limitation
YOL053C-A	*DDR2*	1.99	DNA damage responsive protein
YOL151W	*GRE2*	1.88	Alpha-Acetoxy ketone reductase
Transporters (7)			
YAL067C	*SEO1*	1.19	Member of the allantoate permease family
YHL035C	*YHL035C*	1.45	Member of the ATP-binding cassette (ABC) superfamily
YJR150C	*DAN1*	2.19	Cell wall mannoprotein
YKR093W	*PTR2*	1.19	Peptide permease
YNL055C	*POR1*	1.07	Outer mitochondrial membrane porin
YOR306C	*MCH5*	0.94	Monocarboxylate permease homologue
YPR156C	*TPO3*	0.80	Polyamine transport protein
Functionally unknown genes (90)			
YAL061W	*YAL061W*	3.31	Member of the zinc-containing alcohol dehydrogenase family
YAL068C	*YAL068C*	1.40	Possibly required for full induction of IME1 during early meiosis
YAR020C	*PAU7*	1.64	Member of the seripauperin family
YBL049W	*MOH1*	1.42	Unknown
YBR005W	*YBR005W*	1.02	Unknown
YBR046C	*ZTA1*	1.15	Zeta-crystallin homolog
YBR047W	*YBR047W*	2.12	Unknown
YBR269C	*YBR269C*	0.99	Unknown
YCL049C	*YCL049C*	0.97	Unknown
YDL023C	*YDL023C*	1.37	Unknown
YDL038C	*YDL038C*	1.35	Unknown

Gene ID	Gene Name	Average expression	Function
Functionally unknown genes (cont'd)			
YDL048C	*STP4*	1.59	Similarity to Stp1p
YDL110C	*YDL110C*	0.81	Unknown
YDL169C	*UGX2*	1.73	Unknown
YDR032C	*PST2*	1.09	Similarity to Ycp4p
YDR055W	*PST1*	1.25	Member of the Sps2p-Ecm33p-Ycl048p family
YDR264C	*AKR1*	1.09	Protein with palmitoyl transferase activity
YDR271C	*YDR271C*	1.63	Unknown
YDR516C	*EMI2*	0.95	Required for full induction of IME1 during early meiosis
YEL049W	*PAU2*	1.28	Similarity to S. cerevisiae Yal068p
YEL067C	*YEL067C*	1.09	Unknown
YER038C	*KRE29*	1.69	Unknown
YER053C	*YER053C*	2.47	Member of the mitochondrial carrier family of membrane transporters
YER079W	*YER079W*	1.70	Unknown
YFL020C	*PAU5*	1.40	Member of the seripauperin (PAU) family
YFL067W	*YFL067W*	1.10	Unknown
YFR017C	*YFR017C*	1.73	Unknown
YGL157W	*YGL157W*	1.69	Unknown
YGL260W	*YGL260W*	0.97	Unknown
YGR017W	*YGR017W*	0.97	Unknown
YGR131W	*YGR131W*	1.19	Unknown
YGR146C	*YGR146C*	1.01	Unknown
YGR161C	*RTS3*	1.94	Unknown
YGR213C	*RTA1*	1.38	Involved in 7-aminocholesterol resistance
YHR016C	*YSC84*	0.97	Protein involved in cortical actin patch polarization with Lsb5p
YHR070W	*TRM5*	1.29	Unknown
YHR113W	*YHR113W*	1.62	Unknown
YHR138C	*YHR138C*	1.21	Unknown
YHR140W	*YHR140W*	0.92	Unknown
YIL015C-A	*YIL014C-A*	1.03	Unknown
YJL066C	*MPM1*	0.96	Unknown
YJL067W	*YJL067W*	0.98	Unknown
YJL079C	*PRY1*	1.38	Protein with similarity to plant pathenogenesis-related proteins
YJL116C	*NCA3*	1.58	Protein involved in regulation of synthesis of the Fo-F1 ATP synthase
YJL161W	*YJL161W*	1.43	Unknown
YKL071W	*YKL071W*	1.10	Unknown
YKL151C	*YKL151C*	1.51	Unknown
YKR076W	*ECM4*	1.39	Unknown
YLL019C	*KNS1*	1.26	Serine/threonine protein kinase
YLL025W	*YLL025W*	1.38	Unknown
YLL056C	*YLL056C*	2.01	Unknown
YLR037C	*DAN2*	1.66	Member of the seripauperin (PAU) family
YLR089C	*YLR089C*	0.96	Putative mitochondrial alanine aminotransferase
YLR099C	*ICT1*	1.39	Required for normal sensitivity to copper
YLR152C	*YLR152C*	1.16	Unknown
YLR194C	*YLR194C*	1.46	GPI-anchored protein
YLR270W	*DCS1*	1.31	Trehalase-associated protein
YLR297W	*YLR297W*	1.43	Unknown

Gene ID	Gene Name	Average log_2 expression	Function
Functionally unknown genes (cont'd)			
YLR327C	*YLR327C*	3.10	Protein with high similarity to S. cerevisiae Stf2p
YLR345W	*YLR345W*	1.02	Similarity to fructose-2,6-bisphosphatases
YLR346C	*YLR346C*	3.34	Unknown
YML047C	*PRM6*	0.96	Pheromone-Regulated Membrane protein
YMR041C	*YMR041C*	0.85	Protein containing an aldo-keto reductase family domain
YMR095C	*SNO1*	4.50	Putative pyridoxine biosynthetic enzyme
YMR096W	*SNZ1*	3.20	Putative pyridoxine biosynthetic enzyme
YMR102C	*YMR102C*	2.63	Unknown
YMR134W	*YMR134W*	0.87	Unknown
YMR173W-A	*YMR173W-A*	1.74	Unknown
YMR195W	*ICY1*	1.58	Unknown
YMR244C-A	*YMR244C-A*	1.38	Unknown
YMR245W	*YMR245W*	1.04	Unknown
YMR291W	*YMR291W*	1.15	Serine/threonine protein kinase of unknown function
YMR316C-A	*YMR316C-A*	1.40	Unknown
YNL040W	*YNL040W*	0.92	Unknown
YNL200C	*YNL200C*	1.28	Unknown
YNL208W	*YNL208W*	1.22	Unknown
YNL234W	*YNL234W*	1.02	Hemoprotein with similarity to mammalian globins
YOL150C	*YOL150C*	1.78	Unknown
YOL161C	*YOL161C*	1.22	Member of the seripauperin (PAU) family
YOR135C	*YOR135C*	3.01	Required for full induction of IME1 during early meiosis
YOR137C	*SIA1*	0.95	Suppressor of eIF5A
YOR173W	*DCS2*	1.31	Protein with high similarity to S. cerevisiae Dcs1p
YOR289W	*YOR289W*	2.31	Unknown
YOR338W	*YOR338W*	3.73	Possibly required for full induction of IME1 during early meiosis
YOR394W	*YOR394W*	1.81	Similarity to S. cerevisiae Yir041p
YPL159C	*YPL159C*	0.93	Unknown
YPL250C	*ICY2*	1.64	Unknown
YPL272C	*YPL272C*	2.32	Unknown
YPL282C	*YPL282C*	1.40	Similarity to S. cerevisiae Yir041p
YPR002W	*PDH1*	2.30	Involved in propionate utilization

DOWN-REGULATED GENES

Gene ID	Gene Name	Average expression	Function
Nucleotide metabolism (9)			
YBR021W	*FUR4*	-0.98	Uracil permease
YDR216W	*ADR1*	-0.89	Zinc-finger transcription factor
YDR399W	*HPT1*	-1.05	Hypoxanthine-guanine phosphoribosyl transferase
YER060W-A	*FCY22*	-1.35	Purine/cytosine permease
YGL241W	*KAP114*	-3.09	Protein of the karyopherin-beta family
YGR109C	*CLB6*	-1.18	B-type cyclin appearing late in G1
YLR359W	*ADE13*	-1.04	Adenylosuccinate lyase
YLR449W	*FPR4*	-0.88	Nucleolar peptidylprolyl cis-trans isomerase
YPR065W	*ROX1*	-1.56	Heme-dependent transcriptional repressor
Others (8)			
YBR092C	*PHO3*	-1.28	Acid phosphatase
YBR093C	*PHO5*	-1.43	Acid phosphatase
YBR244W	*GPX2*	-1.17	Glutathione peroxidase
YER174C	*GRX4*	-0.93	Glutaredoxin
YHR051W	*COX6*	-0.86	Cytochrome c oxidase subunit VI
YHR096C	*HXT5*	-3.21	Glucose transporter
YKL096W	*CWP1*	-0.97	Mannoprotein of the cell wall
YPL274W	*SAM3*	-1.48	High affinity S-adenosylmethionine permease
Protein metabolism (22)			
YDL184C	*RPL41A*	-1.35	Ribosomal protein L41A
YDR382W	*RPP2B*	-0.86	Acidic ribosomal protein P2B
YGL009C	*LEU1*	-1.04	3-Isopropylmalate dehydratase
YGR034W	*RPL26B*	-1.32	Ribosomal protein L26
YHL001W	*RPL14B*	-0.91	Ribosomal protein L14
YHR010W	*RPL27A*	-0.94	Ribosomal protein L27
YHR021C	*RPS27B*	-1.06	Ribosomal protein S27
YJL136C	*RPS21B*	-1.02	Ribosomal protein S21
YJL189W	*RPL39*	-1.02	Ribosomal protein L39
YJR094W-A	*RPL43B*	-1.04	Ribosomal protein L43B
YKR057W	*RPS21A*	-1.12	Ribosomal protein S21
YLR167W	*RPS31*	-1.10	Ribosomal protein S31
YLR264W	*RPS28B*	-1.07	Ribosomal protein S28
YLR325C	*RPL38*	-1.31	Ribosomal protein L38
YLR344W	*RPL26A*	-1.23	Ribosomal protein L26
YML024W	*RPS17A*	-0.90	Ribosomal protein S17
YML026C	*RPS18B*	-1.05	Ribosomal protein S18
YMR230W	*RPS10B*	-1.27	Ribosomal protein S10
YOR096W	*RPS7A*	-1.29	Ribosomal protein S7
YOR167C	*RPS28A*	-1.40	Ribosomal protein S28
YPL143W	*RPL33A*	-1.07	Ribosomal protein L33
YPR167C	*MET16*	-2.32	3'-Phosphoadenylylsulfate reductase
Functionally unknown genes (27)			
YAR070C	*YAR070C*	-1.34	Unknown
YBR108W	*YBR108W*	-1.40	Unknown
YCR102C	*YCR102C*	-1.22	Similarity to Bacillus subtilis sorbitol dehydrogenase
YDL241W	*YDL241W*	-1.18	Unknown
YDR111C	*YDR111C*	-0.93	Similarity to S. cerevisiae Ylr089p

Gene ID	Gene Name	Average expression	Function
Functionally unknown genes (cont'd)			
YEL033W	*YEL033W*	-1.10	Required for invasive growth and pseudohyphal development
YGL010W	*YGL010W*	-0.80	Unknown
YGR043C	*YGR043C*	-2.93	Involved in signal transduction
YGR164W	*YGR164W*	-1.56	Unknown
YGR270W	*YTA7*	-1.58	Member of the AAA ATPase protein family
YHL037C	*YHL037C*	-1.57	Unknown
YIL025C	*YIL025C*	-2.51	Unknown
YIL146C	*ECM37*	-1.53	Possibly involved in cell wall structure
YIR014W	*YIR014W*	-1.15	Unknown
YJL188C	*BUD19*	-0.94	Involved in bud site selection
YJL200C	*YJL200C*	-0.96	Protein with similarity to aconitase
YLL014W	*YLL014W*	-0.83	Unknown
YMR321C	*YMR321C*	-0.99	Unknown
YNL109W	*YNL109W*	-1.19	Unknown
YNL303W	*YNL303W*	-1.39	Unknown
YOL109W	*YOL109W*	-0.86	Unknown
YOL155C	*YOL155C*	-1.06	Similarity to S. cerevisiae glucan 1,4-alpha-glucosidase
YOR271C	*YOR271C*	-0.87	Member of the mitochondrial tricarboxylate carrier family
YOR366W	*YOR366W*	-1.92	Unknown
YPL263C	*KEL3*	-0.81	Protein containing three Kelch motifs
YPR044C	*YPR044C*	-0.94	Unknown
YPR077C	*YPR077C*	-1.90	Unknown
