# Supplementary material for: Novel insights into iron metabolism by integrating deletome and transcriptome analysis in an iron deficiency model of the yeast Saccharomyces cerevisiae
Source: BMC Genomics. 2009 Mar 25;10:130. doi: 10.1186/1471-2164-10-130 (PMC2669097; doi:10.1186/1471-2164-10-130)

Novel insights into iron metabolism by integrating deletome and transcriptome analysis in an iron deficiency model of the yeast *Saccharomyces cerevisiae*

Jo, Kim, Oh, *et al.* (2009)

Additional File 15: Yeast iron map organized by the force-directed layout in Cytoscape.

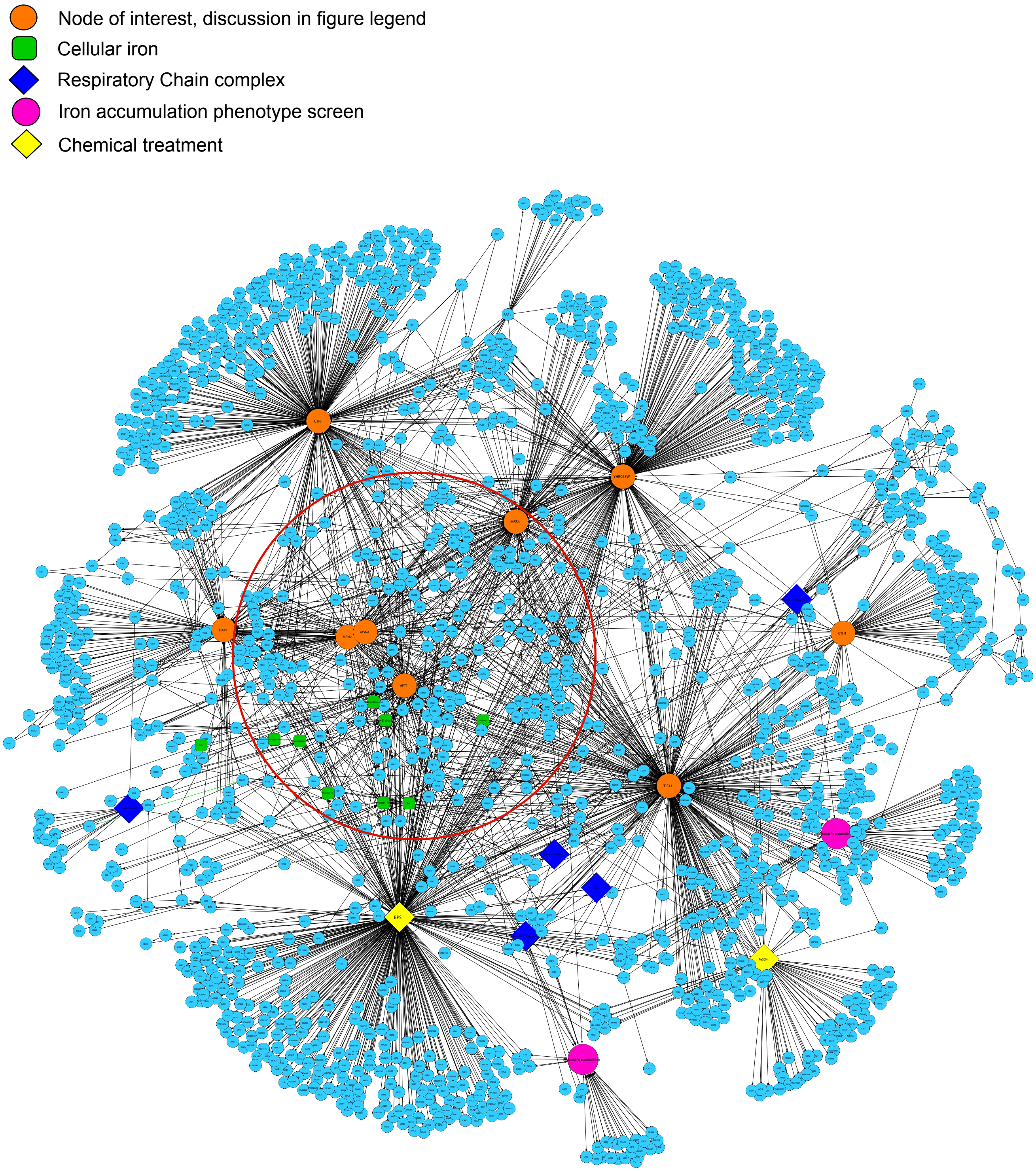

Supplement: Additional file 15 — Yeast iron map organized by the force-directed layout in Cytoscape. Map was constructed using all molecular interactions associated to iron metabolism compiled from the literature, including ones from the present study. The iron metabolism cluster, enriched with known genes associated to it, is circled in red. Genes in this cluster are highly interconnected with each other by edges that represent different molecular interactions retrieved from the literature. Specific genes can be searched in the map by using the Find feature in Adobe Reader or Acrobat. [file 1471-2164-10-130-S15.pdf]
