# Supplementary material for: Novel insights into iron metabolism by integrating deletome and transcriptome analysis in an iron deficiency model of the yeast Saccharomyces cerevisiae
Source: BMC Genomics. 2009 Mar 25;10:130. doi: 10.1186/1471-2164-10-130 (PMC2669097; doi:10.1186/1471-2164-10-130)
Supplement: Additional file 16 — Yeast iron map organized by iron-related pathway and Gene Ontology cellular localization. Map was constructed using molecular interactions associated to iron metabolism compiled from the literature, excluding those ones obtained from genomic screens. The map can be browsed from the main menu by cellular component or iron-related pathway. Specific genes can be searched in the map by using the Find feature in Adobe Reader or Acrobat. [file 1471-2164-10-130-S16.pdf]

**Yeast Iron Map.** Jo, Kim, Oh *et al.* (2009). Novel insights into iron metabolism by integrating deletome and transcriptome analysis in an iron deficiency model of the yeast *Saccharomyces cerevisiae*

**Go to map**

## Cellular Component (click to view on map)

Cell wall
Cellular bud
Cytoplasm
Endoplasmic Reticulum
Endosome
Endosome and cytoplasmic membrane-bounded vesicle
Golgi apparatus
Membrane
Mitochondrion 1
Mitochondrion 2
Mitochondrion 3
Nucleolus
Nucleus
Other
Peroxisome
Plasma membrane
Ribosome
Unknown
Vacuole 1
Vacuole 2

## Pathway (click to view on map)

Biotine biosynthesis
*De novo* biosynthesis of purine nucleotides
*De novo* biosynthesis of pyrimidine ribonucleotides
Electron transport chain
Ergosterol pathway
Galactose metabolism
Glutamate biosynthesis
Glycolytic pathways
Heme biosynthesis
Histidine biosynthesis
Leucine, isoleucine and valine biosynthesis
Lysine biosynthesis
Sulfur amino acid biosynthesis
TCA cycle

## Legend

Functional data - gene deletion induces sensitivity to BPS
Functional data - gene deletion induces resistance to BPS
Physical interaction (PI)
Metal transport (MT/PMT)
Metal transfer expected (MTE)
Transcriptional down regulation (TRD)
Indirect transcriptional down regulation (ITRD)
Transcriptional up regulation (TRU)
Indirect transcriptional up regulation (ITRU)
Transcriptional regulation (TR)
Expression data - gene is up regulated in BPS
Expression data - gene is down regulated in BPS
Transcriptional regulation expected (TRE)
Indirect regulation expected (IRE)
Pathway sequence / step (PS)
Unknown pathway sequence / step (UPS)
Regulatory stimulus (REG)
Involved in FeS cluster assembly (FeS)
Translational activator (TLA)
Activation by phosphorylation (AP)
Genes within respiratory chain complex, or involved in maturation or assembly (RC)

Novel insights into iron metabolism by integrating deletome and transcriptome analysis in an iron deficiency model of the yeast *Saccharomyces cerevisiae*
Jo, Kim, Oh, *et al.* (2009)

Go Back
Legend
View by Cellular Component
View by Pathway

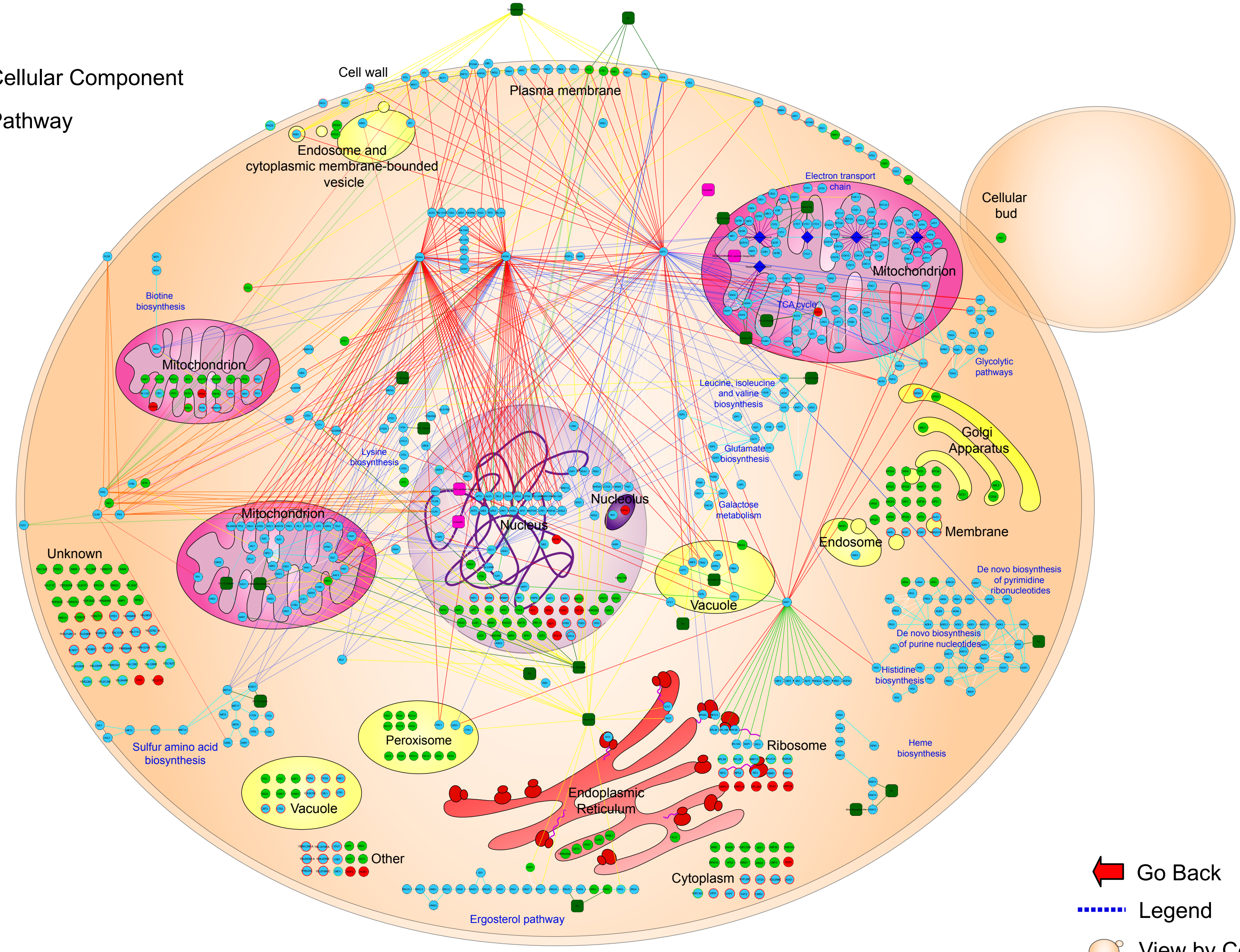


Go Back
Legend
View by Cellular Component
View by Pathway
